# C1′‐Branched Acyclic Nucleoside Phosphonates as Inhibitors of Plasmodium Falciparum 6‐Oxopurine Phosphoribosyltransferase

**DOI:** 10.1002/cmdc.202500575

**Published:** 2025-08-22

**Authors:** Jan Frydrych, Dianne T. Keough, Haojun Xia, Lenka Poštová Slavětínská, Martin Dračínský, Michal Česnek, Jye Travis, Marina Chavchich, Michael Edstein, Dana Hocková, Luke W. Guddat, Zlatko Janeba

**Affiliations:** ^1^ Institute of Organic Chemistry and Biochemistry of the Czech Academy of Sciences Flemingovo nám. 2 160 00 Prague 6 Czech Republic; ^2^ School of Chemistry and Molecular Biosciences The University of Queensland Brisbane 4072 Australia; ^3^ Department of Drug Evaluation Australian Defence Force Malaria and Infectious Disease Institute Enoggera Brisbane Queensland 4051 Australia

**Keywords:** acyclic nucleoside phosphonates, docking, hypoxanthine‐guanine‐(xanthine) phosphoribosyltransferase, inhibitors, malaria, *Plasmodium falciparum*

## Abstract

Hypoxanthine‐guanine‐(xanthine) phosphoribosyltransferase [HG(X)PRT] is an excellent target for the development of new drugs to treat parasitic and bacterial infections as well as MYC‐dependent triple‐negative breast cancer. Inhibitors include compounds that mimic the transition state of the catalytic reaction and analogs of the two products of the reaction, the nucleoside monophosphates and pyrophosphate. One type of chemistry explored here is the design of purine‐based C1′‐branched acyclic nucleoside phosphonates bearing diverse structural attachments (secondary linkers) on the C1′ atom. Compounds where this secondary linker has either a terminal phosphonate or a hydroxyl group are submicromolar to single‐digit micromolar inhibitors of human hypoxanthine‐guanine phosphoribosyltransferase and *Plasmodium falciparum* HGXPRT. The lowest *K*_i_ values for two of these inhibitors are 0.7 µM for the human enzyme and 0.4 µM for the parasite enzyme. The *K*_*i*_ values of the prepared derivatives, however, cover a wide range and depend on the chemical structure of the attachment at the C1′ atom. A phosphonodiamidate prodrug of one of the compounds has an IC_50_ of 4.3  µM against a drug‐sensitive strain of *Plasmodium falciparum* grown in human erythrocytes, showing in vitro activity and the merit of these new inhibitors as potential drug leads.

## Graphical abstract

Novel C1'‐branched acyclic nucleoside phosphonates were synthesized. The compounds are inhibitors of *Plasmodium falciparum* and human 6‐oxopurine phosphoribosyltransferases with *K*_i_ values as low as 0.4 µM and 0.7 µM, respectively. A phosphonodiamidate prodrug exhibited IC_50_ = 4.3 µM against a drug‐sensitive strain of *P. falciparum* grown in human erythrocytes.

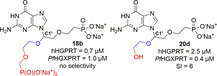

## 1 Introduction

**Figure 1:**
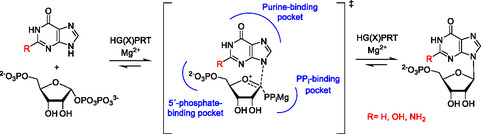
The reaction catalyzed by HG(X)PRT. R = H (hypoxanthine); R = NH_2_ (guanine); R = OH (xanthine). The products of the reaction are IMP, GMP or XMP. The key binding pockets for the substrates/products are highlighted in blue.

Hypoxanthine‐guanine‐(xanthine) phosphoribosyltransferase [HG(X)PRT] catalyzes the reaction between 6‐oxopurines (i.e.*,* hypoxanthine, guanine, or xanthine) and 5‐phospho‐*α*‐d‐ribosyl‐1‐pyrophosphate (PRPP) in the presence of a divalent cation (usually Mg^2+^, in vivo) to form the corresponding 6‐oxopurine nucleoside monophosphates IMP, GMP, or XMP (**Figure** **1**). In humans, there are two pathways to produce the 6‐oxopurine nucleoside monophosphate: salvage, requiring the use of hypoxanthine‐guanine phosphoribosyltransferase (HGPRT) or de novo synthesis of the purine ring, which is an energy‐consuming process. However, in *Plasmodium falciparum* (*Pf*) and other protozoan parasites, there is no de novo pathway and they rely on the purine salvage pathway to produce their purine nucleoside monophosphates required for DNA/RNA synthesis.^[^ ^1^ ^,^ ^2^ ^]^ *Pf*HGXPRT is also indirectly responsible for the production of adenosine monophosphate (AMP) because the parasite lacks both adenine phosphoribosyltransferase (APRT) and adenylate kinase,^[^ ^3^ ^,^ ^4^ ^]^ though AMP has been reported to be able to be transported into *P. falciparum* from the host cell.^[^ ^4^ ^,^ ^5^ ^]^ Human HGPRT and *Pf*HGXPRT have 44% amino acid sequence identity and, unlike *Pf*HGXPRT,^[^ ^6^ ^,^ ^7^ ^]^ human HGPRT does not utilize xanthine as the substrate.^[^ ^8^ ^]^ Inhibitors which demonstrate selective inhibition between these two enzymes have been designed, but there is, so far, no experimentally determined structural evidence to explain the rationale for this data.^[^ 9, 10, 11, 12, 13, 14 ^]^ The essential role of HGXPRT in the malarial parasite suggests that *Pf*HGXPRT is an excellent target for the development of antimalarial chemotherapeutic agents with high potency and selectivity over human HGPRT. It has been shown recently that prodrugs of inhibitors of human HGPRT are drug leads for triple‐negative breast cancer.^[^ ^15^ ^]^ Thus, the development of inhibitors of either of these enzymes represents an advance in the search for chemotherapeutics against several disease states.

**Figure 2:**
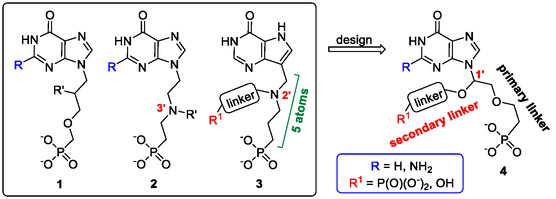
General structures of known HG(X)PRT inhibitors (**1–3**) that were the inspiration for the general scaffold of C1′‐branched ANPs **4** designed here. Structure **1** represents the 9‐phosphono(alkoxy)alkylpurines, **2** and **3** represent the aza‐ANPs with a nitrogen branching atom.

Several classes of HG(X)PRT inhibitors have been developed. These include the transition state analogs known as the immucillin monophosphates,^[^ ^16^ ^]^ the immucillin phosphonates,^[^ ^9^ ^]^ the acyclic nucleoside phosphonates (ANPs),^[^ ^10^ ^]^ and the pyrrolidine^[^ ^11^ ^]^ and prolinol^[^ ^12^ ^]^ phosphonates. Replacement of the phosphate group with phosphonate is a key element in the design of all these inhibitors as the substitution of the labile phosphate ester bond with a phosphonate group confers stability in vivo and this is a critical factor for effective chemotherapeutics. Replacing the rigid (deoxy)ribose moiety with an acyclic moiety increases the overall flexibility of these compounds and this factor may be one reason for selectivity. Based on crystal structures of enzyme‐inhibitor complexes,^[^ ^9^ ^,^ ^10^ ^,^ ^13^ ^,^ ^14^ ^]^ all these compounds fill the purine binding and the 5´‐phosphate binding pockets (Figure 1). The addition of a second phosphonate group designed to occupy the pyrophosphate binding pocket has been found to be effective in increasing the potency and X‐ray crystal structures show that this second group does indeed occupy this pocket in the active site.^[^ ^17^ ^]^ In attempts to improve both potency and selectivity of the ANPs, variations in the chemical composition of the linker that connects the purine base and the phosphonate group have been made. These include the insertion of an oxygen atom at different positions in the acyclic moiety,^[^ ^10^ ^,^ ^11^ ^,^ ^18^ ^,^ ^19^ ^]^ and placing a nitrogen atom at either the third or second position from the purine base (aza‐ANPs)^[^ ^20^ ^]^ (compounds **2** and **3**, respectively, **Figure** **2**). Aliphatic branching linkers, i.e. attachments to the atoms at the second (C2^′^) or third (C3^′^) positions, have also been synthesised and their ability to inhibit HG(X)PRTs determined and analysed. As a result of the above changes, improved *K*_*i*_ values for binding to the HG(X)PRTs have been achieved.^[^ 17, 18, 19 ^,^ ^21^ ^]^ With the improvement in affinity achieved by adding attachments to the second or third atom of the acyclic linker, it has been hypothesized that attachments to the atom closest to the purine ring (C1′) (Figure 2) could also result in potent inhibitors of this class of enzymes. However, the synthesis of such compounds was perceived to be challenging until a series of adenine ANPs were made with a branch in the C1′ position and having a hemiaminal moiety.^[^ 22, 23, 24, 25 ^]^ Here, we have synthesized 12 C1′‐branched ANPs and bisphosphonates of general structure **4** (Figure 2) with varying linker lengths and positions of the oxygen atoms. For clarity, we labeled the linker between the N9 atom and the phosphonate moiety as “primary linker” (compound **4**, Figure 2), while the additional attachment to the C1^′^ atom as “secondary linker” (in the reaction schemes highlighted in red). The prepared compounds have been tested as inhibitors of human HGPRT and *Pf*HGXPRT. Two phosphonodiamidate prodrugs of these compounds with low *K*_*i*_ values were effective in arresting the growth of *P. falciparum* in vivo*.* All of these prodrugs were found to be nontoxic in a number of human cell lines.

## 2 Results and Discussion

### 2.1 Chemistry

**Scheme 1:**
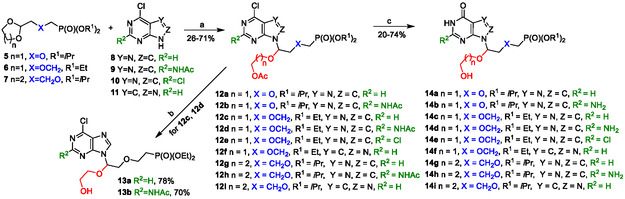
Synthesis of compounds **13a**, **13b**, and **14a–14i** (the secondary linker is highlighted in red). Reagents and conditions: a) Ac_2_O, TMSOTf, or SnCl_4_, MeCN, rt, 15 min; b) Bu_2_SnO, MeOH, MW, 100 °C, 45 min; c) DABCO, K_2_CO_3_, H_2_O, 80 °C, 1–3 h.

Starting acetal‐based phosphonate precursors **5**–**7** (**Scheme** **1**) were prepared according to the published procedures.^[^ ^22^ ^,^ ^25^ ^]^ C1′‐branched ANPs **12a**–**12i** were synthesized using a previously reported multicomponent reaction,^[^ ^23^ ^]^ when acetals **5**–**7** were treated with the corresponding 6‐chloropurine derivatives **8**–**11** and acetic anhydride in the presence of trimethylsilyl trifluorometanesulphonate (TMSOTf) or SnCl_4_ in dry acetonitrile (MeCN). It should be noted that TMSOTf was not always the best catalyst and in some cases (*e.g.*, reactions of acetal **5** with 6‐chloropurine **9** or **10**), using SnCl_4_ offered better yields (optimization experiments not shown). Compounds **12a**–**12i** were obtained in 26–71% yields (Scheme 1).

Deacetylation of compounds **12c** and **12d** gave 6‐chloropurine analogs **13a** and **13b**, respectively, in high yields, while the simultaneous deacetylation and hydrolysis of the 6‐chloro group in **12a**–**12i** afforded 6‐oxopurine derivatives **14a**–**14i** in 20–74% yields (Scheme 1).

**Scheme 2:**
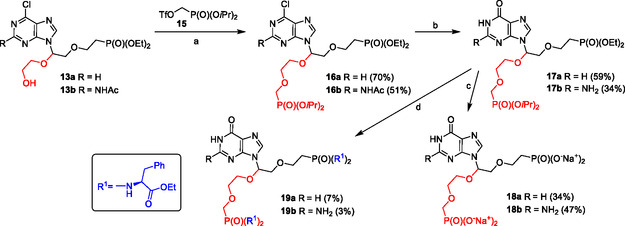
Synthesis of bisphosphonates **18a** and **18b**, and their prodrugs **19a** and **19b** (the secondary linker is highlighted in red). Reagents and conditions: a) *n*‐BuLi, THF/HMPA (3:1), −78 °C to rt; b) DABCO, K_2_CO_3_, H_2_O, 80 °C, 1–3 h; c) 1. TMSBr, pyridine, rt, overnight; 2. H_2_O; 3. 1 m TEAB, H_2_O; 4. DOWEX (Na^+^); d) 1. TMSBr, pyridine, rt, overnight; 2. ethyl ester L‐phenylalanine hydrochloride, Ph_3_P, Aldrithiol‐2, Et_3_N, pyridine, 50 °C, 48 h.

6‐Chloropurine intermediates **13a** and **13b** were used for the synthesis of target bisphosphonates. (Diisopropoxyphosphoryl)methyl trifluoromethanesulfonate **15** and a mixture of THF and hexamethylfosforamid (HMPA) (3:1) were used for the alkylation reaction (HMPA was added to improve the solubility of the starting materials as the conversion in pure THF was low; optimization not shown). Thus, the treatment of compounds **13a** and **13b** with *n*‐BuLi and **15** afforded bisphosphonates **16a** and **16b** in good (over 50%) yields (**Scheme** **2**).

Because of the low stability of hemiaminal derivatives in acidic conditions, hydrolysis using 1,4‐diazabicyclo [2.2.2]octane (DABCO) and K_2_CO_3_^[^ ^18^ ^]^ was employed for the conversion of 6‐chloropurine derivatives **16a** and **16b** into 6‐oxopurine analogs **17a** and **17b**, respectively (Scheme 2). Under these reaction conditions, a simultaneous removal of the acetyl group from the NHAc moiety of **16b** was achieved.

Compounds **17a** and **17b** were then converted into free bisphosphonates **18a** and **18b** (in the form of sodium salt) by the standard procedure,^[^ ^20^ ^]^ using bromotrimethylsilane (TMSBr) in pyridine, followed by the hydrolytic workup (Scheme 2). Finally, the standard one‐pot procedure,^[^ ^20^ ^]^ using a *trans*‐silylation and the treatment with a mixture of ethyl L‐phenylalanine hydrochloride, triphenylphosphine, and Aldrithiol‐2 in pyridine/Et_3_N was used for the synthesis of desired phosphonodiamidate prodrugs **19a** and **19b** (Scheme 2) from compounds **17a** and **17b**.

**Scheme 3:**
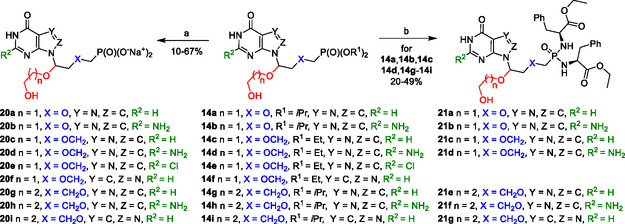
Synthesis of phosphonates **20a**–**20i** and prodrugs **21a**–**21g** (the secondary linker is highlighted in red). Reagents and conditions: a) 1. TMSBr, pyridine, rt, overnight; 2. H_2_O; 3. 1 m TEAB, H_2_O; 4. DOWEX (50WX8 Na^+^); b) 1. TMSBr, pyridine, rt, overnight; 2. ethyl ester L‐phenylalanine hydrochloride, Ph_3_P, Aldrithiol−2, Et_3_N, pyridine, 50 °C, 48 h.

Similarly, C1′‐branched ANPs **14a**–**14i** were converted to their free phosphonates **20a**–**20i** (as sodium salts) and selected derivatives **14** into their phosphonodiamidate prodrugs **21a**–**21g** (**Scheme** **3**).

**Scheme 4:**
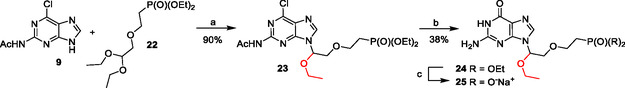
Synthesis of phosphonate **25** (the secondary linker is highlighted in red). Reagents and conditions : a) Ac_2_O, TMSOTf, MeCN, rt, 15 min; b) DABCO, K_2_CO_3_, H_2_O, 80 °C, 2 h; c) 1. TMSBr, pyridine, rt, overnight; 2. H_2_O; 3. 1 M TEAB, H_2_O; 4. DOWEX (50WX 8 Na^+^).

To further evaluate the influence of the secondary linker at C1^′^ position on the enzyme inhibition, compound **25** (**Scheme** **4**) bearing the simple ethoxy moiety, was designed. Compound **25** was prepared by a condensation of 6‐chloropurine derivative **9** with diethyl acetal‐based phosphonate precursor **22**,^[^ ^25^ ^,^ ^20^ ^]^ to give **23**, followed by the simultaneous hydrolysis of the 6‐chloro group and acetyl moiety removal, and by conversion to the phosphonate sodium salt.

### 2.2 Enzyme Inhibition Studies

The *K*_*i*_ values for the C1′‐branched ANPs were determined both against human HGPRT and *Pf*HGXPRT (**Table** **1**).

**Table 1:** The *K*_i_ (µM) values of C1′‐branched ANPs for human HGPRT and *Pf*HGXPRT.

Comp	B (base)a)	Secondary linker at C1′	human HGPRTc)	*Pf*HGXPRTc)
**18a**	Hx	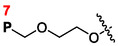	> 60	5 ± 1
**18b**	G	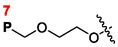	0.7 ± 0.4	1 ± 0.2
**20a** b)	Hx	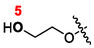	160	ND
**20b** b)	G	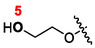	30 ± 5	5 ± 1
**20c**	Hx	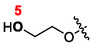	> 20	10 ± 5
**20d**	G	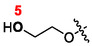	2.5 ± 0.4	0.4 ± 0.1
**20e**	2‐ClHx	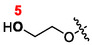	> 60	50 ± 10
**20f**	9‐deaza‐8‐azaHx	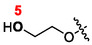	> 60	> 60
**20g**	Hx	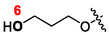	> 60	> 60
**20h**	G	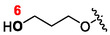	10 ± 3	> 60
**20i**	9‐deaza‐8‐azaHx	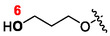	> 60	> 60
**25**	G	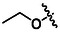	3.7 ± 0.9	3.0 ± 0.5

a) B (base): Hx, hypoxanthine; G, guanine; 2‐ClHx, 2‐chlorohypoxanthine; 9‐deaza‐8‐azaHx, 9‐deaza‐8‐azahypoxanthine;

b) For **20a** and **20b**, the primary linker between N9 atom and the phosphonate group was shortened by one carbon to a 4‐atom‐linker;

c) Data represent the mean ± SD of three independent experiments. (Note: a red number next to the atom in the secondary linker states the distance of the atom from N9 atom of the nucleobase).

All the C1′‐branched ANPs have the 5‐atom‐long primary linker connecting the purine base to a phosphonate group, except for **20a** and **20b** with 4‐atom‐linkers. For **20a** and **20b**, this linker was shortened by a single carbon atom (Table 1, footnote b)). The removal of the carbon atom results in a significant increase in the *K*_*i*_ values, when comparing the guanine derivatives, from 2.5 to 30 μM for human HGPRT and from 0.4 to 5 μM for *Pf*HGXPRT (*cf* **20d** with **20b**) (Table 1). It is concluded that this increase is due to the differences in the length of the primary linker so that the single phosphonate of **20b** cannot be pushed as far into the 5′‐phosphate binding pocket as would be the case for **20d**. This decreases the interactions of the phosphonyl oxygens with main chain and/or side chain atoms of the amino acids in the flexible loop which surrounds the natural 5′‐phosphate group. The addition of the secondary linker to C1′ position cannot efficiently counter this effect for **20a** and **20b**.

**Figure 3:**
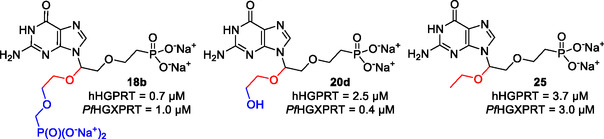
A comparison of chemical structures of the three most active C1^′^‐branched inhibitors of human HGPRT and *Pf*HGXPRT (the secondary C1′‐linkers highlighted in colors).

The compounds that contain hypoxanthine as the base, and with the same 5‐atom primary linker as their guanine analogs in **Figure** **3**, exhibited poor activity against human HGPRT (**18a**, **20c** and **20g** with 60, > 20, and > 60 µM, respectively). Compounds **18a** and **20c** did, however, show good inhibition of *Pf*HGXPRT with *K*_*i*_ values of 5 and 10 µM, respectively (Table 1). The exception for the hypoxanthine‐containing compounds is **20g** (> 60 µM), but this is because the secondary 1‐atom‐longer linker at C1′ atom of this compound hinders binding of the inhibitor in the active site. The C1′‐branched ANP compounds containing unnatural nucleobases (**20e**, **20f**, and **20i**) are also very weak inhibitors of both these enzymes (Table 1). In contrast, the corresponding compounds that contain guanine, namely **18b** and **20d**, are reasonable inhibitors of human HGPRT, 0.7 and 2.5 µM, respectively. For *Pf*HGXPRT, these values are 1 and 0.4 µM, with **20d** exhibiting selectivity for the parasite enzyme. Thus, the inhibitors with guanine as the nucleobase have the highest affinity for both human HGPRT and *Pf*HGXPRT, emphasizing the importance of the exocyclic amino group on the purine base for effective inhibition of the C1′‐branched ANPs.

Compound **18b** (Figure 3, Table 1) with a second phosphonate group at the end of the secondary linker (bisphosphonate) is the most potent inhibitor of human HGPRT and the second most potent for *Pf*HGXPRT, with *K*_*i*_ values of 0.7 and 1 µM, respectively. The similarity in the inhibition constants suggests that this compound may form similar interactions with main chain or side chain atoms in the active sites of both enzymes. The addition of the second phosphonate group was very effective in lowering the *K*_*i*_ value for the human enzyme in comparison with those C1′‐branched ANPs that do not contain this second phosphonate group (Table 1).

Compound **20d** (Figure 3, Table 1) is the most potent inhibitor of *Pf*HGXPRT with a *K*_*i*_ of 0.4 µM. This is slightly lower than for **18b,** which contains a second phosphonate group. The value for the human enzyme is higher (2.5 µM). This selectivity for *Pf*HGXPRT could be attributed to a different mode of binding to the parasite enzyme compared with the human enzyme.

If the secondary linker attached to the C1^′^ atom, terminated with a hydroxyl group, is lengthened by one carbon atom (*cf*. **20d** with **20 h**, Table 1), the *K*_*i*_ values for **20 h** increase significantly to 10 µM (human HGPRT) and > 60 µM (*Pf*HGXPRT) with guanine as the base. Similar trend can be seen for the hypoxanthine analogs **20c** and **20g** (Table 1), although the oxygen atom position in the primary linkers of the same length can also have a certain effect on the potency of the compounds. It is concluded that the longer secondary linker with the hydroxyl group makes it more difficult for these inhibitors to fit into the active site, particularly for *Pf*HGXPRT. The guanine compound **25** (Figure 3), lacking the terminal phosphonomethoxy or hydroxyl group (compared to **18b** and **20d**, respectively), is a good inhibitor of both enzymes with *K*_*i*_ values of 3.7 and 3.0 µM, for human HGPRT and *Pf*HGXPRT, respectively. This suggests that, for this compound, the mode of binding is similar in the active site of both enzymes and that the second group attached to the C1′ atom does not contribute to affinity (*cf* **18b**).

Thus, **18b** and **20d** are good inhibitors of both *Pf*HGXPRT and human HGPRT with **18b** being the most potent, though unselective, inhibitor of the human enzyme, and **20d** being the most potent for *Pf*HGXPRT and is selective (Table 1 and Figure 3). To advance a structural explanation for these differences, docking studies were conducted with compounds **18b** and **20d** using the known X‐ray crystal structures of the human HGPRT and *Pf*HGXPRT in complex with inhibitors as the templates.

### 2.3 Docking Studies

**Figure 4:**
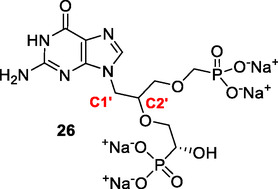
The chemical structure of ligand **26** used as the template for the docking studies with human HGPRT (PDB code: 7SAN).^[^ ^29^ ^]^

In the absence of experimental crystal structures, docking studies are a valuable guide in understanding how a novel compound may bind in the active site of an enzyme. For human HGPRT, there are 19 structures available in the protein data bank which can be used as templates for docking analysis. These include structures of the enzyme in the absence of ligands,^[^ ^26^ ^]^ in complex with ANP inhibitors,^[^ ^10^ ^,^ ^20^ ^]^ in complex with inhibitors containing a five membered ring in the linker connecting the purine base to the phosphonate group,^[^ ^12^ ^,^ ^27^ ^]^ in complex with GMP,^[^ ^28^ ^]^ in complex with the transition state analog, and in complex with inhibitors which mimic the chemical structure of the transition state.^[^ ^9^ ^,^ ^13^ ^]^ Collectively, these structures emphasize a high degree of conformational variability which occurs in the active site when different ligands bind. It was predicted that the crystal structure of human HGPRT is likely to have the most similar protein structure and, therefore, the best optimized for the C1^′^‐branched compounds to dock was that in complex with (4*S*,7*S*)‐7‐hydroxy‐4‐((guanin‐9‐yl)methyl)−2,5‐dioxaheptan‐1,7‐diphosphonate (**26**, **Figure** **4**) (PDB code: 7SAN).^[^ ^29^ ^]^ This was because this inhibitor has 5‐atom‐linkers connecting the purine base to a phosphonate group (Figure 4), similarly to the C1′‐branched compounds made herein. The difference is that the secondary linker is attached to the C2′ atom in the acyclic linker instead of C1′.

**Figure 5:**
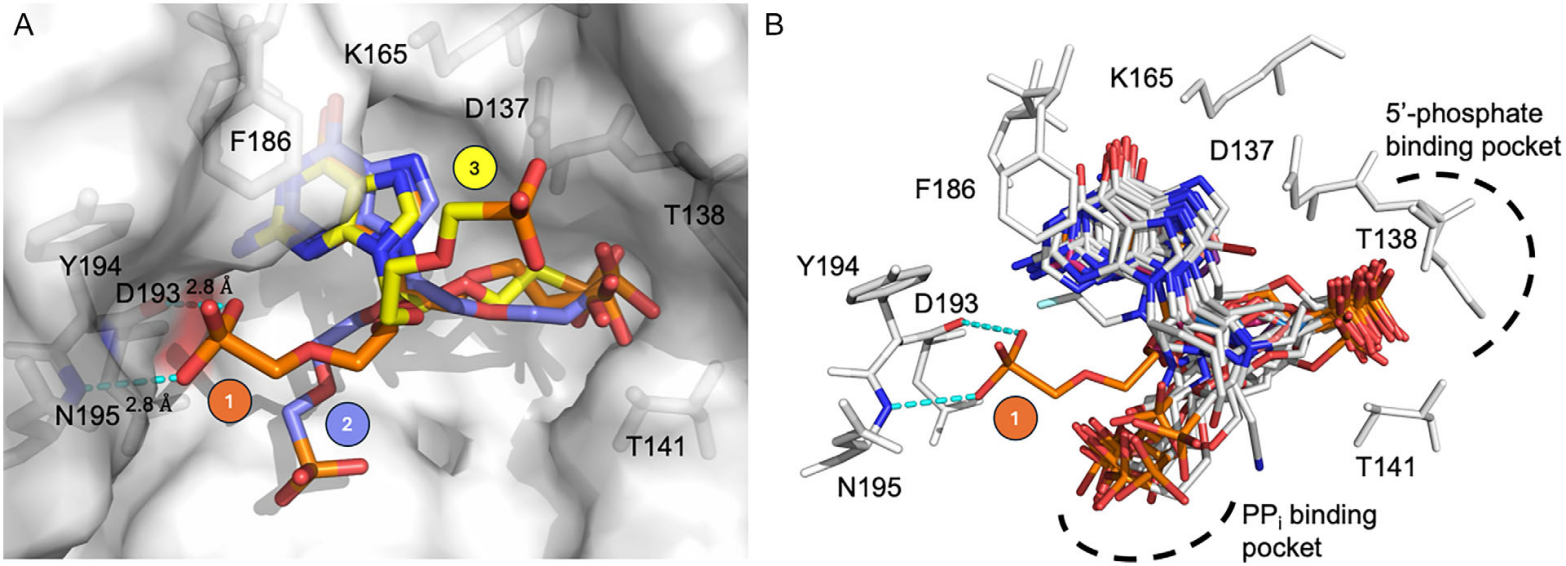
The top three docking poses (labeled 1, 2, and 3) for compound **18b** into the active site of human HGPRT. A) The transparent Connolly surface of the human HGPRT and **26** complex (PDB code: 7SAN). The three highest‐scoring binding poses of **18b** are shown as stick models and labeled with spheres numbered 1, 2, and 3. The ChemPLP scores for these poses are similar: 117.87, 115.02, and 114.43. B) Superimposition of the highest docking pose of **18b** with structures of 19 inhibitors from complexes with human HGPRT overlaid. The overlaid structures have PDB codes of 1BZY, 1DN6, 3GEP, 3GGC, 3GGJ, 4JIQ, 4KN6, 4RAB, 4RAC, 4 RAD, 4RAN, 4RAO, 4RAQ, 5BRN, 5BSK, 5W8V, 7SAN, 8TPV, and 8TPY.

The attachment at the C2′ position in compound **26** is one atom shorter (5 atoms between N9 and the phosphonate group) than that of **18b** (Table 1, Figure 3), though it also has a phosphonate group at the terminal end of the secondary linker. The docking studies show that the three highest‐ranking poses, when **18b** is docked into the human HGPRT structure where **26** was bound, all have the purine base in the expected binding pocket and the phosphonate group on the primary linker reaching into the 5′‐phosphate binding pocket (**Figure** **5**). However, the pose for the C1′ attachment is highly variable even among the highest‐scoring results. The only pose, pose number 1, where the phosphonate group of the C1′ attachment is in contact with a part of the surface of human HGPRT, is the one with highest ranking score (Figure 5A) and the phosphonate oxygen atoms are observed to make two hydrogen bonds, one with the backbone amide of N195 and the other with the carbonyl of D193. This potential binding site for a phosphate/phosphonate group has not been observed as a binding location for any inhibitor of HG(X)PRTs. Figure 5B shows the superimposition of all the previously determined crystal structures of human HGPRT inhibitor complexes with the predicted number 1 pose of **18b**. This demonstrates the possible differences in the binding mode of **18b** compared with other known inhibitors. Thus, positioning the secondary linker to the C1′ atom of the primary linker provides new ideas for further rational structure‐based design.

**Figure 6:**
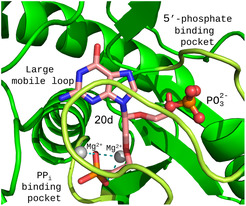
Docking of **20d** to the active site of *Pf*HGXPRT. Docking was performed using the protein coordinates and the Mg^2+^ ion locations from the *Pf*HGXPRT structure with PDB code 1CJB. The coordinates for phosphate were superimposed based on its position in the *Pf*HGXPRT structure with PDB code 3OZF. It is therefore possible for both **20d** and phosphate to be bound in the active site at the same time. The pyrophosphate binding pocket, 5′‐phosphate binding pocket, and the large mobile loop that closes over the active site and is visible in the structures of 1CJB and 3OZF, are shown in light green and labeled accordingly.

There are only four crystal structures of *Pf*HGXPRT which have been determined in complex with different ligands: i) the transition state analog, (1*S*)‐1‐(9‐deazahypoxanthin‐9‐yl)−1,4‐dideoxy‐1,4‐imino‐D‐ribitol 5‐phosphate (immucillinHP), pyrophosphate, and Mg^2+^ (PDB code 1CJB);^[^ ^14^ ^]^ ii) the acyclic immucillin phosphate, (3*S*)‐4‐hydroxy‐3‐[[(4‐oxo‐4,5‐dihydro‐3*H*‐pyrrolo[3,2‐d]pyrimidin‐7yl)methyl]amino]butyl]phosphonate (PDB code 3OZG);^[^ ^9^ ^]^ iii) in complex with hypoxanthine, phosphate, pyrophosphate, and magnesium (PDB code 3OZF);^[^ ^9^ ^]^ and iv) in complex with the acyclic immucillin phosphate, [(3*S*)‐4‐hydroxy‐3‐[([2‐amino‐4‐hydroxy‐5*H*‐pyrrolo[3,2‐d]pyrimidin‐7‐yl]methyl)amino]butyl] phosphonate, pyrophosphate, and Mg^2+^ (PDB code 7TUX). In all these structures, a large mobile loop consisting of ≈20 amino acids is closed over the active site and it has been suggested that this closure occurs to sequester the active site from exposure to solvent.^[^ ^17^ ^]^ Closure of this loop also results in extra interactions at the active site, which could contribute to lowering the *K*_*i*_ values. Based on the structures of human HGPRT in complex with inhibitors which are not transition state analogs (Figure 5), this loop movement is unlikely to occur when the C1^′^‐branched inhibitors bind to *Pf*HGXPRT. Therefore, the available structures of *Pf*HGXPRT may not provide the ideal protein template model for predicting the binding of compounds such as **18b** or **20d**. Nonetheless, we performed a docking study using the structure of *Pf*HGXPRT (PDB code 1CJB as a protein template)^[^ ^14^ ^]^ and docked **20d**. The highestranked solution locates the phosphonate group of **20d** into the pyrophosphate binding pocket, leaving the 5′‐phosphate binding pocket empty (**Figure** **6**). The reason for this is that the secondary linker at the C1′ position in **20d** does not have a terminal phosphonate group, so instead it reaches down to the pyrophosphate binding pocket. In all the reported X‐ray crystal structures of the HG(X)PRTs, a critical criterion for a ligand to bind appears to be that the 5′‐phosphate binding pocket is occupied. The *Pf*HGXPRT enzyme used for the inhibition studies of the C1′‐branched ANPs is purified and stored in phosphate buffer. Thus, it is likely that a phosphate ion already occupies the 5′‐phosphate site. Thus, the active site of *Pf*HGXPRT is optimally adjusted to allow the linker and phosphonate to bind in an alternative conformation where the phosphonate binds to the pyrophosphate binding pocket. This would account for the fact that **20d** has the lowest *K*_*i*_ value for the *Plasmodium* enzyme in this series of inhibitors and is lower than for the human enzyme, which does not have a phosphate ion present. It can be concluded that **18b**, which has two phosphonate groups, may bind to *Pf*HGXPRT in a similar manner as to the human enzyme, accounting for the fact that their *K*_*i*_ values are similar (Table 1 and Figure 3).

These docking poses demonstrate the differences that can occur when the C1′‐branched compounds bind to either of these two enzymes, providing, in part, an explanation for similarities and differences in their inhibition constants, especially for **18b** and **20d**.

The C1^′^‐branched compounds synthesized here are not as potent as the transition state analogs (AIPs) and ANPs containing two phosphonate groups (bisphosphonates), all of which exhibit a range of *K*_*i*_ values and selectivity toward the human and *Pf* enzymes.^[^ ^9^ ^,^ ^17^ ^,^ ^18^ ^,^ ^20^ ^,^ ^29^ ^]^ Nonetheless, some of the novel compounds developed here do show strong affinity for human HGPRT and *Pf*HGXPRT, with *K*_*i*_ values lower than for the *K*_*i*_ values of the nucleotide products GMP and IMP, 10.0 ± 2 µM and 3.6 ± 1 µM for *Pf*HGXPRT and 5.8 ± 0.2 µM and 5.4 ± 1.2 µM for human HGPRT.^[^ ^10^ ^]^ It is not known why the C1′‐branched ANPs with hypoxanthine as the nucleobase do not bind or bind only weakly to human HGPRT, but, for this class of compounds, it is apparent they require the guidance afforded by the 2‐amino group attached to the purine ring (i.e.*,* guanine) to enable efficient binding.

### 2.4 Activity of Prodrugs against *Plasmodium falciparum* Cell Lines

The C1′‐branched ANPs possess negative charges on their phosphonate group(s), making it difficult for them to cross cell membranes. To facilitate transport across the red blood cell membrane and then into the parasite itself, phosphonodiamidate prodrugs were synthesized (Schemes 2 and 3). Once inside the cell, the masking groups must be removed by inherent enzymes, resulting in the release of the nontoxic amino acid (phenylalanine), as well as the active parent compound. For these assays, two *P. falciparum* lines were chosen, D6 and W2, both of which are laboratory‐adapted cell lines. The D6 cell line is highly susceptible to most antimalarial drugs, including chloroquine, while the W2 cell line is resistant to many antimalarial drugs, including chloroquine. The lowest IC_50_ values for these prodrugs are for **19a**, the prodrug of bisphosphonate **18a,** and these values are 2 μM in the D6 cell line and 3 μM in the W2 cell line while, for **19b**, the prodrug of bisphosphonate **18b**, these values are 4.3 and 8.8 μM, respectively (**Table** **2**). These two prodrugs are those whose parent compound has the lowest *K*_*i*_ values for *Pf*HGXPRT (Table 1). The potency of **21a**–**21g** (Table 2), the prodrugs of ANPs, which have much higher *K*_*i*_ values (> 60 μM), was much lower with IC_50_ values > 10 μM. The percentage of the prodrug that enters the parasite and is then hydrolyzed to the active component is unknown at the moment and is the subject of ongoing studies. The IC_50_ values do show that a significant proportion of the prodrug is taken up by the cells,‐ and the masking groups have been removed to produce sufficient concentrations of the inhibitor to be effective in vitro.

**Table 2:** The IC_50_ values for the phosphonodiamidate prodrugs **19a**, **19b**, and **21a**–**21g** in *Pf* infected erythrocytes.

Prodrug	Parent comp.	IC_50_ [µM]a)	IC_50_ [µM]a)
		D6b)	W2c)
**19a**	**18a**	2.1 ± 0.2	3.4 ± 1.1
**19b**	**18b**	4.3 ± 0.7	8.8 ± 2.6
**21a**	**20a**	34 ± 6	71 ± 1
**21b**	**20b**	84 ± 10	79 ± 8
**21c**	**20c**	41 ± 5	14 ± 2
**21d**	**20d**	64 ± 6	39 ± 5
**21e**	**20g**	14 ± 2	41 ± 5
**21f**	**20h**	13 ± 2	26 ± 14
**21g**	**20i**	11 ± 1	15 ± 2

a) Data represent the mean ± SD of three independent experiments;

b) D6 is a strain of *P. falciparum* sensitive to chloroquine;

c) W2 is *P. falciparum* strain resistant to chloroquine.

Prodrugs **19a** and **21a –21g** were also assessed for their cytotoxicity in five different human cell lines, namely HL‐60, CCRF‐CEM, HeLa, HepG2, and NHDF (Table S1, Supporting Information). At a concentration of 10 μM, none of the compounds exhibited any reduction in growth in any of the cell lines, suggesting these compounds are nontoxic under in vivo conditions.

## 3 Conclusions

These studies with ANPs bearing different secondary linkers attached to the C1′‐position of the primary acyclic linker complement those on ANPs, where attachments were made to other positions in the primary acyclic linker. Phosphonodiamidate prodrugs of those inhibitors with low *K*_*i*_ values arrest the growth of *Pf* in cell culture and they are nontoxic in the human cell lines tested. This data reinforces the proposition that inhibitors of this enzyme are potential drug leads against parasitic infections. The docking studies reveal new potential binding sites for the phosphonyl groups, which can be exploited for chemical alterations to the primary ANP scaffold to further enhance potency and selectivity of these compounds.

## 4 Experimental Section

4.1

4.1.1

##### General Remarks

**Figure 7:**
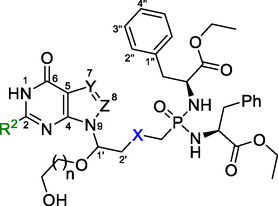
The atom numbering used for NMR signal assignment.

Unless otherwise stated, solvents were evaporated at 40 °C (2 kPa) and prepared compounds were dried at 30 °C (2 kPa). Reaction flasks were heated in aluminum heating blocks. Tetrahydrofuran, dioxane, and acetonitrile were dried by activated neutral alumina (Drysphere). Dimethylformamide was dried by activated molecular sieves (3 Å). Other dry solvents were purchased from commercial suppliers. Analytical thin layer chromatography (TLC) was performed on silica gel precoated aluminum plates with the fluorescent indicator Merck 60 F254 (Sigma–Aldrich). Flash column chromatography was carried out using Teledyne ISCO CombiFlash Rf200 with a dual absorbance detector (Teledyne ISCO, Lincoln, NE, USA). HRMS spectra (ESI^+^ or EI^+^) were recorded on LTQ Orbitrap XL spectrometer (Thermo Fisher Scientific, Waltham, MA, USA) with the electrospray ionization (ESI) or electron ionization (EI) ionization method. NMR spectra were recorded on a Bruker (Rheinstetten, Germany) Avance III 500 MHz spectrometer (^1^H at 500.0 MHz, ^13^C at 125.7 MHz) and referenced to the residual solvent signal (DMSO at 2.50 and 39.70 ppm in ^1^H and ^13^C, respectively; CDCl_3_ at 7.26 and 77.00 ppm in ^1^H and ^13^C, respectively) or to an internal standard for measurement in D_2_O (dioxane at 3.75 and 67.19 ppm in ^1^H and ^13^C, respectively, *tert*‐butyl alcohol at 1.24 and 30.29 ppm in ^1^H and ^13^C, respectively). The basic atom numbering used for proton and carbon signal assignment is shown in **Figure** **7**.

Purity of compounds was measured on Waters UPLC‐MS system consisting of Waters UPLC H‐Class Core System (column Waters ACQUITY UPLC BEH C18 1.7 μm, 2.1 fcalc 100 mm), Waters ACQUITY UPLC PDA detector, and Mass spectrometer Waters SQD2. Universal LC method was used (eluent H_2_O/MeCN, gradient 0–100%, run length 7 min) and MS method (ESI^+^ and/or ESI^−^, cone voltage = 30 V, mass detector range 100–1000 Da). Purity of the final compounds was > 95%. Starting compounds and reagents were purchased from commercial suppliers and used without purification. Starting acetal‐based phosphonate precursors **5**–**7** and **22** were prepared according to the published procedures.^[^ ^22^ ^,^ ^25^ ^]^

##### General Methods: General Method A: Preparation of Purine Hemiaminal Phosphonates from acetal. Phosphonates

Trimethylsilyl trifluoromethanesulfonate (3.0 eq.) or SnCl_4_ (3.0 eq.) was added to a mixture of the corresponding purine nucleobase (1.0 eq.), acetal‐based phosphonate precursors (1.0 eq.), and acetic anhydride (1.0 or 1.5 eq.) in dry acetonitrile and the resulting mixture was stirred for 15 min at room temperature. The mixture was then poured into water and subsequently extracted with chloroform (3 × 15 mL). The combined organic layers were washed with brine, dried over Na_2_SO_4_, and evaporated. The crude product was purified by flash chromatography (silica gel, chloroform to 20% methanol or hexane to 30% ethyl acetate) to obtain the pure product.

##### General Methods: General Method B: Hydrolysis of 6‐Chloropurines to 6‐Oxopurines

A mixture of the corresponding 6‐chloropurine hemiaminal phosphonate (1.0 eq.), DABCO (1.1 eq.), and K_2_CO_3_ (2.1 eq.) in water was heated at 50 °C or 80 °C until the reaction was completed (monitored by UPLC/MS). Additional K_2_CO_3_ and DABCO were added to the mixture when needed. Water was removed in vacuo, the residue dissolved in chloroform, and subsequently filtered using a short plug of silica gel, followed by washing with an excess of chloroform. Solvent was removed in vacuo and the crude product was adsorbed on silica gel and purified by flash chromatography (silica gel, chloroform to 30% methanol for 20 column volumes) to obtain pure 6‐oxopurine derivative.

##### General Methods: General Method C: Preparation of Phosphonate Sodium Salts

Excess trimethylsilyl bromide (TMSBr) was added to a solution of the corresponding dialkyl phosphonate (or tetraalkyl bisphosphonate) in dry pyridine, and the mixture was stirred for 12 h at room temperature. Solvent was removed in vacuo and the residue was dissolved in an excess of 1 M solution of triethylammonium bicarbonate (TEAB**)** and evaporated to dryness. The crude TEAB‐salt was purified by flash chromatography (C18 silica gel, 0.125 M aqueous TEAB to 40% methanol for 20 column volumes). The fractions with pure TEAB‐salt were combined and evaporated in vacuo. The residue was dissolved in a small amount of water and percolated through DOWEX (50WX8 Na^+^, 5 g), evaporated, and lyophilized from water to obtain the pure product.

##### General Methods: General Method D: Preparation of Phosphonodiamidate Prodrugs

Excess TMSBr was added to a solution of the corresponding dialkyl phosphonate (or tetraalkyl bisphosphonate) in dry pyridine under an argon atmosphere, and the mixture was stirred overnight at room temperature. Solvent was removed in vacuo and the flask with the residue was purged with argon and l‐alanine ethyl ester hydrochloride, dry trimethylamine, and dry pyridine were added. The reaction mixture was stirred at 55 °C for ≈1 min (to obtain a homogenous solution) and a solution of Aldrithiol−2 and triphenylphosphine in dry pyridine was added. The mixture was heated at 50 °C for 48 h. After cooling, the volatiles were removed in vacuo and the solid residue was purified by flash chromatography on silica gel (0–100% methanol in a hexane/EtOAc (6:4) mixture). The crude product was purified by flash chromatography (C18 reverse phase silica gel using 0–100% H_2_O to MeOH gradient for 20 column volumes). The pure product was lyophilized from 1,4‐dioxane to give the pure product.

##### Chemical Synthesis: Diisopropyl ((2‐(2‐acetoxyethoxy)‐2‐(6‐chloro‐9*H*‐purin‐9‐yl)ethoxy)methyl)phosphonate (12a)

Prepared according to general method A from **5** (840 mg, 3.0 mmol), **8** (460 mg, 3.0 mmol), acetic anhydride (0.28 mL, 300 mg, 3.0 mmol), and TMSOTf (1.6 mL, 1.2 g, 8.9 mmol) in dry MeCN (12 mL) to give **12a** (1.0 g, 71%) as a yellow viscous oil. ^1^H NMR (500 MHz, CDCl_3_): *δ* = 8.68 (s, 1H, H‐2), 8.37 (s, 1H, H‐8), 6.01 (dd, *J*(1′‐2′a) = 5.6 Hz, *J*(1′‐2′b) = 4.4 Hz, 1H, H‐1′), 4.55–4.63 (m, 2H, CH*iPr*), 4.16 (ddd, *J*(gem) = 12.4 Hz, *J*(CH_2_—CH_2_) = 6.9 Hz, *J*(CH_2_—CH_2_) = 2.9 Hz, 1H, AcO—CH_2_a), 4.13 (ddd, *J*(gem) = 10.7 Hz, *J*(2′a‐1′) = 5.6 Hz, *J*(2′a‐P) = 0.6 Hz, 1H, H‐2′a), 4.06 (ddd, *J*(gem) = 12.4 Hz, *J*(CH_2—_CH_2_) = 5.8 Hz, *J*(CH_2_—CH_2_) = 3.0 Hz, 1H, AcO—CH_2_b), 4.01 (ddd, *J*(gem) = 10.7 Hz, *J*(2′b‐1′) = 4.4 Hz, *J*(2′b‐P) = 0.6 Hz, 1H, H‐2′b), 3.75 (s, 1H, CH_2_a‐P), 3.75 (m, 1H, 1′‐O—CH_2_b), 3.74 (s, 1H, CH_2_b‐P), 3.53 (ddd, *J*(gem) = 11.4 Hz, *J*(CH_2_b—CH_2_a) = 6.9 Hz, *J*(CH_2_b—CH_2_b) = 3.0 Hz, 1H, 1′‐O—CH_2_b), 1.96 (s, 3H, CH_3_—COO), 1.22, 120, 1.16, and 1.14 (4 × d, *J*(CH_3_—CH) = 6.2 Hz, 4 × 3H, CH_3_ *iPr*). ^13^C NMR (125 MHz, CDCl_3_): *δ* = 170.63 (COO), 152.20 (C‐2), 151.98 (C‐4), 151.18 (C‐6), 143.90 (C‐8), 131.43 (C‐5), 82.98 (C‐1′), 72.59 (d, *J*(C—P) = 8.7 Hz, C‐2′), 71.23, and 71.29 (2 × d, *J*(C—P) = 6.7 Hz, CH*iPr*), 67.76 (1′‐O—CH_2_), 66.44 (d, *J*(C—P) = 167.6 Hz, CH_2_—P), 62.56 (AcO—CH_2_), 23.81–24.03 (m, CH_3_ *iPr*), 20.72 (CH_3_—COO). HRMS (ESI^+^): *m/z* [*M* + H]^+^ calcd. for C_18_H_28_O_7_N_4_ClNaP = 501.12764, found: 501.12769.

##### Chemical Synthesis: Diisopropyl ((2‐(2‐acetoxyethoxy)‐2‐(2‐acetamido‐6‐chloro‐9*H*‐purin‐9‐yl)ethoxy)methyl)phosphonate (12b)

Prepared according to general method A from **5** (1.3 g, 4.7 mmol), **9** (1.0 g, 4.7 mmol), acetic anhydride (0.67 mL, 720 mg, 7.1 mmol), and SnCl_4_ (1.7 mL, 3.7 g, 14 mmol) in dry MeCN (10 mL) to give **12b** (1.1 g, 41%) as a yellow viscous oil. ^1^H NMR (500 MHz, DMSO‐*d*_*6*_): *δ* = 10.87 (s, 1H, NH), 8.66 (s, 1H, H‐8), 5.95 (dd, *J*(1′‐2′a) = 7.1 Hz, *J*(1′‐2′b) = 4.9 Hz, 1H, H‐1′), 4.39–4.48 (m, 2H, CH*iPr*), 4.23 (dd, *J*(gem) = 10.7 Hz, *J*(2′a‐1′) = 7.2 Hz, 1H, H‐2′a), 4.11 (ddd, *J*(gem) = 12.4 Hz, *J*(CH_2_—CH_2_) = 6.7 and 2.8 Hz, 1H, AcO—CH_2_a), 4.06 (dd, *J*(gem) = 10.7 Hz, *J*(2′b‐1′) = 4.9 Hz, 1H, H‐2′b), 4.00 (ddd, *J*(gem) = 12.4 Hz, *J*(CH_2_—CH_2_) = 6.2 and 2.9 Hz, 1H, AcO—CH_2_b), 3.77–3.88 (m, 3H, CH_2_—P, 1′‐O—CH_2_a), 3.60 (ddd, *J*(gem) = 11.7 Hz, *J*(CH_2_—CH_2_) = 6.7 and 2.9 Hz, 1H, 1′‐O—CH_2_b), 2.21 (s, 3H, CH_3_—CON), 1.91 (s, 3H, CH_3_—COO), 1.13, (2 × d, *J*(CH_3—_CH) = 6.2 Hz, 2 × 3H, CH_3_ *iPr*), 1.06 (2 × d, *J*(CH_3—_CH) = 6.2 Hz, 2 × 3H, CH_3_ *iPr*). ^13^C NMR (125 MHz, DMSO‐*d*_*6*_): *δ* = 170.42 (COO), 169.13 (CON), 153.18 (C‐4), 152.45 (C‐2), 149.43 (C‐6), 145.09 (C‐8), 127.50 (C‐5), 82.77 (C‐1′), 71.27 (d, *J*(C—P) = 12.5 Hz, C‐2′), 70.40–70.48 (m, CH*iPr*), 67.27 (1′‐O—CH_2_), 65.31 (d, *J*(C—P) = 164.5 Hz, CH_2_—P), 62.84 (AcO—CH_2_), 24.87 (CH_3_—CON), 23.88 (d, *J*(C—P) = 3.8 Hz, CH_3_ *iPr*), 23.68 (d, *J*(C—P) = 4.6 Hz, CH_3_ *iPr*), 20.69 (CH_3_—COO). HRMS (ESI^+^): *m/z* [*M* + H]^+^ calcd. for C_20_H_32_O_8_N_5_ClP = 536.16715, found: 536.16735.

##### Chemical Synthesis: Diethyl (2‐(2‐(2‐acetoxyethoxy)‐2‐(6‐chloro‐9*H*‐purin‐9‐yl)ethoxy)ethyl)phosphonate (12c)

Prepared according to general method A from **6** (500 mg, 1.9 mmol), **8** (300 mg, 1.9 mmol), acetic anhydride (0.2mL, 190 mg, 1.9 mmol), and TMSOTf (1.0 mL, 1.3 g, 5.8 mmol) in dry MeCN (10 mL) to give **12c** (550 mg, 64%) as a yellowish viscous oil. ^1^H NMR (500 MHz, DMSO‐*d*_*6*_): *δ* = 8.91 (s, 1H, H‐8), 8.82 (s, 1H, H‐2), 6.06 (dd, *J*(1′‐2′) = 6.7 and 4.9 Hz, H‐1′), 4.06–4.12 (m, 2H, H‐2′a, AcO—CH_2_a), 3.96–4.00 (m, 2H, H‐2′b, AcO—CH_2_b), 3.84–3.90 (m, 4H, CH_2_—CH_3_), 3.81 (ddd, *J*(gem) = 11.6 Hz, *J*(CH_2—_CH_2_) = 6.0 and 2.8 Hz, 1H, 1′‐O—CH_2_a), 3.58–3.69 (m, 2H, CH_2_—CH_2_—P), 3.56 (ddd, *J*(gem) = 11.6 Hz, *J*(CH_2_—CH_2_) = 6.7 and 2.8 Hz, 1H, 1′‐O—CH_2_b), 1.92–2.02 (m, 2H, CH_2_—P), 1.89 (s, 3H, CH_3_—COO), 1.14 (2 × t, *J*(CH_3_—CH_2_) = 7.0 Hz, 2 × 3H, CH_2_—CH_3_). ^13^C NMR (125 MHz, DMSO‐*d*_*6*_): *δ* = 170.37 (COO), 152.38 (C‐4), 152.12 (C‐2), 149.45 (C‐6), 146.28 (C‐8), 131.15 (C‐5), 83.05 (C‐1′), 69.60 (C‐2′), 67.25 (1′‐O—CH_2_), 65.24 (d, *J*(C—P) = 1.8 Hz, CH_2_—CH_2_—P), 62.87 (AcO—CH_2_), 61.07–61.15 (m, CH_2_—CH_3_), 25.96 (d, *J*(C—P) = 137.1 Hz, CH_2_—P), 20.68 (CH_3_—COO), 16.39 (d, *J*(C—P) = 5.9 Hz, CH_2_—CH_3_). HRMS (ESI^+^): *m/z* [*M* + H]^+^ calcd. for C_17_H_26_O_7_N_4_ClNaP = 487.11198, found: 487.11218.

##### Chemical Synthesis: Diethyl (2‐(2‐(2‐acetoxyethoxy)‐2‐(2‐acetamido‐6‐chloro‐9*H*‐purin‐9‐yl)ethoxy)ethyl)phosphonate (12d)

Prepared according to general method A from **6** (540 mg, 2.0 mmol), **9** (420 mg, 2.0 mmol), acetic anhydride (0.2 mL, 200 mg, 2.0 mmol), and TMSOTf (0.5 mL, 666 mg, 3.0 mmol) in dry MeCN (10 mL) to give **12d** (640 mg, 64%) as a yellowish viscous oil. ^1^H NMR (500 MHz, DMSO‐*d*_*6*_): *δ* = 10.88 (bs, 1H, NH), 8.71 (s, 1H, H‐8), 5.90 (dd, *J*(1′‐2′) = 6.8 and 5.1 Hz, H‐1′), 4.08–4.15 (m, 2H, H‐2′b, AcO—CH_2_a), 3.96–4.00 (m, 2H, H‐2′a, AcO—CH_2_b), 3.84–3.91 (m, 4H, CH_2_—CH_3_), 3.79 (ddd, *J*(gem) = 11.6 Hz, *J*(CH_2_—CH_2_) = 5.9 and 2.7 Hz, 1H, 1′‐O—CH_2_a), 3.56–3.70 (m, 3H, 1′‐O—CH_2_b, CH_2_—CH_2_—P), 2.20 (s, 3H, CH_3_—CON), 1.95–2.02 (m, 2H, CH_2_—P), 1.91 (s, 3H, CH_3_—COO), 1.13–1.16 (m, 6H, CH_2_—CH_3_). ^13^C NMR (125 MHz, DMSO‐*d*_*6*_): *δ* = 170.40 (COO), 169.03 (CON), 153.21 (C‐4), 152.43 (C‐2), 149.39 (C‐6), 145.17 (C‐8), 127.41 (C‐5), 82.89 (C‐1′), 69.32 (C‐2′), 67.20 (1′‐O—CH_2_), 65.19 (d, *J*(C—P) = 1.7 Hz, CH_2—_CH_2—_P), 62.83 (1′‐O—CH_2—_CH_2_), 61.09–61.16 (m, CH_2_—CH_3_), 25.96 (d, *J*(C—P) = 137.0 Hz, CH_2—_P), 24.86 (CH_3_—CON), 20.69 (CH_3_—COO), 16.39 (d, *J*(C—P) = 5.9 Hz, CH_2_—CH_3_). HRMS (ESI^+^): *m/z* [*M* + H]^+^ calcd. for C_19_H_30_O_8_N_5_ClP = 522.15150, found: 522.15153.

##### Chemical Synthesis: Diethyl (2‐(2‐(2‐acetoxyethoxy)‐2‐(2,6‐dichloro‐9*H*‐purin‐9‐yl)ethoxy)ethyl)phosphonate (12e)

Prepared according to general method A from **6** (3.0 g, 11 mmol), **10** (2.1 g, 11 mmol), acetic anhydride (1.6 mL, 1.7 g, 17 mmol), and SnCl_4_ (3.9 mL, 8.7 g, 33 mmol) in dry MeCN (25 mL) to give **12e** (2.3 g, 41%) as a yellow oil. ^1^H NMR (500 MHz, DMSO‐*d*_*6*_): *δ* = 8.92 (s, 1H, H‐8), 6.01 (dd, *J*(1′‐2′) = 6.5 and 4.8 Hz, 1H, H‐1′), 3.86–4.12 (m, 8H, AcO—CH_2_, H‐2′, CH_2_—CH_3_), 3.81 (m, 1H, 1′‐O—CH_2_a), 3.57–3.67 (m, 3H, 1′‐O—CH_2_b, CH_2_—CH_2_—P), 1.92–2.03 (m, 2H, CH_2_—P), 1.93 (s, 3H, CH_3—_COO), 1.14–1.18 (m, 6H, CH_2—_CH_3_). ^13^C NMR (125 MHz, DMSO‐*d*_*6*_): *δ* = 170.36 (COO), 153.84 (C‐4), 151.47 (C‐6), 150.03 (C‐2), 147.09 (C‐8), 130.84 (C‐5), 83.30 (C‐1′), 69.61 (C‐2′), 67.31 (1′‐O—CH_2_), 65.27 (d, *J*(C—P) = 1.9 Hz, CH_2_—CH_2_—P), 62.84 (AcO—CH_2_), 61.07–61.15 (m, CH_2_—CH_3_), 25.94 (d, *J*(C—P) = 137.3 Hz, CH_2_—P), 20.70 (CH_3_—COO), 16.35–16.40 (m, CH_2_—CH_3_). HRMS (ESI^+^): *m/z* [*M* + H]^+^ calcd. for C_17_H_26_O_7_N_4_Cl_2_P = 499.09107, found: 499.09121.

##### Chemical Synthesis: Diethyl (2‐(2‐(2‐acetoxyethoxy)‐2‐(4‐chloro‐1*H*‐pyrazolo[3,4‐d]pyrimidin‐1‐yl)ethyl)phosphonate (12f)

Prepared according to general method A from **6** (1.0 g, 3.8 mmol), **11** (580 mg, 3.8 mmol), acetic anhydride (0.5 mL, 570 mg, 5.63 mmol), and SnCl_4_ (0.4 mL, 960 mg, 3.8 mmol) in dry MeCN (5 mL) to give **12f** (557 mg, 32%) as a yellow oil. ^1^H NMR (500 MHz, DMSO‐*d*_*6*_): *δ* = 8.93 (s, 1H, H‐2), 8.62 (s, 1H, H‐7), 6.20 (dd, *J*(1′‐2′a) = 7.6 Hz, *J*(1′‐2′b) = 5.1 Hz, 1H, H‐1′), 4.12 (dd, *J*(gem) = 10.5 Hz, *J*(2′a‐1′) = 7.6 Hz, 1H, H‐2′a), 4.07 (ddd, *J*(gem) = 12.3 Hz, *J*(CH_2—_CH_2_) = 6.4 and 2.9 Hz, 1H, AcO—CH_2_a), 4.02 (dd, *J*(gem) = 10.5 Hz, *J*(2′b‐1′) = 5.1 Hz, 1H, H‐2′b), 3.94 (ddd, *J*(gem) = 12.3 Hz, *J*(CH_2_—CH_2_) = 6.3 and 2.9 Hz, 1H, AcO—CH_2_b), 3.81–3.89 (m, 4H, CH_2_—CH_3_), 3.73 (ddd, *J*(gem) = 11.6 Hz, *J*(CH_2_—CH_2_) = 6.4 and 2.9 Hz, 1H, 1′‐O—CH_2_a), 3.62 and 3.53 (2 × m, 2 × 1H, CH_2_—CH_2—_P), 3.44 (ddd, *J*(gem) = 11.6 Hz, *J*(CH_2_—CH_2_) = 6.4 and 2.9 Hz, 1H, 1′‐O—CH_2_b), 1.88–1.95 (m, 2H, CH_2_—P), 1.91 (s, 3H, CH_3_—COO), 1.14 and 1.13 (2 × t, *J*(CH_3_—CH_2_) = 7.0 Hz, 2 × 3H, CH_2_—CH_3_). ^13^C NMR (125 MHz, DMSO‐*d*_*6*_): *δ* = 170.38 (COO), 155.39 (C‐2), 154.66 (C‐4), 154.04 (C‐6), 133.85 (C‐7), 113.67 (C‐5), 84.22 (C‐1′), 69.36 (C‐2′), 66.77 (1′‐O—CH_2_), 65.09 (d, *J*(C—P) = 1.5 Hz, CH_2_—CH_2_—P), 62.79 (AcO—CH_2_), 61.03–61.11 (m, CH_2_—CH_3_), 25.99 (d, *J*(C—P) = 136.8 Hz, CH_2_—P), 20.73 (CH_3_—COO), 16.34–16.40 (m, CH_2_—CH_3_). HRMS (ESI^+^): *m/z* [*M* + H]^+^ calcd. for C_17_H_27_O_7_N_4_ClP = 465.13004, found: 465.13023.

##### Chemical Synthesis: Diisopropyl ((3‐(3‐acetoxypropoxy)‐3‐(6‐chloro‐9*H*‐purin‐9‐yl)propoxy)methyl)phosphonate (12g)

Prepared according to general method A from **7** (5.0 g, 16 mmol), **8** (2.5 g, 16 mmol), acetic anhydride (2.3 mL, 2.5 g), and SnCl_4_ (2.0 mL, 4.5 g, 17 mmol) in dry MeCN (30 mL) to give **12g** (3.5 g, 44%) as a colorless viscous oil. ^1^H NMR (500 MHz, DMSO‐*d*_*6*_): *δ* = 8.92 (s, 1H, H‐8), 8.80 (s, 1H, H‐2), 5.92 (dd, *J*(1′‐2′) = 7.5 and 5.9 Hz, 1H, H‐1′), 4.50–4.61 (m, 2H, CH*iPr*), 3.97 (dt, *J*(gem) = 10.9 Hz, *J*(CH_2_—CH_2_) = 6.1 Hz, 1H, AcO—CH_2_a), 3.88 (m, 1H, AcO—CH_2_b), 3.62–3.71 (m, 3H, CH_2_—P, H‐3′a), 3.51–3.56 (m, 2H, H‐3′b, 1′‐O—CH_2_a), 3.29 (m, 1H, 1′‐O—CH_2_b), 2.39 and 2.53 (2 × m, 2 × 1H, H‐2′), 1.86 (s, 3H, CH_3_—COO), 1.67–1.78 (m, 2H, 1′‐O—CH_2_—CH_2_), 1.21–1.24 (m, 12H, CH_3_ *iPr*). ^13^C NMR (125 MHz, DMSO‐*d*_*6*_): *δ* = 170.33 (COO), 152.17 (C‐4), 151.98 (C‐2), 149.41 (C‐6), 146.03 (C‐8), 131.26 (C‐5), 82.54 (C‐1′), 70.29 (d, *J*(C—P) = 6.4 Hz, CH*iPr*), 68.20 (d, *J*(C—P) = 11.50 Hz, C‐3′), 65.14 (1′‐O—CH_2_), 65.03 (d, *J*(C—P) = 164.30 Hz, CH_2_—P), 60.70 (AcO—CH_2_), 34.27 (C‐2′), 28.06 (1′‐O—CH_2_—CH_2_), 23.88–24.01 (m, CH_3_ *iPr*), 20.66 (CH_3_—COO). HRMS (ESI^+^): *m/z* [*M* + H]^+^ calcd. for C_20_H_33_O_7_N_4_ClP = 507.17699, found: 507.17723.

##### Chemical Synthesis: Diisopropyl ((3‐(3‐acetoxypropoxy)‐3‐(2‐acetamido‐6‐chloro‐9*H*‐purin‐9‐yl)propoxy)methyl)phosphonate (12 h)

Prepared according to general method A from **7** (5.0 g, 16 mmol), **9** (3.4 g, 16 mmol), acetic anhydride (2.3 mL, 2.5 g, 16 mmol), and SnCl_4_ (1.9 mL, 4.2 g, 16 mmol) in dry MeCN (25 mL) to give **12 h** (2.8 g, 31%) as a yellow viscous oil. ^1^H NMR (500 MHz, DMSO‐*d*_*6*_): *δ* = 10.82 (s, 1H, NH), 8.70 (s, 1H, H‐8), 5.78 (dd, *J*(1′‐2′) = 7.5 and 5.9 Hz, 1H, H‐1′), 4.51–4.62 (m, 2H, CH*iPr*), 3.87–3.99 (m, 2H, AcO—CH_2_), 3.62–3.72 (m, 3H, CH_2_—P, H‐3′a), 3.51–3.56 (m, 2H, H‐3′b, 1′‐O—CH_2_a), 3.45 (m, 1H, 1′‐O—CH_2_b), 2.40 and 2.53 (2 × m, 2 × 1H, H‐2′), 2.19 (s, 3H, CH_3_—CON), 1.87 (s, 3H, CH_3_—COO), 1.68–1.79 (m, 2H, 1′‐O—CH_2_—CH_2_), 1.20–1.23 (m, 12H, CH_3_ *iPr*). ^13^C NMR (125 MHz, DMSO‐*d*_*6*_): *δ* = 170.36 (CH_3_—COO), 169.01 (CH_3_—CON) 153.00 (C‐4), 152.35, and 149.36 (C‐2, C‐6), 144.96 (C‐8), 127.56 (C‐5), 82.35 (C‐1′), 70.32 (d, *J*(C—P) = 6.4 Hz, CH*iPr*), 68.31 (d, *J*(C—P) = 11.3 Hz, C‐3′), 65.12 (1′‐O—CH_2_), 65.09 (d, *J*(C—P) = 164.4 Hz, CH_2_—P), 60.76 (CH_2_—OAc), 33.96 (C‐2′), 28.09 (1′‐O—CH_2_—CH_2_), 24.79 (CH_3_—CON), 23.86–24.00 (m, CH_3_ *iPr*), 20.67 (CH_3_—COO). HRMS (ESI^+^): *m/z* [*M* + H]^+^ calcd. for C_22_H_36_O_8_N_5_ClP = 564.19845, found: 564.19834.

##### Chemical Synthesis: Diisopropyl ((3‐(3‐acetoxypropoxy)‐3‐(4‐chloro‐1*H*‐pyrazolo[3,4‐d]pyrimidin‐1‐yl)propoxy)methyl)phosphonate (12i)

Prepared according to method A from **7** (2.0 g, 6.5 mmol), **11** (1.0 g, 6.5 mmol), acetic anhydride (0.9 mL, 1.0 g, 9.8 mmol), and SnCl_4_ (1.1 mL, 2.5 g, 9.7 mmol) in dry MeCN (20 mL) to give **12i** (990mg, 30%) as a yellow oil. ^1^H NMR (500 MHz, DMSO‐*d*_*6*_): *δ* = 8.91 (s, 1H, H‐2), 8.59 (s, 1H, H‐7), 6.12 (t, *J*(1′‐2′) = 6.6 Hz, 1H, H‐1′), 4.52–4.60 (m, 2H, CH*iPr*), 3.84 and 3.94 (2 × m, 2 × 1H, AcO–CH_2_), 3.67 (d, *J*(CH_2_—P) = 8.2 Hz, 2H, CH_2_—P), 3.61 (m, 1H, H‐3′a), 3.52 (m, 1H, 1′‐O—CH_2_a), 3.45 (m, 1H, H‐3′b), 3.21 (m, 1H, 1′‐O—CH_2_b), 2.37, and 2.51 (2 × m, 2 × 1H, H‐2′), 1.86 (s, 3H, CH_3_—COO), 1.62–1.74 (m, 2H, 1′‐O—CH_2_—CH_2_), 1.21–1.24 (m, 12H, CH_3_ *iPr*). ^13^C NMR (125 MHz, DMSO‐*d*_*6*_): *δ* = 170.32 (COO), 155.23 (C‐2), 154.18 (C‐4), 153.96 (C‐6), 133.60 (C‐7), 113.59 (C‐5), 83.45 (C‐1′), 70.26, and 70.28 (2 × d, *J*(C—P) = 6.4 Hz, CH*iPr*), 68.25 (d, *J*(C—P) = 11.8 Hz, C‐3′), 65.01 (d, *J*(C—P) = 164.4 Hz, CH_2—_P), 64.92 (1′‐O—CH_2_), 60.78 (AcO—CH_2_), 33.76 (C‐2′), 28.12 (1′‐O—CH_2_–CH_2_), 23.88–24.02 (m, CH_3_ *iPr*), 20.67 (CH_3_—COO). MS (ESI) m/z [M + H]^+^ 507.3. HRMS (ESI^+^): *m/z* [*M* + H]^+^ not observed.

##### Chemical Synthesis: Diethyl (2‐(2‐(6‐chloro‐9*H*‐purin‐9‐yl)‐2‐(2‐hydroxyethoxy)ethoxy)ethyl)phosphonate (13a)

A mixture of compound **12c** (950 mg, 2.0 mmol) and dibutyltin oxide (50 mg, 0.20 mmol) in dry methanol (7 mL) was heated in the microwave reactor (100 °C, 200 W) for 45 min. After cooling, the volatiles were removed in vacuo and the crude product was purified by flash chromatography (silica gel, chloroform to 20% methanol for 20 column volumes) to give **13a** (670 mg, 78%) as a colorless viscous oil. ^1^H NMR (500 MHz, DMSO‐*d*_*6*_): *δ* = 8.90 (s, 1H, H‐8), 8.82 (s, 1H, H‐2), 6.05 (dd, *J*(1′‐2′a) = 6.8 Hz, *J*(1′‐2′b) = 4.8 Hz, 1H, H‐1′), 4.66 (t, *J*(OH—CH_2_) = 5.4 Hz, 1H, OH), 4.10 (dd, *J*(gem) = 10.8 Hz, *J*(2′a‐1′) = 6.8 Hz, 1H, H‐2′a), 3.98 (dd, *J*(gem) = 10.8 Hz, *J*(2′b‐1′) = 4.8 Hz, 1H, H‐2′b), 3.83–3.90 (m, 4H, CH_2_—CH_3_), 3.56–3.68 (m, 3H, CH_2_—CH_2_—P, 1′‐O—CH_2_a), 3.39–3.48 (m, 2H, CH_2_—OH), 3.34 (m, 1H, 1′‐O—CH_2_b), 1.90–2.03 (m, 2H, CH_2_—P), 1.14, and 1.13 (2 × t, *J*(CH_3_—CH_2_) = 7.1 Hz, 2 × 3H, CH_3_). ^13^C NMR (125 MHz, DMSO‐*d*_*6*_): *δ* = 152.39 (C‐4), 152.08 (C‐2), 149.40 (C‐6), 146.30 (C‐8), 131.12 (C‐5), 83.26 (C‐1′), 71.13 (1′‐O‐CH_2_), 69.76 (C‐2′), 65.20 (d, *J*(C—P) = 1.7 Hz, CH_2_—CH_2—_P), 61.08–61.16 (m, CH_2_—CH_3_), 59.92 (CH_2_—OH), 25.95 (d, *J*(C—P) = 137.1 Hz, CH_2_—P), 16.39 (d, *J*(C—P) = 5.9 Hz, CH_3_). HRMS (ESI^+^): *m/z* [*M* + H]^+^ calcd. for C_15_H_24_O_6_N_4_ClNaP = 445.10142, found: 445.10163.

##### Chemical Synthesis: Diethyl (2‐(2‐(2‐acetamido‐6‐chloro‐9*H*‐purin‐9‐yl)‐2‐(2‐hydroxyethoxy)ethoxy)ethyl)phosphonate (13b)

A mixture of compound **12d** (3.2 g, 6.2 mmol) and dibutyltin oxide (155 mg, 0.62 mmol) in dry methanol (15 mL) was heated in the microwave reactor (100 °C, 200 W) for 45min. After cooling, the volatiles were removed in vacuo and the crude product was purified by flash chromatography (silica gel, chloroform to 20% methanol for 20 column volumes) to give **13b** (1.7 g, 70%) as a colorless viscous oil. ^1^H NMR (500 MHz, DMSO‐*d*_*6*_): *δ* = 10.85 (bs, 1H, NH), 8.69 (s, 1H, H‐8), 5.89 (dd, *J*(1′‐2′a) = 6.8 Hz, *J*(1′‐2′b) = 4.9 Hz, 1H, H‐1′), 4.64 (t, *J*(OH‐CH_2_) = 5.5 Hz, 1H, OH), 4.13 (dd, *J*(gem) = 10.8 Hz, *J*(2′a‐1′) = 6.8 Hz, 1H, H‐2′a), 3.97 (dd, *J*(gem) = 10.8 Hz, *J*(2′b‐1′)= 4.9 Hz, 1H, H‐2′b), 3.84–3.91 (m, 4H, CH_2_—CH_3_), 3.55–3.69 (m, 3H, CH_2_—CH_2_—P, 1′‐O—CH_2_a), 3.41–3.48 (m, 2H, CH_2_—OH), 3.37 (m, 1H, 1′‐O—CH_2_b), 2.20 (s, 3H, CH_3_—CO), 1.92–2.02 (m, 2H, CH_2_—P), 1.13–1.16 (m, 6H, CH_2_—CH_3_). ^13^C NMR (125 MHz, DMSO‐*d*_*6*_): *δ* = 169.02 (CH_3_—CO), 153.20 (C‐4), 152.36, and 149.32 (C‐2, C‐6), 145.17 (C‐8), 127.39 (C‐5), 83.10 (C‐1′), 71.01 (1′‐O‐CH_2_), 69.49 (C‐2′), 65.14 (d, *J*(C—P) = 1.7 Hz, CH_2_—CH_2_—P), 61.08–61.14 (CH_2_—CH_3_), 59.91 (CH_2_—OH), 25.95 (d, *J*(C—P) = 137.1 Hz, CH_2_—P), 24.81 (CH_3_—CO), 16.35 (d, *J*(C—P) = 5.8 Hz, CH_2_—CH_3_). HRMS (ESI^+^): *m/z* [*M* + H]^+^ calcd. for C_17_H_28_O_7_N_5_ClP = 480.14094, found: 480.14102.

##### Chemical Synthesis: Diisopropyl ((2‐(2‐hydroxyethoxy)‐2‐(hypoxanthin‐9‐yl)ethoxy)methyl)phosphonate (14a)

Prepared according to general method B from **12a** (4.8 g, 10 mmol), DABCO (2.0 g, 18 mmol), K_2_CO_3_ (4.0 g, 30 mmol) under reflux in water (40 mL) for 1 h to give **14a** (2.1 g, 50%) as a yellowish viscous oil. ^1^H NMR (500 MHz, DMSO‐*d*_*6*_): *δ* = 8.14 (s, 1H, H‐2), 8.10 (s, 1H, H‐8), 5.94 (t, *J*(1′‐2′) = 5.1 Hz, 1H, H‐1′), 4.66–4.73 (m, 2H, CH*iPr*), 4.04–4.10 (m, 2H, H‐2′), 3.83–3.92 (m, 2H, CH_2_—P), 3.66–3.75 (m, 3H, CH_2_—OH, 1′‐O—CH_2_a), 3.55 (m, 1H, 1′‐O—CH_2_b), 1.30, 1.29, 1.27, and 1.26 (4 × d, *J*(CH_3_—CH) = 6.2 Hz, 4 × 3H, CH_3_ *iPr*). ^13^C NMR (125 MHz, DMSO‐*d*_*6*_): *δ* = 158.30 (C‐6), 148.76 (C‐4), 146.12 (C‐2), 138.54 (C‐8), 124.34 (C‐5), 83.39 (C‐1′), 73.23 (d, *J*(C—P) = 10.5 Hz, C‐2′), 71.44 (d, *J*(C—P) = 6.8 Hz, CH*iPr*), 71.26 (1′‐O—CH_2_), 66.5 (d, *J*(C—P) = 168.3 Hz, CH_2—_P), 60.97 (CH_2—_OH), 23.85–23.99 (m, CH_3_ *iPr*). HRMS (ESI^+^): *m/z* [*M* + H]^+^ calcd. for C_16_H_27_O_7_N_4_NaP = 441.15096, found: 441.15115.

##### Chemical Synthesis: Diisopropyl ((2‐(2‐hydroxyethoxy)‐2‐(guanin‐9‐yl)ethoxy)methyl)phosphonate (14b)

Prepared according to general method B from **12b** (4.4 g, 8.2 mmol), DABCO (1.0 g, 8.9 mmol), K_2_CO_3_ (3.6g, 26 mmol) under reflux in water (15 mL) for 1 h to give **14b** (1.3 g, 39%) as a transparent viscous oil. ^1^H NMR (500 MHz, DMSO‐*d*_*6*_): *δ* = 10.78 (bs, 1H, NH), 7.83 (s, 1H, H‐8), 6.58 (bs, 2H, NH_2_), 5.64 (dd, *J*(1′‐2′a) = 6.6 Hz, *J*(1′‐2′b) = 5.1 Hz, 1H, H‐1′), 4.45–4.53 (m, 2H, CH*iPr*), 4.04 (dd, *J*(gem) = 10.7 Hz, *J*(2′a‐1′) = 6.6 Hz, H‐2′a), 3.96 (dd, *J*(gem) = 10.7 Hz, *J*(2′b‐1′) = 5.1 Hz, H‐2′b), 3.75–3.85 (m, 2H, CH_2_—P), 3.42–3.50 (m, 3H, CH_2_—OH, 1′‐O—CH_2_a), 3.32 (m, 1H, 1′‐O—CH_2_b), 1.11–1.18 (m, 12H, CH_3_ *iPr*). ^13^C NMR (125 MHz, DMSO‐*d*_*6*_): *δ* = 157.22 (C‐6), 154.06 (C‐2), 151.83 (C‐4), 135.79 (C‐8), 116.58 (C‐5), 81.71 (C‐1′), 72.22 (d, *J*(C—P) = 12.0 Hz, C‐2′), 70.59, and 70.57 (2 × d, *J*(C—P) = 6.4 Hz, CH*iPr*), 70.47 (1′‐O—CH_2_), 65.36 (d, *J*(C—P) = 164.2 Hz, CH_2_—P), 59.92 (CH_2_—OH), 23.75–23.98 (m, CH_3_ *iPr*). HRMS (ESI^+^): *m/z* [*M* + H]^+^ calcd. for C_16_H_29_O_7_N_5_P = 434.17991, found: 434.18007.

##### Chemical Synthesis: Diethyl (2‐(2‐(2‐hydroxyethoxy)‐2‐(hypoxanthin‐9‐yl)ethoxy)ethyl)phosphonate (14c)

Prepared according to general method B from **12c** (5.0 g, 11 mmol), DABCO (1.8 g, 16 mmol), K_2_CO_3_ (6.0 g, 43 mmol) in water (50 mL) at 50 °C for 2 h to obtain **14c** (3.2 g, 74%) as a brownish viscous oil. ^1^H NMR (500 MHz, DMSO‐*d*_*6*_): *δ* = 8.25 (s, 1H, H‐8), 8.07 (s, 1H, H‐2), 5.83 (dd, *J*(1′‐2′) = 6.6 and 4.9 Hz, 1H, H‐1′), 3.87–4.03 (m, 6H, H‐2′, CH_2_—CH_3_), 3.57–3.68 (m, 2H, CH_2_—CH_2_—P), 3.52 (m, 1H, 1′‐O—CH_2_a), 3.40–3.48 (m, 2H, CH_2_—OH), 1.95–2.02 (m, 2H, CH_2_—P), 3.30 (m, 1H, 1′‐O—CH_2_b), 1.15–1.18 (m, 6H, CH_3_). ^13^C NMR (125 MHz, DMSO‐*d*_*6*_): *δ* = 157.19 (C‐6), 148.90 (C‐4), 146.42 (C‐2), 138.78 (C‐8), 124.05 (C‐5), 82.50 (C‐1′), 70.63 (1′‐O—CH_2_), 70.11 (C‐2′), 65.12 (d, *J*(C—P) = 1.6 Hz, CH_2_—CH_2_—P), 61.11–61.17 (m, CH_2_—CH_3_), 59.86 (CH_2_—OH), 26.01 (d, *J*(C—P) = 136.9 Hz, CH_2_—P), 16.36–16.42 (m, CH_3_). HRMS (ESI^+^): *m/z* [*M* + H]^+^ calcd. for C_15_H_26_O_7_N_4_P = 405.15336, found: 405.15356.

##### Chemical Synthesis: Diethyl (2‐(2‐(2‐hydroxyethoxy)‐2‐(guanin‐9‐yl)ethoxy)ethyl)phosphonate (14d)

Prepared according to general method B from **12d** (2.3 g, 4.3 mmol) and K_2_CO_3_ (920 mg, 6.7 mmol) under reflux in water (2 mL) for 20 min, then DABCO (260 mg, 2.3 mmol) and K_2_CO_3_ (920 mg, 6.7 mmol) were added and refluxed for 20 min. Then DABCO (260 mg, 2.3 mmol) was added again and the mixture was refluxed for an additional 20 min to give **14d** (840 mg, 47%) as a brownish oil. ^1^H NMR (500 MHz, DMSO‐*d*_*6*_): *δ* = 10.72 (bs, 1H, H‐1), 7.84 (s, 1H, H‐8), 6.54 (bs, 2H, NH_2_), 5.59 (m, 1H, H‐1′), 3.84–3.97 (m, 6H, H‐2′, CH_2_—CH_3_), 3.56–6.65 (m, 2H, CH_2_—CH_2_—P), 3.28–3.48 (m, 4H, 1′‐O—CH_2_, CH_2_—OH), 1.96–2.03 (m, 2H, CH_2_—P), 1.16–1.19 (m, 6H, CH_3_). ^13^C NMR (125 MHz, DMSO‐*d*_*6*_): *δ* = 156.98 (C‐6), 153.97 (C‐2), 151.74 (C‐4), 135.79 (C‐8), 116.50 (C‐5), 81.84 (C‐1′), 70.36 (1′‐O—CH_2_), 70.28 (C‐2′), 65.09 (d, *J*(C—P) = 1.7 Hz, CH_2_—CH_2_—P), 61.15–61.21 (m, CH_2_—CH_3_), 59.87 (CH_2_—OH), 26.05 (d, *J*(C—P) = 136.7 Hz, CH_2_—P), 16.39–16.43 (m, CH_3_). HRMS (ESI^+^): *m/z* [*M* + H]^+^ calcd. for C_15_H_27_O_7_N_5_P = 420.16426, found: 420.16439.

##### Chemical Synthesis: Diethyl (2‐(2‐(2‐hydroxyethoxy)‐2‐(2‐chloro‐6‐oxopurin‐9‐yl)ethyl)phosphonate (14e)

Prepared according to general method B from **12e** (1.9 g, 3.8 mmol), DABCO (240 mg, 2.1 mmol), K_2_CO_3_ (520 mg, 3.8 mmol) in water (15 mL) at 50 °C for 30 min. Then DABCO (240 mg, 2.1 mmol) and K_2_CO_3_ (520 mg, 3.8 mmol) were added and the mixture was refluxed for 30 min to give **14e** (960 mg, 58%) as a yellowish viscous oil. ^1^H NMR (500 MHz, DMSO‐*d*_*6*_): *δ* = 10.8 (bs, 1H, NH), 8.02 (s, 1H, H‐8), 5.69 (dd, *J*(1′‐2′) =6.5 and 5.2 Hz, 1H, H‐1′), 3.85–3.99 (m, 6H, H‐2′, CH_2_—CH_3_), 3.56–3.67 (m, 2H, CH_2_—CH_2_—P), 3.40–3.50 (m, 3H, CH_2_—OH, 1′‐O—CH_2_a), 3.27 (ddd, *J*(gem) = 9.7 Hz, *J*(CH_2—_CH_2_) = 5.4 and 4.4 Hz, 1H, 1′‐O—CH_2_b), 1.94–2.03 (m, 2H, CH_2_—P), 1.15–1.19 (m, 6H, CH_3_). ^13^C NMR (125 MHz, DMSO‐*d*_*6*_): *δ* = 150.49 (C‐4), 137.25 (C‐8), 122.80 (C‐5), 82.13 (C‐1′), 70.44 (1′‐O—CH_2_), 70.22 (C‐2′), 65.10 (CH_2_—CH_2_—P), 61.12–61.18 (m, CH_2_—CH_3_), 59.88 (CH_2_—OH), 26.03 (d, *J*(C—P) = 136.8 Hz, CH_2_—P), 16.37–16.42 (m, CH_3_), C‐2 and C‐6 not found. HRMS (ESI^+^): *m/z* [*M* + H]^+^ calcd. for C_15_H_24_O_7_N_4_ClNaP = 461.09633, found: 461.09641.

##### Chemical Synthesis: Diethyl (2‐(2‐(2‐hydroxyethoxy)‐2‐(4‐oxo‐4,5‐dihydro‐1H‐pyrazolo[3,4‐d]pyrimidin‐1‐yl)ethyl)phosphonate (14f)

Prepared according to general method B from **12f** (965 mg, 2.1 mmol), DABCO (233 g, 2.1 mmol), K_2_CO_3_ (574 mg, 4.2 mmol) under reflux in water (15 mL) for 1 h to give **14f** (320 mg, 38%) as a yellowish viscous oil. ^1^H NMR (500 MHz, DMSO‐*d*_*6*_): *δ* = 12.31 (bs, 1H, NH), 8.16 (d, *J*(7‐1′) = 0.6 Hz, 1H, H‐7), 8.13 (s, 1H, H‐2), 5.95 (ddd, *J*(1′‐2′a) = 7.5 Hz, *J*(1′‐2′b) = 5.0 Hz, *J*(1′−7) = 0.6 Hz, 1H, H‐1′), 4.63 (bs, 1H, OH), 4.04 (dd, *J*(gem) = 10.4 Hz, *J*(2′a‐1′) = 7.5 Hz, 1H, H‐2′a), 3.96 (dd, *J*(gem) = 10.4 Hz, *J*(2′b‐1′) = 5.1 Hz, 1H, H‐2′b), 3.84–3.92 (m, 4H, CH_2_—CH_3_), 3.61 and 3.52 (2 × m, 2 × 1H, CH_2_—CH_2_—P), 3.39–3.48 (m, 3H, CH_2_—OH, 1′‐O—CH_2_a), 3.21 (m, 1H, 1′‐O—CH_2_b), 1.94 (dt, *J*(CH_2_—P) = 18.3 Hz, *J*(CH_2_—CH_2_) = 7.2 Hz, CH_2_—P), 1.14–1.17 (m, 6H, CH_3_). ^13^C NMR (125 MHz, DMSO‐*d*_*6*_): *δ* = 157.44 (C‐6), 153.77 (C‐4), 148.81 (C‐2), 135.70 (C‐7), 106.14 (C‐5), 83.67 (C‐1′), 70.22 (1′‐O—CH_2_), 69.71 (C‐2′), 65.00 (d, *J*(C—P) = 1.5 Hz, CH_2_—CH_2_—P), 61.08–61.16 (CH_2_—CH_3_), 59.78 (CH_2_—OH), 26.06 (d, *J*(C—P) = 136.8 Hz, CH_2_—P), 16.39 and 16.38 (2 × d, *J*(C–P) = 5.8 Hz, CH_3_). HRMS (ESI^+^): *m/z* [*M* + H]^+^ calcd. for C_15_H_26_O_7_N_4_P = 405.15336, found: 405.15356.

##### Chemical Synthesis: Diisopropyl ((3‐(3‐hydroxypropoxy)‐3‐(hypoxanthin‐9‐yl)propoxy)methyl)phosphonate (14g)

Prepared according to general method B from **12g** (3.3 g, 6.5 mmol), DABCO (500 mg, 4.5 mmol), K_2_CO_3_ (550 mg, 4.0 mmol) under reflux in water (20 mL) for 20 min. Then DABCO (500 mg, 4.5 mmol) and K_2_CO_3_ (550 mg, 4.0 mmol) were added and the mixture was refluxed for 1 h to give **14g** (1.0 g, 34%) as a brownish viscous oil. ^1^H NMR (500 MHz, DMSO‐*d*_*6*_): *δ* = 12.36 (bs, 1H, NH), 8.27 (s, 1H, H‐8), 8.05 (s, 1H, H‐2), 5.74 (dd, *J*(1′‐2′) = 7.7 and 5.6 Hz, 1H, H‐1′), 4.53–4.63 (m, 2H, CH*iPr*), 4.38 (m, 1H, OH), 3.66–3.74 (m, 2H, CH_2—_P), 3.62 (ddd, *J*(gem) = 9.8 Hz, *J*(3′a‐2′) = 7.7 and 5.2 Hz, 1H, H‐3′a), 3.44–3.52 (m, 2H, H‐3′b, 1′‐O—CH_2_a), 3.38 (m, 2H, CH_2_—OH), 3.27 (m, 1H, 1′‐O—CH_2_b), 2.25 and 2.44 (2 × m, 2 × 1H, H‐2′), 1.57 (p, *J*(CH_2—_CH_2_) = 6.4 Hz, 2H, 1′‐O—CH_2_—CH_2_), 1.22–1.25 (m, 12H, CH_3_ *iPr*). ^13^C NMR (125 MHz, DMSO‐*d*_*6*_): *δ* = 156.85 (C‐6), 148.62 (C‐4), 146.02 (C‐2), 138.60 (C‐8), 124.13 (C‐5), 81.66 (C‐1′), 70.35 and 70.36 (2 × d, *J*(C‐P) = 6.3 Hz, CH*iPr*), 68.32 (d, *J*(C—P) = 11.4 Hz, C‐3′), 65.82 (1′‐O—CH_2_), 65.06 (d, *J*(C—P) = 164.1 Hz, CH_2—_P), 57.55 (CH_2—_OH), 34.91 (C‐2′), 32.33 (1′‐O—CH_2_—CH_2_), 23.90–24.04 (m, CH_3_ *iPr*). HRMS (ESI^+^): *m/z* [*M* + H]^+^ calcd. for C_18_H_32_O_7_N_4_P = 447.20031, found: 447.20042.

##### Chemical Synthesis: Diisopropyl ((3‐(guanin‐9‐yl)‐3‐(3‐hydroxypropoxy)propoxy)methyl)phosphonate (14 h)

Prepared according to general method B from **12 h** (2.1 g, 3.8mmol), DABCO (511 mg, 4.6 mmol), K_2_CO_3_ (2.6 g, 19 mmol) under reflux in water (20 mL) for 1 h to give **14 h** (500 mg, 29%) as a brownish viscous oil. ^1^H NMR (500 MHz, DMSO‐*d*_*6*_): *δ* = 10.62 (bs, 1H, NH), 7.84 (s, 1H, H‐8), 6.47 (bs, 2H, NH_2_), 5.52 (dd, *J*(1′‐2′) = 7.9 and 5.6 Hz, 1H, H‐1′), 4.53–4.63 (m, 2H, CH*iPr*), 4.39 (t, *J*(OH—CH_2_) = 5.0 Hz, 1H, OH), 3.67–3.74 (m, 2H, CH_2_—P), 3.60 (ddd, *J*(gem) = 9.8 Hz, *J*(3′‐2′) = 7.7 and 5.5 Hz, 1H, H‐3′a), 3.50 (m, 1H, H‐3′b), 3.26–3.44 (m, 4H, 1′‐O—CH_2_, CH_2_—OH), 2.16 and 2.37 (2 ×  m, 2 × 1H, H‐2′), 1.58 (p, *J*(CH_2_—CH_2_) = 6.4 Hz, 2H, 1′‐O—CH_2_—CH_2_), 1.22–1.25 (m, 12H, CH_3_ *iPr*). ^13^C NMR (125 MHz, DMSO‐*d*_*6*_): *δ* = 157.00 (C‐6), 153.86 (C‐2), 151.50 (C‐4), 135.58 (C‐8), 116.67 (C‐5), 80.86 (C‐1′), 70.39 (d, *J*(C‐P) = 6.4 Hz, CH*iPr*), 68.51 (d, *J*(C‐P) = 11.0 Hz, C‐3′), 65.58 (1′‐O—CH_2_), 65.14 (d, *J*(C—P) = 164.1 Hz, CH_2_—P), 57.67 (CH_2_—OH), 34.89 (C‐2′), 32.41 (1′‐O—CH_2—_CH_2_), 23.91–24.05 (m, CH_3_ *iPr*). HRMS (ESI^+^): *m/z* [*M* + H]^+^ calcd. for C_18_H_33_O_7_N_5_P = 462.21121, found: 462.21136.

##### Chemical Synthesis: Diisopropyl ((3‐(3‐hydroxypropoxy)‐3‐(4‐oxo‐4,5‐dihydro‐1*H*‐pyrazolo[3,4‐d]pyrimidin‐1‐yl)propoxy)methyl)phosphonate (14i)

Prepared according to general method B from **12i** (1.5 g, 3.0 mmol), DABCO (500 mg, 4.5 mmol), K_2_CO_3_ (1.6 g, 12 mmol) under reflux in water (15mL) for 30 min to give **14i** (350 mg, 27%) as a colorless viscous oil. ^1^H NMR (500 MHz, DMSO‐*d*_*6*_): *δ* = 12.30 (bs, 1H, NH), 8.15 (d, J(7‐1′) = 0.7 Hz, 1H, H‐7), 8.11 (s, 1H, H‐2), 5.92 (m, 1H, H‐1′), 4.53–4.63 (m, 2H, CH*iPr*), 4.34 (bt, *J*(OH—CH_2_) = 5.0 Hz, 1H, OH), 3.68 (d, *J*(CH_2—_P) = 8.1 Hz, 2H, CH_2—_P), 3.57 (ddd, *J*(gem) = 9.8 Hz, *J*(3′a‐2′) = 7.4 and 5.2 Hz, 1H, H‐3′a), 3.40–3.47 (m, 2H, H‐3′b, 1′‐O—CH_2_a), 3.28–3.38 (m, 2H, CH_2_—OH), 3.20 (dt, *J*(gem) = 9.6 Hz, *J*(CH_2_—CH_2_) = 6.4 Hz, 1H, 1′‐O—CH_2_b), 2.42 and 2.25 (2 × m, 2 × 1H, H‐2′), 1.54 (p, *J*(CH_2_—CH_2_) = 6.4 Hz, 2H, 1′‐O—CH_2_—CH_2_), 1.22–1.25 (m, 12H, CH_3_ *iPr*). ^13^C NMR (125 MHz, DMSO‐*d*_*6*_): *δ* = 157.52 (C‐6), 153.29 (C‐4), 148.65 (C‐2), 135.57 (C‐7), 106.04 (C‐5), 82.58 (C‐1′), 70.32, and 70.34 (2 × d, *J*(C—P) = 6.4 Hz, CH*iPr*), 68.41 (d, *J*(C—P) = 11.7 Hz, C‐3′), 65.49 (1′‐O—CH_2_), 65.05 (d, *J*(C—P) = 164.2 Hz, CH_2_—P), 57.63 (CH_2_—OH), 34.03 (C‐2′), 32.37 (1′‐O—CH_2_—CH_2_), 23.89–24.04 (m, CH_3_ *iPr*). HRMS (ESI^+^): *m/z* [*M* + H]^+^ calcd. for C_18_H_32_O_7_N_4_P = 447.20031, found: 447.20044.

##### Chemical Synthesis: Diethyl (2‐(2‐(6‐chloro‐9*H*‐purin‐9‐yl)‐2‐(2‐((diisopropoxyphosphoryl)methoxy)ethoxy)ethoxy)ethyl)phosphonate (16a)

Butyl lithium (2.7 M solution in toluene, 0.5 mL, 1.3 mmol) was added to a solution of **13a** (500 mg, 1.2 mmol) in dry THF (20 mL) at −78 °C and the mixture was stirred at −78 °C for 15 min. To this solution, a preformed solution of **15** (390 mg, 1.2 mmol) in dry THF (5 mL) was added and the mixture was allowed to warm up to laboratory temperature. Water (30 mL) was added and the slurry was extracted with EtOAc (3 × 50 mL). The combined organic layers were washed with brine (30 mL), dried over NaSO_4_, and evaporated to give crude product which was further purified by column chromatography (silica gel, chloroform to 20% methanol for 15 column volumes) to give **16a** (500 mg, 70%) as a yellowish viscous oil. ^1^H NMR (500 MHz, DMSO‐*d*_*6*_): *δ* = 8.88 (s, 1H, H‐8), 8.81 (s, 1H, H‐2), 6.04 (dd, *J*(1′‐2′a) = 6.9 Hz, *J*(1′‐2′b) = 4.8 Hz, 1H, H‐1′), 4.51–4.63 (m, 2H, CH‐*iPr*), 4.12 (dd, 1H, *J*(gem) = 10.8 Hz, *J*(2′a‐1′) = 6.9 Hz, H‐2′a), 3.97 (dd, 1H, *J*(gem) = 10.8 Hz, *J*(2′b‐1′) = 4.8 Hz, H‐2′b), 3.82–3.90 (m, 4H, CH_2_—CH_3_), 3.55–3.74 (m, 7H, O—CH_2_—P, CH_2—_CH_2—_P, 1′‐O–—H_2_—CH_2_, 1′‐O–CH_2_a), 3.34 (ddd, *J*(gem) = 11.1 Hz, *J*(CH_2_—CH_2_) = 5.8 and 2.9 Hz, 1H, 1′‐O—CH_2_b), 1.92–2.00 (m, 2H, CH_2_—CH_2_—P), 1.12–1.25 (m, 18H, CH_2_—CH_3_, CH_3_ *iPr*). ^13^C NMR (125 MHz, DMSO‐*d*_*6*_): *δ* = 152.35 (C‐4), 152.08 (C‐2), 149.44 (C‐6), 146.29 (C‐5), 131.19 (C‐5), 83.20 (C‐1′), 71.23 (d, *J*(C—P) = 11.7 Hz, 1′‐O—CH_2_—CH_2_), 70.33 (d, *J*(C—P) = 6.3 Hz, CH*iPr*), 69.54 (C‐2′), 68.39 (1′‐O—CH_2_), 65.19 (CH_2_—CH_2_—P), 64.98 (d, *J*(C—P) = 164.4 Hz, O—CH_2_—P), 61.06–61.14 (m, CH_2_—CH_3_), 25.96 (d, *J*(C—P) = 137.1 Hz, CH_2_—CH_2_—P), 23.87–24.04 (m, CH_3_ *iPr*), 16.36–16.41 (m, CH_2_—CH_3_). HRMS (ESI^+^): *m/z* [*M* + H]^+^ calcd. for C_22_H_39_O_9_N_4_ClNaP_2_ = 623.17730, found: 623.17767.

##### Chemical Synthesis: Diethyl (2‐(2‐(2‐acetamido‐6‐chloro‐9*H*‐purin‐9‐yl)‐2‐(2‐((diisopropoxyphosphoryl)methoxy)ethoxy)ethoxy)ethyl)phosphonate (16b)

Butyl lithium (2.5 M solution in THF, 0.8 mL, 2.1 mmol) was added to a solution of **13b** (500 mg, 1.0 mmol) in dry THF (12 mL) at −78 °C and the mixture was stirred at −78 °C for 15 min. To this solution, a preformed solution of **15** (376 mg, 1.1 mmol) in dry THF (3 mL) was added and the mixture was allowed to warm up to laboratory temperature. Water (30 mL) was added and the slurry was extracted with EtOAc (3 × 50 mL). The combined organic layers were washed with brine (30 mL), dried over NaSO_4_, and evaporated to give crude product which was further purified by column chromatography (silica gel, chloroform to 20% methanol for 15 column volumes) to give **16b** (200 mg, 51%) as a brownish viscous oil. ^1^H NMR (500 MHz, DMSO‐*d*_*6*_): *δ* = 11.95 (bs, 1H, NH), 8.28 (s, 1H, H‐8), 5.71 (dd, 1H, *J*(1′‐2′) = 6.7 Hz, *J*(1′‐2′) = 5.0 Hz, H‐1′), 4.52–4.64 (m, 2H, CH‐*iPr*), 3.86–4.05 (m, 6H, H‐2′, CH_2_—CH_3_), 3.67–3.73 (m, 2H, O—CH_2—_P), 3.53–3.66 (m, 5H, CH_2—_CH_2—_P, 1′‐O—CH_2—_CH_2_, 1′‐O—CH_2_a), 3.45 (m, 1H, 1′‐O—CH_2_b), 1.94–2.01 (m, 2H, CH_2—_CH_2_—P), 1.14–1.25 (m, 18H, CH_2—_CH_3_, CH_3_ *iPr*). ^13^C NMR (125 MHz, DMSO‐*d*_*6*_): *δ* = 172.20 (CON), 160.07 (C‐2), 154.52 (C‐4), 149.69 (C‐6), 141.66 (C‐8), 123.45 (C‐5), 82.31 (C‐1′), 71.23 (d, *J*(C—P) = 11.6 Hz, 1′‐O—CH_2—_CH_2_), 70.33 (d, *J*(C—P) = 6.3 Hz, CH*iPr*), 69.59 (C‐2′), 68.01 (1′‐O—CH_2—_CH_2_), 65.12 (CH_2_—CH_2_—P), 65.01 (d, *J*(C—P) = 164.4 Hz, O—CH_2—_P), 61.08–61.15 (m, CH_2_—CH_3_), 26.09 (d, *J*(C—P) = 136.9 Hz, CH_2—_CH_2_—P), 23.85–24.03 (m, CH_3_ *iPr*), 16.37 (d, *J*(C—P) = 5.9 Hz, CH_2_—CH_3_). HRMS (ESI^+^): *m/z* [*M* + H]^+^ calcd. for C_24_H_43_O_10_N_5_ClP_2_ = 658.21682, found: 658.21696.

##### Chemical Synthesis: Diethyl (2‐(2‐(2‐((diisopropoxyphosphoryl)methoxy)ethoxy)‐2‐(hypoxanthin‐9‐yl)ethoxy)ethyl)phosphonate (17a)

Prepared according to General method B from **16a** (1.1 g, 1.3 mmol), DABCO (200 mg, 1.3 mmol), K_2_CO_3_ (250 mg, 1.3 mmol) under reflux in water (10 mL) for 30 min to give **17a** (620 mg, 59%) as a yellowish viscous oil. ^1^H NMR (500 MHz, DMSO‐*d*_*6*_): *δ* = 12.37 (bs, 1H, H‐1), 8.25 (s, 1H, H‐8), 8.06 (s, 1H, H‐2), 5.82 (dd, *J*(1′‐2′a) = 6.7 Hz, *J*(1′‐2′b) = 5.0 Hz, 1H, H‐1′), 4.51–4.61 (m, 2H, CH*iPr*), 4.02 (dd, *J*(gem) = 10.8 Hz, *J*(2′a‐1′) = 6.8 Hz, 1H, H‐2′a), 3.87–3.95 (m, 5H, H‐2′b, CH_2_—CH_3_), 3.54–3.74 (m, 7H, O—CH_2_—P, CH_2_—CH_2_—P, 1′‐O—CH_2_—CH_2_, 1′‐O—CH_2_a), 3.44 (m, 1H, 1′‐O—CH_2_b), 1.93–2.02 (m, 2H, CH_2_—CH_2_—P), 1.15–1.24 (m, 18H, CH_2_—CH_3_, CH_3_ *iPr*). ^13^C NMR (125 MHz, DMSO‐*d*_*6*_): *δ* = 156.77 (C‐6), 148.77 (C‐4), 146.09 (C‐2), 138.92 (C‐8), 124.15 (C‐5), 82.53 (C‐1′), 71.17 (d, *J*(C—P) = 11.6 Hz, 1′‐O—CH_2_—CH_2_), 70.30 (d, *J*(C—P) = 6.4 Hz, CH*iPr*), 69.89 (C‐2′), 68.03 (1′‐O—CH_2_), 65.10 (CH_2_—CH_2_—P), 65.03 (d, *J*(C—P) = 164.4 Hz, O—CH_2_—P), 61.07–61.13 (m, CH_2_—CH_3_), 26.02 (d, *J*(C—P) = 136.9 Hz, CH_2_—CH_2_—P), 23.83–24.00 (m, CH_3_ *iPr*), 16.35 (d, *J*(C—P) = 5.9 Hz, CH_2_—CH_3_). HRMS (ESI^+^): *m/z* [*M* + H]^+^ calcd. for C_22_H_41_O_10_N_4_P_2_ = 583.22924, found: 583.22937.

##### Chemical Synthesis: Diethyl (2‐(2‐(guanin‐9‐yl)‐2‐(2‐((diisopropoxyphosphoryl)methoxy)ethoxy)ethoxy)ethyl)phosphonate (17b)

Prepared according to general method B from **16b** (790 mg, 1.2 mmol), DABCO (162 mg, 1.4 mmol), K_2_CO_3_ (365 mg, 2.6 mmol) under reflux in water (10 mL) for 45 min to give **17b** (240 mg, 34%) as a brownish amorphous solid. ^1^H NMR (500 MHz, DMSO‐*d*_*6*_): *δ* = 10.62 (bs, 1H, NH), 7.83 (s, 1H, H‐8), 6.48 (bs, 2H, NH_2_), 5.58 (dd, *J*(1′‐2′) = 6.4 and 5.4 Hz, 1H, H‐1′), 4.53–4.60 (m, 2H, CH*iPr*), 3.84–3.95 (m, 6H, H‐2′, CH_2—_CH_3_), 3.67–3.75 (m, 2H, P—CH_2—_O), 3.54–3.65 (m, 5H, CH_2—_CH_2—_P, 1′‐O—CH_2—_CH_2_, 1′‐O—CH_2_a), 3.42 (m, 1H, 1′‐O—CH_2_b), 1.93–2.06 (m, 2H, CH_2—_CH_2—_P), 1.16–1.24 (m, 18H, CH_2—_CH_3_, CH_3_ *iPr*). ^13^C NMR (125 MHz, DMSO‐*d*_*6*_): *δ* = 156.92 (C‐6), 153.92 (C‐2), 151.68 (C‐4), 135.76 (C‐8), 116.58 (C‐5), 81.79 (C‐1′), 71.21 (d, *J*(C—P) = 11.7 Hz, 1′‐O—CH_2—_CH_2_), 70.34 (d, *J*(C—P) = 6.3 Hz, CH*iPr*), 70.05 (C‐2′), 67.71 (1′‐O—CH_2—_CH_2_), 65.08 (CH_2_—CH_2—_P), 65.03 (d, *J*(C—P) = 164.4 Hz, P—CH_2—_O), 61.12–61.17 (m, CH_2—_CH_3_), 26.04 (d, *J*(C—P) = 136.7 Hz, CH_2—_CH_2—_P), 23.86–24.02 (m, CH_3_ *iPr*), 16.39 (d, *J*(C—P) = 5.9 Hz, CH_2—_CH_3_). HRMS (ESI^+^): *m/z* [*M* + H]^+^ calcd. for C_22_H_42_O_10_N_5_P_2_ = 598.24014, found: 598.24035.

##### Chemical Synthesis: Sodium salt of (2‐(2‐(hypoxanthin‐9‐yl)‐2‐(2‐(phosphonatomethoxy)ethoxy)ethoxy)ethyl)phosphonic acid (18a)

Prepared according to general method C from **17a** (290 mg, 0.5 mmol), TMSBr (0.6 mL, 687 mg, 4.5 mmol) in pyridine (13 mL) to give **18a** (88 mg, 34%) as a white solid. ^1^H NMR (500.0 MHz, D_2_O, ref: *t*BuOH): *δ* = 8.35 (s, 1H, H‐8), 8.21 (s, 1H, H‐2), 6.02 (t, *J*(1′‐2′) =5.6 Hz, 1H, H‐1′), 4.03–4.10 (m, 2H, H‐2′), 3.61–3.84 (m, 6H, 1′‐O—CH_2_—CH_2_, CH_2_—CH_2—_P), 3.51 (d, *J*(CH_2_—P) = 8.4 Hz, 2H, O—CH_2—_P), 1.78–1.83 (m, 2H, CH_2—_CH_2—_P). ^13^C NMR (125.7 MHz, D_2_O, ref: *t*BuOH): *δ* = 159.46 (C‐6), 149.63 (C‐4), 146.78 (C‐2), 141.33 (C‐8), 124.42 (C‐5), 84.36 (C‐1′), 71.58 (d, *J*(C—P) = 10.2 Hz, 1′‐O—CH_2—_CH_2_), 70.59 (C‐2′), 69.12 (1′‐O—CH_2_), 68.55 (d, *J*(C—P) = 2.2 Hz, CH_2—_CH_2—_P), 68.51 (d, *J*(C—P) = 153.3 Hz, O—CH_2—_P), 29.87 (d, *J*(C—P) = 127.4 Hz, CH_2—_CH_2—_P). HRMS (ESI^−^): *m/z* [*M−*H]^−^ calcd. for C_12_H_19_O_10_N_4_P_2_ = 441.05709, found: 441.05717.

##### Chemical Synthesis: Sodium salt of (2‐(2‐(guanin‐9‐yl)‐2‐(2‐(phosphonatomethoxy)ethoxy)ethoxy)ethyl)phosphonic acid (18b)

Prepared according to general method C from **17b** (100 mg, 0.2 mmol), TMSBr (0.2 mL, 230 mg, 1.5 mmol) in pyridine (5 mL) to give **18b** (42 mg, 47%) as a white solid. ^1^H NMR (500.0 MHz, D_2_O, ref: *t*BuOH): *δ* = 8.10 (s, 1H, H‐8), 5.92 (t, *J*(1′‐2′) = 5.7 Hz, 1H, H‐1′), 4.05–4.13 (m, 2H, H‐2′), 3.70–3.90 (m, 6H, 1′‐O—CH_2_—CH_2_, CH_2_—CH_2—_P), 3.65 (d, *J*(CH_2—_P) = 8.5 Hz, 2H, O—CH_2—_P), 1.90–2.03 (m, 2H, CH_2—_CH_2—_P). ^13^C NMR (125.7 MHz, D_2_O, ref: *t*BuOH): *δ* = 159.78 (C‐6), 154.65 (C‐2), 152.52 (C‐4), 138.82 (C‐8), 116.81 (C‐5), 83.46 (C‐1′), 71.75 (d, *J*(C‐P) = 10.4 Hz, 1′‐O—CH_2_—CH_2_), 70.60 (C‐2′), 68.78 (1′‐O—CH_2_), 68.13 (d, *J*(C—P) = 2.0 Hz, CH_2_—CH_2—_P), 68.00 (d, *J*(C—P) = 154.8 Hz, O—CH_2—_P), 29.60 (d, *J*(C—P) = 128.8 Hz, CH_2—_CH_2_—P). HRMS (ESI^−^): *m/z* [*M−*H]^−^ calcd. for C_12_H_20_O_10_N_5_P_2_ = 456.06909, found: 456.06866.

##### Chemical Synthesis: Tetra‐(L‐phenylalaninate ethyl ester) prodrug of (2‐(2‐(hypoxanthin‐9‐yl)‐2‐(2‐(phosphonatomethoxy)ethoxy)ethoxy)ethyl)phosphonic acid (19a)

Prepared according to general method D from **17a** (210 mg, 0.36 mmol), pyridine (10 mL) and TMSBr (0.38 mL, 442 mg, 2.88 mmol, 8 eq), then L‐phenylalanine ethyl ester hydrochloride (650 mg, 2.88 mmol, 8 eq), Aldrithiol‐2 (1.14 g, 4.3 mmol, 12 eq), triphenylphosphine (953 mg, 4.32 mmol, 12 eq), dry triethylamine (5 mL), and dry pyridine (10 mL) to give **19a** (27 mg, 7%) as a white solid. ^1^H NMR (500 MHz, DMSO‐*d*_*6*_, mixture of diastereomers): *δ* = 12.33 (bs, 1H, NHpur), 8.19 and 8.20 (s, 1H, H‐8), 8.05 and 8.06 (s, 1H, H‐2), 7.05–7.26 (m, 20H, H‐2′′, H‐3′′, H‐4′′), 5.73–5.79 (m, 1H, H‐1′), 4.44–4.50 (m, 2H, 2 × NH), 3.69–4.12 (m, 16H, CH_2_—CH_3_, 2 × NH, NH—CH, H‐2′), 3.14–3.56 (m, 8H, 1′‐O—CH_2_—CH_2_, CH_2_—CH_2_—P, O—CH_2_—P), 2.65–2.91 (m, 8H, 1′′‐CH_2_), 1.36–1.55 (m, 2H, CH_2_—CH_2_—P), 1.02–1.12 (m, 12H, CH_3_). ^13^C NMR (125 MHz, DMSO‐*d*_*6*_, mixture of diastereomers): *δ* = 172.80–173.25 (m, COO), 156.92 (C‐6), 148.81 and 148.82 (C‐4), 146.21 (C‐2), 138.83 (C‐8), 137.16–137.47 (m, C‐1′′), 129.54–129.65 (m, C‐2′′), 128.26–128.30 (m, C‐3′′), 126.59–126.67 (m, C‐4′′), 124.17 and 124.16 (C‐5), 82.58 and 82.53 (C‐1′), 70.99 (d, *J*(C—P) = 11.1 Hz, 1′‐O—CH_2_—CH_2_), 69.92 (C‐2′), 67.67 (d, *J*(C—P) = 135.2 Hz, O—CH_2_—P), 67.63 and 67.67 (1′‐O—CH_2_), 65.76 (CH_2_—CH_2_—P), 60.42–60.61 (CH_2_—CH_3_), 53.99–54.37 (m, NH—CH), 39.80 (1′′‐CH_2_), 29.37–30 (m, CH_2_—CH_2_—P), 14.04–14.12 (m, CH_3_). HRMS (ESI^+^): *m/z* [*M* + H]^+^ calcd. for C_56_H_73_O_14_N_8_P_2_ = 1143.47160, found: 1143.47247.

##### Chemical Synthesis: Tetra‐(L‐phenylalaninate ethyl ester) prodrug of (2‐(2‐(guanin‐9‐yl)‐2‐(2‐(phosphonatomethoxy)ethoxy)ethoxy)ethyl)phosphonic acid (19b)

Prepared according to general method D from **17b** (164 mg, 0.3 mmol), pyridine (8.5 mL), and TMSBr (0.3 mL, 378 mg, 2.5 mmol), then L‐phenylalanine ethyl ester hydrochloride (492 mg, 2.2 mmol), dry triethylamine (4 mL), dry pyridine (4 mL), Aldrithiol‐2 (864 mg, 3.3 mmol), and triphenylphosphine (725mg, 3.3 mmol) in pyridine (4 mL) to give **19b** (10 mg, 3%) as a yellowish solid. ^1^H NMR (500 MHz, DMSO‐*d*_*6*_, mixture of diastereomers): *δ* = 10.64 (bs, 1H, NHpur), 7.75–7.78 (m, 1H, H‐8), 7.08–7.26 (m, 20H, H‐2′′, H‐3′′, H‐4′′), 6.48 (bs, 2H, NH_2_), 5.49–5.54 (m, 1H, H‐1′), 4.41–4.50 (m, 2H, NH), 3.63–4.10 (m, 16H, CH_2_—CH_3_, 2 × NH, NH—CH, H‐2′), 3.16–3.51 (m, 8H, 1′‐O—CH_2_—CH_2_, CH_2_—CH_2_—P, O—CH_2_—P), 2.67–2.92 (m, 8H, 1′′‐CH_2_), 1.41–1.60 (m, 2H, CH_2_—CH_2_—P), 1.03–1.12 (m, 12H, CH_3_). ^13^C NMR (125 MHz, DMSO‐*d*_*6*_, mixture of diastereomers): *δ* = 172.78–173.20 (m, COO), 156.92 (C‐6), 153.93 (C‐2), 151.67 (C‐4), 137.13–137.44 (m, C‐1′′), 135.61 (C‐8), 129.51–129.60 (m, C‐2′′), 128.23–128.27 (m, C‐3′′), 126.58–126.64 (m, C‐4′′), 116.59 (C‐5), 81.83 (C‐1′), 70.87–70.97 (m, 1′‐O—CH_2_—CH_2_), 70.10 (C‐2′), 67.11–68.23 (m, O—CH_2_—P), 67.36 (1′‐O—CH_2_), 65.69 (CH_2_—CH_2_—P), 60.39–60.57 (m, CH_2_—CH_3_), 54.00–54.36 (m, NH—CH), 39.9 (1′′‐CH_2_), 29.40–30.30 (m, CH_2_—CH_2_—P), 14.01–14.08 (m, CH_3_). HRMS (ESI^+^): *m/z* [*M* + H]^+^ calcd. for C_56_H_75_O_14_N_9_P_2_ [*z* = 2] = 579.74489, found: 579.74531.

##### Chemical Synthesis: Sodium salt of ((2‐(2‐hydroxyethoxy)‐2‐(hypoxanthin‐9‐yl)ethoxy)methyl) phosphonic acid (20a)

Prepared according to general method C from **14a** (500 mg, 1.2 mmol), TMSBr (0.6 mL, 0.70 g, 4.5 mmol) in pyridine (5 mL) to give **20a** (234 mg, 52%) as a white solid. ^1^H NMR (500 MHz, D_2_O, ref: dioxane): *δ* = 8.37 (s, 1H, H‐8), 8.20 (s, 1H, H‐2), 6.02 (t, *J*(1′‐2′) = 5.2 Hz, 1H, H‐1′), 4.14 (dd, *J*(gem) = 11.1 Hz, *J*(2′a‐1′) = 5.4 Hz, 1H, H‐2′a), 4.08 (dd, *J*(gem) = 11.1 Hz, *J*(2′b‐1′) = 5.1 Hz, 1H, H‐2′b), 3.70–3.76 (m, 2H, CH_2_a—OH, 1′‐O—CH_2_a), 3.64 (m, 1H, CH_2_b—OH), 3.62 (d, *J*(CH_2_—P) = 8.5 Hz, 2H, CH_2_—P), 3.53 (ddd, *J*(gem) = 10.5 Hz, *J*(CH_2_—CH_2_) = 6.1 and 2.3 Hz, 1H, 1′‐O—CH_2_b). ^13^C NMR (125 MHz, D_2_O, ref: dioxane): *δ* = 159.35 (C‐6), 149.48 (C‐4), 146.67 (C‐2), 141.36 (C‐8), 124.18 (C‐5), 84.17 (C‐1′), 72.92 (d, *J*(C—P) = 10.7 Hz, C‐2′), 71.13 (1′‐O—CH_2_), 69.24 (d, *J*(C—P) = 152.9 Hz, CH_2_—P), 60.73 (CH_2_—OH). HRMS (ESI^−^): *m/z* [*M−*H]^−^ calcd. for C_10_H_14_O_7_N_4_P = 333.06056, found: 333.06042.

##### Chemical Synthesis: Sodium salt of ((2‐(guanin‐9‐yl)‐2‐(2‐hydroxyethoxy)ethoxy)methyl)phosphonic acid (20b)

Prepared according to general method C from **14b** (300 mg, 0.8 mmol), TMSBr (0.3 mL, 348 mg, 2.3 mmol) in pyridine (10 mL) to give **20b** (60 mg, 20%) as a white solid. ^1^H NMR (500.0 MHz, D_2_O, ref: dioxane): *δ* = 8.03 (s, 1H, H‐8), 5.82 (dd, *J*(1′‐2′a) = 5.5 Hz, *J*(1′‐2′b) = 5.1 Hz, 1H, H‐1′), 4.10 (dd, *J*(gem) = 11.0 Hz, *J*(2′a‐1′) = 5.5 Hz, 1H, H‐2′a), 4.03 (dd, *J*(gem) = 11.0 Hz, *J*(2′b‐1′) = 5.1 Hz, 1H, H‐2′b), 3.66–3.75 (m, 2H, 1′‐O—CH_2_a, CH_2_a—OH), 3.65 (d, *J*(CH_2_—P) = 8.5 Hz, 2H, CH_2_—P), 3.63 (m, 1H, CH_2_b—OH), 3.52 (ddd, *J*(gem) = 10.5 Hz, *J*(CH_2_—CH_2_) = 6.7 and 2.6 Hz, 1H, 1′‐O—CH_2_b). ^13^C NMR (125.7 MHz, D_2_O, ref: dioxane): *δ* = 159.65 (C‐6), 154.56 (C‐2), 152.32 (C‐4), 138.86 (C‐8), 116.58 (C‐5), 83.26 (C‐1′), 72.88 (d, *J*(C—P) = 10.7 Hz, C‐2′), 70.84 (1′‐O—CH_2_), 60.75 (CH_2_—OH), 68.93 (d, *J*(C—P) = 153.8 Hz, CH_2_—P). HRMS (ESI^−^): *m/z* [*M−*H]^−^ calcd. for C_10_H_15_O_7_N_5_P = 348.07146, found: 348.07104.

##### Chemical Synthesis: Sodium salt of (2‐(2‐(2‐hydroxyethoxy)‐2‐(hypoxanthin‐9‐yl)ethoxy)ethyl)phosphonic acid (20c)

Prepared according to general method C from **14c** (300 mg, 0.7 mmol), TMSBr (0.6 mL, 0.70 g, 4.5 mmol) in pyridine (12 mL) to give **20c** (118 mg, 35%) as a white solid. ^1^H NMR (500.0 MHz, D_2_O, ref: *t*BuOH): *δ* = 8.33 (s, 1H, H‐8), 8.21 (s, 1H, H‐2), 6.00 (t, *J*(1′‐2′) = 5.4 Hz, 1H, H‐1′), 4.03–4.10 (m, 2H, H‐2′), 3.52–3.81 (m, 6H, CH_2_—CH_2_—P, 1′‐O—CH_2_, CH_2_—OH), 1.82–1.94 (m, 2H, CH_2_—P). ^13^C NMR (125.7 MHz, D_2_O, ref: *t*BuOH): *δ* = 159.40 (C‐6), 149.56 (C‐4), 146.75 (C‐2), 141.23 (C‐8), 124.38 (C‐5), 84.37 (C‐1′), 71.25 (1′‐O—CH_2_), 70.84 (C‐2′), 68.13 (d, *J*(C—P) = 1.8 Hz, CH_2_—CH_2_—P), 60.86 (CH_2_—OH), 29.53 (d, *J*(C—P) = 129.3 Hz, CH_2_—P). HRMS (ESI^−^): *m/z* [*M−*H]^−^ calcd. for C_11_H_16_O_7_N_4_P = 347.07621, found: 347.07663.

##### Chemical Synthesis: Sodium salt of (2‐(2‐(2‐hydroxyethoxy)‐2‐(guanin‐9‐yl)ethoxy)ethyl)phosphonic acid (20d)

Prepared according to general method C from **14d** (310 mg, 0.7 mmol), TMSBr (0.6 mL, 0.70 g, 4.5 mmol) in pyridine (12 mL) to give **20d** (30 mg, 10%) as a white solid. ^1^H NMR (500.0 MHz, D_2_O, ref: dioxane): *δ* = 7.99 (s, 1H, H‐8), 5.81 (t, *J*(1′‐2′) = 5.6 Hz, 1H, H‐1′), 4.03 (dd, *J*(gem) = 11.2 Hz, *J*(2′a‐1′) = 5.7 Hz, H‐2′a), 3.97 (dd, *J*(gem) = 11.2 Hz, *J*(2′b‐1′) = 5.6 Hz, H‐2′b), 3.75–3.83 (m, 2H, CH_2_—CH_2_—P), 3.62–3.74 (m, 3H, CH_2_—OH, 1′‐O—CH_2_a), 3.53 (m, 1H, 1′‐O—CH_2_b), 1.81 (dm, *J*(CH_2_—P) = 18.0 Hz, 2H, CH_2_—P). ^13^C NMR (125.7 MHz, D_2_O, ref: dioxane): *δ* = 159.75 (C‐6), 154.61 (C‐2), 152.38 (C‐4), 138.65 (C‐8), 116.73 (C‐5), 83.39 (C‐1′), 70.88 (C‐2′), 70.56 (1′‐O—CH_2_), 69.06 (CH_2_—CH_2_—P), 60.80 (CH_2_—OH), 30.25 (d, *J*(C—P) = 125.8 Hz, CH_2_—P). HRMS (ESI^−^): *m/z* [*M−*H]^−^ calcd. for C_11_H_17_O_7_N_5_P = 362.08711, found: 362.08722.

##### Chemical Synthesis: Sodium salt of (2‐(2‐(2‐chlorohypoxanthin‐9‐yl)‐2‐(2‐hydroxyethoxy)ethoxy)ethyl)phosphonic acid (20e)

Prepared according to general method C from **14e** (400 mg, 0.9 mmol), TMSBr (0.6 mL, 0.70 g, 4.5 mmol) in pyridine (10 mL) to give **20e** (90 mg, 23%) as a white solid. ^1^H NMR (500.0 MHz, D_2_O, ref: dioxane): *δ* = 8.12 (s, 1H, H‐8), 5.83 (t, *J*(1′‐2′) = 5.5 Hz, 1H, H‐1′), 4.05 (dd, *J*(gem) = 11.1 Hz, *J*(2′a‐1′) = 5.7 Hz, 1H, H‐2′a), 4.00 (dd, *J*(gem) = 11.1 Hz, *J*(2′b‐1′) = 5.3 Hz, 1H, H‐2′b), 3.74–3.80 (m, 2H, CH_2_—CH_2_—P), 3.61–3.74 (m, 3H, CH_2_—OH, 1′‐O—CH_2_a), 3.49 (m, 1H, 1′‐O—CH_2_b), 1.82–1.90 (m, 2H, CH_2_—P). ^13^C NMR (125.7 MHz, D_2_O, ref: dioxane): *δ* = 167.95 (C‐6), 154.58 (C‐2), 151.22 (C‐4), 139.52 (C‐8), 122.92 (C‐5), 83.69 (C‐1′), 70.83 (1′‐O—CH_2_), 70.68 (C‐2′), 68.64 (CH_2_—CH_2_—P), 60.81 (CH_2_—OH), 29.26–30.41 (m, CH_2_—P). HRMS (ESI^−^): *m/z* [*M−*H]^−^ calcd. for C_11_H_15_O_7_N_4_ClP = 381.03724, found: 381.03677.

##### Chemical Synthesis: Sodium salt of (2‐(2‐(2‐hydroxyethoxy)‐2‐(4‐oxo‐4,5‐dihydro‐1*H*‐pyrazolo[3,4‐d]pyrimidin‐1‐yl)ethoxy)ethyl)phosphonic acid (20f)

Prepared according to general method C from **14f** (282 mg, 0.7 mmol), TMSBr (0.5 mL, 580 mg, 3.8 mmol) in pyridine (13 mL) to give **20f** (142 mg, 52%) as a white solid. ^1^H NMR (500.0 MHz, D_2_O, ref: *t*BuOH): *δ* = 8.29 (s, 1H, H‐7), 8.23 (s, 1H, H‐2), 6.14 (m, 1H, H‐1′), 4.15 (dd, *J*(gem) = 11.0 Hz, *J*(2′a‐1′) = 5.7 Hz, 1H, H‐2′a), 4.07 (dd, *J*(gem) = 11.0 Hz, *J*(2′b‐1′) = 7.0 Hz, 1H, H‐2′b), 3.65–3.82 (m, 4H, CH_2_—CH_2_—P, CH_2_a—OH, 1′‐O—CH_2_a), 3.61 (m, 1H, CH_2_b—OH), 3.41 (m, 1H, 1′‐O—CH_2_b), 1.76–1.90 (m, 2H, CH_2_—P). ^13^C NMR (125.7 MHz, D_2_O, ref: *t*BuOH): *δ* = 160.77 (C‐6), 154.38 (C‐4), 149.50 (C‐7), 137.50 (C‐2), 106.92 (C‐5), 84.62 (C‐1′), 70.76 (1′‐O‐CH_2_), 70.14 (C‐2′), 68.07 (d, *J*(C‐P) = 2.1 Hz, CH_2_—CH_2_—P), 60.81 (CH_2_—OH), 29.63 (d, *J*(C—P) = 128.8 Hz, CH_2_—P). HRMS (ESI^−^): *m/z* [*M−*H]^−^ calcd. for C_11_H_16_O_7_N_4_P = 347.07621, found: 347.07571.

##### Chemical Synthesis: Sodium salt of ((3‐(3‐hydroxypropoxy)‐3‐(hypoxanthin‐9‐yl)propoxy)methyl)phosphonic acid (20g)

Prepared according to general Method C from **14g** (270 mg, 0.6 mmol), TMSBr (0.4 mL, 464 mg, 3.0 mmol) in pyridine (12 mL) to give **20g** (108 mg, 44%) as a white solid. ^1^H NMR (500.0 MHz, D_2_O, ref: *t*BuOH): *δ* = 8.31 (s, 1H, H‐8), 8.19 (s, 1H, H‐2), 5.95 (t, *J*(1′‐2′) = 6.8 Hz, 1H, H‐1′), 3.44–3.73 (m, 8H, CH_2_—P, CH_2_‐OH, H‐3′, 1′‐O—CH_2_), 2.38 and 2.55 (2 × m, 2 × 1H, H‐2′), 1.70–1.83 (m, 2H, 1′‐O—CH_2_—CH_2_). ^13^C NMR (125.7 MHz, D_2_O, ref: *t*BuOH): *δ* = 159.48 (C‐6), 149.37 (C‐4), 146.54 (C‐2), 141.30 (C‐8), 124.61 (C‐5), 84.59 (C‐1′), 68.65 (d, *J*(C—P) = 11.8 Hz, C‐3′), 68.34 (d, *J*(C—P) = 154.5 Hz, CH_2_—P), 66.77 (1′‐O—CH_2_), 59.03 (CH_2_—OH), 35.17 (C‐2′), 31.65 (1′‐O—CH_2_—CH_2_). HRMS (ESI^−^): *m/z* [*M−*H]^−^ calcd. for C_12_H_18_O_7_N_4_P = 361.09186, found: 361.09225.

##### Chemical Synthesis: Sodium salt of ((3‐(guanin‐9‐yl)‐3‐(3‐hydroxypropoxy)propoxy)methyl)phosphonic acid (20 h)

Prepared according to general method C from **14h** (200 mg, 0.4 mmol), TMSBr (0.3 mL, 348 mg, 2.3 mmol) in pyridine (9 mL) to give **20 h** (82 mg, 45%) as a white solid. ^1^H NMR (500.0 MHz, D_2_O, ref: *t*BuOH): *δ* = 7.90 (s, 1H, H‐8), 5.75 (t, (1′‐2′) = 6.8 Hz, H‐1′), 3.41–3.68 (m, 8H, 1′‐O—CH_2_, H‐3′, CH_2_—OH, CH_2_—P), 2.31 and 2.47 (2 × m, 2 × 1H, H‐2′), 1.69–1.81 (m, 2H, 1′‐O—CH_2_—CH_2_). ^13^C NMR (125.7 MHz, D_2_O, ref: *t*BuOH): *δ* = 167.26 (C‐6), 160.62 (C‐2), 152.22 (C‐4), 137.31 (C‐8), 118.06 (C‐5), 83.12 (C‐1′), 69.65 (d, *J*(C—P) = 150.6 Hz, CH_2_—P), 68.65 (d, *J*(C‐P) = 11.3 Hz, C‐3′), 66.42 (1′‐O—CH_2_), 59.18 (CH_2_—OH), 35.18 (C‐2′), 31.72 (1′‐O—CH_2_—CH_2_). HRMS (ESI^−^): *m/z* [*M−*H]^−^ calcd. for C_12_H_19_O_7_N_5_P = 376.10276, found: 376.10229.

##### Chemical Synthesis: Sodium salt of ((3‐(3‐hydroxypropoxy)‐3‐(4‐oxo‐4,5‐dihydro‐1H‐pyrazolo[3,4‐d]pyrimidin‐1‐yl)propoxy)methyl)phosphonic acid (20i)

Prepared according to general method C from **14i** (150 mg, 0.3 mmol), TMSBr (0.2 mL, 232 mg, 1.5 mmol) in pyridine (7 mL) to give **20i** (90 mg, 67%) as a white solid. ^1^H NMR (500.0 MHz, D_2_O, ref: *t*BuOH): *δ* = 8.28 (s, 1H, H‐7), 8.22 (s, 1H, H‐2), 5.96 (t, *J*(1′‐2′) = 6.7 Hz, 1H, H‐1′), 3.43–3.52 (m, 3H, H‐3′a, 1′‐O—CH_2_a, CH_2_a—OH), 3.38 (m, 1H, CH_2_b—OH), 3.30 (d, *J*(CH_2_—P) = 8.7 Hz, 2H, CH_2_—P), 3.17–3.26 (m, 2H, H‐3′b, 1′‐O—CH_2_b), 2.23 and 2.38 (2 × m, 2 × 1H, H‐2′), 1.52–1.63 (m, 2H, 1′‐O—CH_2_—CH_2_). ^13^C NMR (125.7 MHz, D_2_O, ref: *t*BuOH): *δ* = 160.98 (C‐6), 153.79 (C‐4), 149.42 (C‐2), 137.17 (C‐7), 106.72 (C‐5), 84.63 (C‐1′), 68.76 (d, *J*(C—P) = 153.2 Hz, CH_2_—P), 68.60 (d, *J*(C—P) = 11.3 Hz, C‐3′), 66.45 (1′‐O—CH_2_), 59.06 (CH_2_—OH), 34.32 (C‐2′), 31.64 (1′‐O—CH_2_—CH_2_). HRMS (ESI^−^): *m/z* [*M−*H]^−^ calcd. for C_12_H_18_O_7_N_4_P = 361.09186, found: 361.09186.

##### Chemical Synthesis: Bis‐(L‐phenylalaninate ethyl ester) prodrug of ((2‐(2‐hydroxyethoxy)‐2‐(hypoxanthin‐9‐yl)ethoxy)methyl)phosphonic acid (21a)

Prepared according to general method D from **14a** (1.2 g, 2.9 mmol), TMSBr (1.5 mL, 1.8 g, 12 mmol), and pyridine (15 mL), then L‐phenylalanine ethyl ester hydrochloride (2.6 g, 12 mmol), Aldrithiol‐2 (3.8 g, 17 mmol), triphenylphosphine (4.5 g, 17 mmol), dry triethylamine (7.5 mL), dry pyridine (7.5 mL) to give **21a** (400 mg, 20%) as a white solid. ^1^H NMR (500 MHz, DMSO‐*d*_*6*_, mixture of diastereomers): *δ* = 12.17 (bs, 1H, NHpur), 8.23 (2 × s, 1H, H‐8), 8.05 and 8.03 (s, 1H, H‐2), 7.06–7.27 (m, 10H, H‐2′′, H‐3′′, H‐4′′), 5.78–5.81 (m, 1H, H‐1′), 4.68 (bs, 1H, OH), 4.39–4.37 and 4.08–4.14 (2 × m, 2H, NH), 3.81–4.02 (m, 8H, H‐2′, CH_2_—CH_3_, NH—CH), 3.27–3.54 (m, 6H, CH_2_—P, CH_2_—OH, 1′‐O—CH_2_), 2.69–2.87 (m, 4H, 1′′—CH_2_), 1.03–1.11 (m, 6H, CH_3_). ^13^C NMR (125 MHz, DMSO‐*d*_*6*_, mixture of diastereomers): *δ* = 172.62–172.84 (m, COO), 156.80 (C‐6), 148.73 (C‐4), 146.09 (C‐2), 138.75 (C‐8), 137.25, and 137.08 (C‐1′′), 129.51 (C‐2′′), 128.22 (C‐3′′), 126.60 and 126.57 (C‐4′′), 124.09 (C‐5), 82.44 and 82.39 (C‐1′), 71.97–72.12 (m, C‐2′), 70.64 (1′‐O—CH_2_), 68.07 (d, *J*(C—P) = 134.4 Hz, CH_2_—P), 60.52 and 60.47 (CH_2_—CH_3_), 59.82 (CH_2_—OH), 54.00 (NH—CH), 39.9 (1′′—CH_2_), 14.04 and 13.98 (CH_3_). HRMS (ESI^+^): *m/z* [*M* + H]^+^ calcd. for C_32_H_41_O_9_N_6_NaP = 707.25649, found: 707.25616.

##### Chemical Synthesis: Bis‐(L‐phenylalaninate ethyl ester) prodrug of ((2‐(guanin‐9‐yl)‐2‐(2‐hydroxyethoxy)ethoxy)methyl)phosphonic acid (21b)

Prepared according to general method D from **14b** (830 mg, 1.9 mmol), pyridine (20 mL), and TMSBr (2 mL, 2.3 g, 15.3 mmol), then L‐phenylalanine ethyl ester hydrochloride (1.8 g, 7.6 mmol), Aldrithiol‐2 (3.0 g, 11.4 mmol), triphenylphosphine (2.5 g, 11.4 mmol), dry triethylamine (8 mL), and dry pyridine (8 mL) to give **21b** (340 mg, 26%) as a brownish viscous oil. ^1^H NMR (500 MHz, DMSO‐*d*_*6*_, mixture of diastereomers): *δ* = 10.62 (bs, 1H, NHpur), 7.82 and 7.81 (s, 1H, H‐8), 7.07–7.27 (m, 10H, H‐2′′, H‐3′′, H‐4′′), 6.47 (bs, 2H, NH_2_), 5.53–5.59 (m, 1H, H‐1′), 4.67 (m, 1H, OH), 4.43 and 4.12 (2 × m, 2H, NH), 3.78–4.03 (m, 8H, CH_2_—CH_3_, NH—CH, H‐2′), 3.27–3.47 (m, 6H, CH_2_—OH, CH_2_—P, 1′‐O—CH_2_), 2.71–2.90 (m, 4H, 1′′‐CH_2_), 1.04–1.11 (m, 6H, CH_3_). ^13^C NMR (125 MHz, DMSO‐*d*_*6*_, mixture of diastereomers): *δ* = 172.71–172.95 (m, COO), 156.93 (C‐6), 153.91 (C‐2), 151.62 (C‐4), 137.33, and 137.14 (C‐1′′), 135.78 and 135.76 (C‐8), 129.59 (C‐2′′), 128.29 (C‐3′′), 126.67, and 126.64 (C‐4′′), 116.60 and 116.58 (C‐5), 81.77 and 81.72 (C‐1′), 72.15–72.24 (m, C‐2′), 70.40 and 70.37 (1′‐O—CH_2_), 67–57 (m, CH_2_—P), 60.59 and 60.54 (CH_2_—CH_3_), 59.86 (CH_2_—OH), 54.06 (NH—CH), 39.9 (1′′‐CH_2_), 14.10, 14.05, and 14.04 (CH_3_). HRMS (ESI^+^): *m/z* [*M* + H]^+^ calcd. for C_32_H_43_O_9_N_7_P = 700.28544, found: 700.28563.

##### Chemical Synthesis: Bis‐(L‐phenylalaninate ethyl ester) prodrug of (2‐(2‐(2‐hydroxyethoxy)‐2‐(hypoxanthin‐9‐yl)ethoxy)ethyl) phosphonic acid (21c)

Prepared according to general method D from **14c** (1.0 g, 2.5 mmol), pyridine (20 mL), and TMSBr (1.3 mL, 1.5 g, 9.9 mmol), then L‐phenylalanine ethyl ester hydrochloride (2.3 g, 9.9 mmol), Aldrithiol‐2 (3.9 g, 14.9 mmol), triphenylphosphine (3.3 g, 14.9 mmol), dry triethylamine (9 mL), and dry pyridine (9 mL) to give **21c** (362 mg, 21%) as a white solid. ^1^H NMR (500 MHz, DMSO‐*d*_*6*_, mixture of diastereomers): *δ* = 12.37 (bs, 1H, NHpur), 8.21 (s, 1H, H‐8), 8.06 (s, 1H, H‐2), 7.06–7.27 (m, 10H, H‐2′′, H‐3′′, H‐4′′), 5.77 (m, 1H, H‐1′), 4.67 (m, 1H, OH), 4.44 (m, 1H, NH), 3.89–4.04 (m, 6H, CH_2_—CH_3_, NH—CH), 3.75–3.86 (m, 3H, NH—CH, H‐2′), 3.29–3.53 (m, 6H, 1′‐O—CH_2_, CH_2_—OH, CH_2_—CH_2_—P), 2.78–2.89 and 2.70 (2 × m, 4H, 1′′‐CH_2_), 1.40–1.63 (m, 2H, CH_2_—P), 1.03–1.13 (m, 6H, CH_3_). ^13^C NMR (125 MHz, DMSO‐*d*_*6*_, mixture of diastereomers): *δ* = 172.95–173.18 (m, COO), 156.78 (C‐6), 148.76 (C‐4), 146.05 (C‐2), 138.82 (C‐8), 137.43 and 137.39 (C‐1′′), 129.56 and 129.52 (C‐2′′), 128.27 and 128.23 (C‐3′′), 126.64 and 126.56 (C‐4′′), 124.09 (C‐5), 82.71 and 82.68 (C‐1′), 70.65 (1′‐O—CH_2_), 70.03 (C‐2′), 65.72 (CH_2_—CH_2_—P), 60.49 and 60.39 (CH_2_—CH_3_), 59.84 (CH_2_—OH), 54.34 and 53.98 (NH—CH), 40.07 (1′′‐CH_2_), 29.46–30.23 (m, CH_2_—P), 14.08 and 14.01 (CH_3_). HRMS (ESI^+^): *m/z* [*M* + H]^+^ calcd. for C_33_H_44_O_9_N_6_P = 699.29019, found: 699.29058.

##### Chemical Synthesis: Bis‐(L‐phenylalaninate ethyl ester) prodrug of (2‐(2‐(guanin‐9‐yl)‐2‐(2‐hydroxyethoxy)ethoxy)ethyl) phosphonic acid (21d)

Prepared according to general method D from **14d** (40 mg, 0.10 mmol), pyridine (3 mL), and TMSBr (0.08 mL, 90 mg, 0.6 mmol), then L‐phenylalanine ethyl ester hydrochloride (88 mg, 0.4 mmol), Aldrithiol‐2 (150 mg, 0.6 mmol), triphenylphosphine (126 mg, 0.6 mmol), dry triethylamine (1 mL), and dry pyridine (1 mL) to give **21d** (33 mg, 49%) as a white solid. ^1^H NMR (500 MHz, DMSO‐*d*_*6*_, mixture of diastereomers): *δ* = 10.63 (bs, 1H, NHpur), 7.77–7.79 (m, 1H, H‐8), 7.06–7.28 (m, 10H, H‐2′′, 3′′, 4′′), 6.48 (bs, 2H, NH_2_), 5.45–5.55 (m, 1H, H‐1′), 4.60–4.68 (m, 1H, OH), 4.45–4.53 (m, 1H, NH), 3.90–4.10 (m, 6H, NH, CH_2_—CH_3_, NH—CH), 3.67–3.85 (m, 3H, H‐2′, NH—CH), 3.25–3.47 (m, 6H, 1′‐O—CH_2_, CH_2_—OH, CH_2_—CH_2_—P), 2.65–2.89 (m, 4H, 1′′‐CH_2_), 1.41–1.63 (m, 2H, CH_2_P), 1.03–1.13 (m, 6H, CH_3_). ^13^C NMR (125 MHz, DMSO‐*d*_*6*_, mixture of diastereomers): *δ* = 172.98–173.24 (m, COO), 156.93 (C‐6), 153.90 and 153.86 (C‐2), 151.57–151.66 (m, C‐4), 137.30–137.46 (m, C‐1′′), 135.69 (C‐8), 129.50–129.59 (C‐2′′), 128.30 and 128.27 (C‐3′′), 126.60–126.70 (m, C‐4′′), 116.55–116.64 (m, C‐5), 81.93–82.00 (m, C‐1′), 70.22–72.86 (m, C‐2′, 1′‐O—CH_2_), 65.67 (CH_2_—CH_2_—P), 59.86–60.58 (m, CH_2_—OH, CH_2_—CH_3_), 53.88–54.39 (m, NH—CH), 29.86 (d, *J*(C—P) = 110.8 Hz, CH_2_—P), 14.12 and 14.05 (CH_3_). HRMS (ESI^+^): *m/z* [*M* + H]^+^ calcd. for C_33_H_45_O_9_N_7_P = 714.30109, found: 714.30135.

##### Chemical Synthesis: Bis‐(L‐phenylalaninate ethyl ester) prodrug of ((3‐(3‐hydroxypropoxy)‐3‐(hypoxanthin‐9‐yl)propoxy)methyl) phosphonic acid (21e)

Prepared according to general method D from **14g** (370 mg, 0.8 mmol), pyridine (12 mL), and TMSBr (0.6 mL, 0.70 g, 4.5 mmol), then L‐phenylalanine ethyl ester hydrochloride (760 mg, 3.3 mmol), Aldrithiol‐2 (1.3 g, 5.0 mmol), triphenylphosphine (1.1 g, 5.0 mmol), dry triethylamine (7.5 mL), and dry pyridine (7.5 mL) to give **21e** (135 mg, 23%) as a white solid. ^1^H NMR (500 MHz, DMSO‐*d*_*6*_, mixture of diastereomers): *δ* = 12.38 (bs, 1H, NHpur), 8.23 (s, 1H, H‐8), 8.04 and 7.97 (2 × s, 1H, H‐2), 7.10–7.27 (m, 10H, H‐2′′, H‐3′′, H‐4′′), 5.72 (m, 1H, H‐1′), 4.40–4.56 (m, 2H, OH, NH), 4.09–4.19 (m, 1H, NH), 3.86–4.06 (m, 6H, NH—CH, CH_2_—CH_3_), 3.05–3.49 (m, 8H, H‐3′, CH_2_—P, 1′‐O—CH_2_, CH_2_—OH), 2.73–2.93 (m, 4H, 1′′‐CH_2_), 2.17 and 2.35 (2 × m, 2H, H‐2′), 1.54–1.60 (m, 2H, 1′‐O—CH_2_—CH_2_), 1.04–1.13 (m, 6H, CH_3_). ^13^C NMR (125 MHz, DMSO‐*d*_*6*_, mixture of diastereomers): *δ* = 172.84–173.15 (m, COO), 156.88 and 156.87 (C‐6), 148.58 (C‐4), 146.15 and 145.99 (C‐2), 138.65 and 138.54 (C‐8), 137.26–137.40 (m, C‐1′′), 129.63–129.66 (m, C‐2′′), 128.26–128.30 (m, C‐3′′), 126.64–136.70 (m, C‐4′′), 124.22 and 124.16 (C‐5), 82.00 (C‐1′), 66.99–68.15 (m, C‐3′ and CH_2_—P), 65.79 and 65.78 (1′‐O—CH_2_), 60.52–60.63 (m, CH_2_—CH_3_), 57.54 (CH_2_—OH), 57.53 (CH_2_—OH), 54.06–54.22 (m, NH—CH), 39.80 (1′′‐CH_2_), 34.99 and 34.97 (C‐2′), 32.36 and 32.35(1′‐O—CH_2_—CH_2_), 14.06–14.14 (m, CH_3_). HRMS (ESI^+^): *m/z* [*M* + H]^+^ calcd. for C_34_H_46_O_9_N_6_P = 713.30584, found: 713.30625.

##### Chemical Synthesis: Bis‐(L‐phenylalaninate ethyl ester) prodrug of ((3‐(3‐hydroxypropoxy)‐3‐(guanin‐9‐yl)propoxy)methyl) phosphonic acid (21f)

Prepared according to general method D from **14h** (390 mg, 0.9 mmol), pyridine (17 mL), and TMSBr (0.6 mL, 0.70 g, 4.5 mmol), then L‐phenylalanine ethyl ester hydrochloride (758 mg, 3.4 mmol), Aldrithiol‐2 (1.3 g, 5.1 mmol), triphenylphosphine (1.1 g, 5.1 mmol), dry triethylamine (8 mL), and dry pyridine (8 mL) to give **21f** (158 mg, 26%) as a white solid. ^1^H NMR (500 MHz, DMSO‐*d*_*6*_, mixture of diastereomers): *δ* = 10.74 (bs, 1H, NHpur), 7.80 (s, 1H, H‐8), 7.11–7.27 (m, 10H, H‐2′′, H‐3′′, H‐4′′), 6.51 (bs, 2H, NH_2_), 5.47 (m, 1H, H‐1′), 4.41–4.52 (m, 2H, NH, OH), 4.07–4.15 (m, 1H, NH), 3.86–4.06 (m, 6H, NH—CH, CH_2_—CH_3_), 3.05–3.41 (m, 8H, H‐3′, CH_2_—P, 1′‐O—CH_2_, CH_2_—OH), 2.74–2.93 (m, 4H, 1′′‐CH_2_), 2.28 and 2.07 (2 × m, 2H, H‐2′), 1.54–1.61 (m, 2H, 1′‐O—CH_2_—CH_2_), 1.04–1.13 (m, 6H, CH_3_). ^13^C NMR (125 MHz, DMSO‐*d*_*6*_, mixture of diastereomers): *δ* = 172.86–173.11 (m, COO), 157.22 (C‐6), 154.05 (C‐2), 151.48 (C‐4), 137.19–137.41 (m, C‐1′′), 135.56 (C‐8), 129.66 and 129.64 (C‐2′′), 128.27–128.30 (m, C‐3′′), 126.64–126.70 (m, C‐4′′), 116.75 (C‐5), 81.12 (C‐1′), 67.08–68.26 (m, C‐3′, CH_2_—P), 65.53 (1′‐O—CH_2_), 60.53–60.67 (m, CH_2_—CH_3_), 57.63 (CH_2_—OH), 54.05–54.16 (m, NH—CH), 39.90 (1′′‐CH_2_), 34.87 (C‐2′), 32.43 (1′‐O—CH_2_—CH_2_), 14.06–14.13 (m, CH_3_). HRMS (ESI^+^): *m/z* [*M* + H]^+^ calcd. for C_34_H_47_O_9_N_7_P = 728.31674, found: 728.31715.

##### Chemical Synthesis: Bis‐(L‐phenylalaninate ethyl ester) prodrug of ((3‐(3‐hydroxypropoxy)‐3‐(4‐oxo‐4,5‐dihydro‐1H‐pyrazolo[3,4‐d]pyrimidin‐1‐yl)propoxy)methyl) phosphonic acid (21g)

Prepared according to general method D from **14i** (210 mg, 0.5 mmol), pyridine (10 mL), and TMSBr (0.3 mL, 360 mg, 2.4 mmol), then L‐phenylalanine ethyl ester hydrochloride (421 mg, 1.9 mmol), Aldrithiol‐2 (740 mg, 2.9 mmol), triphenylphosphine (622 mg, 2.9 mmol), dry triethylamine (4.5 mL), and dry pyridine (4.5 mL) to give **21g** (110 mg, 33%) as a white solid. ^1^H NMR (500 MHz, DMSO‐*d*_*6*_, mixture of diastereomers): *δ* = 12.31 (bs, 1H, NHpur), 8.16 and 8.15 (2 × s, 1H, H‐2), 8.09 and 8.03 (2 × s, 1H, H‐2), 7.10–7.27 (m, 10H, H‐2′′, H‐3′′, H‐4′′), 5.90 (H‐1′), 4.50 (m, 1H, NH), 4.41 (m, 1H, OH), 4.12 (m, 1H, NH), 3.86–4.06 (m, 6H, NH—CH, CH_2_—CH_3_), 2.99–3.47 (m, 8H, H‐3′, CH_2_—P, 1′‐O—CH_2_—CH_2_, CH_2_—OH), 2.72–2.92 (m, 4H, 1′′‐CH_2_), 2.34 and 2.17 (2 × m, 2H, H‐2′), 1.51–1.56 (m, 2H, 1′‐O—CH_2_—CH_2_), 1.04–1.13 (m, 6H, CH_3_). ^13^C NMR (125 MHz, DMSO‐*d*_*6*_, mixture of diastereomers): *δ* = 172.84–173.14 (m, COO), 157.84 (C‐6), 153.40 and 153.37 (C‐4), 149.02 and 148.87 (C‐2), 137.27–137.40 (m, C‐1′′), 135.71 and 135.61 (C‐7), 129.67–129.69 (m, C‐2′′), 128.32 and 128.30 (C‐3′′), 126.67–126.73 (m, C‐4′′), 106.05 and 106.01 (C‐5), 82.77 and 82.72 (C‐1′), 67.05–68.23 (m, C‐3′, CH_2_—P), 65.46 (1′‐O—CH_2_), 60.53–60.65 (m, CH_2_—CH_3_), 57.65 and 57.64 (CH_2_—OH), 54.08–54.21 (m, NH—CH), 39.90 (1′′‐CH_2_), 33.99 (C‐2′), 32.43 and 32.42 (1′‐O—CH_2_—CH_2_), 14.08–14.16 (m, CH_3_). HRMS (ESI^+^): *m/z* [*M* + H]^+^ calcd. for C_34_H_46_O_9_N_6_P = 713.30584, found: 713.30625.

##### Chemical Synthesis: Diethyl (2‐(2‐(2‐acetamido‐6‐chloro‐9*H*‐purin‐9‐yl)‐2‐ethoxyethoxy)ethyl)phosphonate (23)

Prepared according to general method A from **9** (210 mg, 1.0 mmol), phosphonate **22** (300 mg, 1.0 mmol), acetic anhydride (0.95 mL, 102 mg, 1.0 mmol), and TMSOTf (0.27 mL, 333 mg, 1.5 mmol, 3 eq) in dry MeCN (8 mL) to give **23** (430 mg, 90%) as a viscous oil. ^1^H NMR (500 MHz, DMSO‐*d*_*6*_): *δ* = 10.87 (bs, 1H, NH), 8.71 (s, 1H, H‐8), 5.85 (dd, *J*(1′‐2′a) = 6.8 Hz, *J*(1′‐2′b) = 5.1 Hz, 1H, H‐1′), 4.11 (dd, *J*(gem) = 10.7 Hz, *J*(gem) = 6.7 Hz, 1H, H‐2′a), 3.96 (dd, *J*(gem) = 10.7 Hz, *J*(gem) = 5.0 Hz, 1H, H‐2′b), 3.84–3.91 (m, 4H, P–O—CH_2_—CH_3_), 3.51–3.69 (m, 3H, 1′‐O—CH_2_a, CH_2_—CH_2_—P), 3.40 (m, 1′‐O—CH_2_b), 2.20 (s, 3H, CH_3_—CON), 1.95–2.02 (m, 2H, CH_2_—P), 1.14 (2 × t, 2 × 3H, *J*(CH_3_—CH_2_) = 7.1 Hz, P—O—CH_2_—CH_3_), 1.07 (t, 3H, *J*(CH_3_—CH_2_) = 7.0 Hz, 1′‐O—CH_2_—CH_3_). ^13^C NMR (125 MHz, DMSO‐*d*_*6*_): *δ* = 169.00 (CON), 153.21 (C‐4), 152.40 (C‐2), 149.38 (C‐6), 145.14 (C‐8), 127.36 (C‐5), 82.58 (C‐1′), 69.54 (C‐2′), 65.15 (d, *J*(C—P) = 1.7 Hz, CH_2_—CH_2_—P), 64.62 (1′‐O—CH_2_), 61.08–61.15 (m, P–O‐CH_2_‐CH_3_), 25.94 (d, *J*(C—P) = 137.0 Hz, CH_2_—P), 24.84 (CH_3_—CON), 16.36–16.42 (m, P—O—CH_2_—CH_3_), 14.86 (1′‐O—CH_2_—CH_3_). HRMS (ESI^+^): *m/z* [*M* + H]^+^ calcd. for C_17_H_27_O_6_N_5_ClNaP = 486.12797, found: 486.12881.

##### Chemical Synthesis: Diethyl (2‐(2‐ethoxy‐2‐(guanin‐9‐yl)ethoxy)ethyl)phosphonate (24)

Prepared according to general method B from **23** (480 mg, 1.0 mmol), DABCO (140 mg, 1.2 mmol), K_2_CO_3_ (430 g, 3.1 mmol) under reflux in water (7 mL) for 1 h to give **24** (160 mg, 38%) as a brownish viscous oil. ^1^H NMR (500 MHz, DMSO‐*d*_*6*_): *δ* = 10.64 (bs, 1H, NH), 7.84 (s, 1H, H‐8), 6.49 (bs, 2H, NH_2_), 5.55 (dd, *J*(1′‐2′) = 6.2 Hz, *J*(1′‐2′) = 5.5 Hz, 1H, H‐1′), 3.83–3.96 (m, 6H, H‐2′ and P—O—CH_2_—CH_3_), 3.55–3.67 (m, 2H, CH_2_—CH_2_—P), 3.30–3.50 (m, 2H, 1′‐O—CH_2_—CH_3_), 1.94–2.07 (m, 2H, CH_2_—P), 1.04–1.20 (m, 9H, P—O—CH_2_—CH_3_, 1′‐O—CH_2_—CH_3_). ^13^C NMR (125 MHz, DMSO‐*d*_*6*_): *δ* = 156.95 (C‐6), 153.92 (C‐2), 151.72 (C‐4), 135.75 (C‐8), 116.51 (C‐5), 81.41 (C‐1′), 70.35 (C‐2′), 65.08 (d, *J*(C—P) = 1.3 Hz, CH_2_—CH_2_—P), 63.98 (1′‐O—CH_2_—CH_3_), 61.12–61.17 (m, P—O—CH_2_—CH_3_), 26.04 (d, *J*(C—P) = 136.8 Hz, CH_2_—P), 16.39 (d, *J*(C—P) = 5.9 Hz, P—O—CH_2_—CH_3_), 14.90 (1′‐O—CH_2_—CH_3_). HRMS (ESI^+^): *m/z* [*M* + H]^+^ calcd. for C_15_H_27_O_6_N_5_P = 404.16935, found: 404.16950.

##### Chemical Synthesis: Sodium salt of (2‐(2‐(guanin‐9‐yl)‐2‐ethoxyethoxy)ethyl)phosphonic acid (25)

Prepared according to general method C from **24** (130 mg, 0.3 mmol) and TMSBr (0.2 mL, 247 mg, 1.6 mmol) in pyridine (6 mL) to give **25** (41 mg, 37%) as a white solid. ^1^H NMR (500.0 MHz, D_2_O, ref: *t*BuOH): *δ* = 7.97 (s, 1H, H‐8), 5.77 (t, *J*(1′‐2′) =5.7 Hz, 1H, H‐1′), 3.99 (dd, *J*(gem) = 11.0 Hz, *J*(2′a‐1′) = 5.8 Hz, 1H, H‐2′a); 3.94 (dd, *J*(gem) = 11.0 Hz, *J*(2′b‐1′) = 5.5 Hz, 1H, H‐2′b); 3.72–3.82 (m, 2H, CH_2_—CH_2_—P); 3.64 and 3.53 (2 × dq, *J*(gem) = 9.6 Hz, *J*(CH_2_—CH_3_) = 7.1 Hz, 2 × 1H, CH_2_—CH_3_), 1.81–1.90 (m, 2H, CH_2_—P), 1.15 (t, *J*(CH_3_—CH_2_) = 7.1 Hz, 3H, CH_3_). ^13^C NMR (125.7 MHz, D_2_O, ref: *t*BuOH): *δ* = 159.81 (C‐6), 154.65 (C‐2), 152.47 (C‐4), 138.73 (C‐8), 116.78 (C‐5), 83.11 (C‐1′), 70.77 (C‐2′), 68.62 (d, *J*(C—P) = 2.6 Hz, CH_2_—CH_2_—P), 66.11 (CH_2_—CH_3_), 29.98 (d, *J*(C—P) = 127.3 Hz, CH_2—_P), 14.59 (CH_3_). HRMS (ESI^−^): *m/z* [*M−*H]^−^ calcd. for C_11_H_17_O_6_N_5_P = 346.09219, found: 346.09188.

##### Determination of K_i_ Values

The human and *Pf* enzymes were purified as described previously.^[^ ^7^ ^,^ ^26^ ^]^ All assays were performed in 0.1 M Tris‐HCl, 10 mM MgCl_2_, pH 7.4. The *K*_*i*_ values were calculated by Hanes’ plots at a fixed concentration of guanine (60 μM) and at variable concentrations of *P*Rib‐*PP* (14−1000 μM), depending on the *K*_*m*(app)_, in the presence of the inhibitor. The *K*_*i*_ was calculated using the equation, *v*_o _= V_max._[S]_o_/[S]_o _+ *K*_*m*(app)_ and *K*_*m*(app) _= *K*_*m*_[1 + [I]/*K*_*i*_].

##### Docking

Ligand preparation included protonation, energy minimization was performed in Open Bable 3.1.1,^[^ ^30^ ^]^ where energy minimization was performed using the MMFF94 force field. Protein preparation was performed using GOLD 2024.2.0.401,^[^ ^31^ ^]^ which included adding hydrogen atoms to the protein, removing water molecules and the removal of the ligands. Docking was conducted using the same version of GOLD, where the binding pocket was defined within a 5 Å radius of the original small molecule ligand (i.e., the original ANP). Docking was performed three different times and conformations with the highest ten ChemPLP scores in GOLD were analyzed

##### In Vitro Antimalarial Activity of the C1^′^‐Branched ANP Prodrugs

The IC_50_ values were determined as previously described.^[^ ^32^ ^]^ *P. falciparum* D6 (Sierra‐Leone) laboratory line, sensitive to most antimalarial drugs, and W2 (Indochina) line, resistant to chloroquine and pyrimethamine, were maintained as previously described^[^ ^33^ ^]^ in medium consisted of 10.4 g L^−1^ RPMI‐1640‐LPLF powder (Gibco BRL), 5.97 g L^−1^ HEPES buffer (MP Biomedicals, USA), 2.0 g L^−1^ d‐glucose (BDH chemicals, Australia), 0.05 g L^−1^ hypoxanthine (Sigma, USA) and 40 mg L^−1^ gentamycin (Pfizer, Australia), supplemented with 0.21% NaHCO_3_ and 10% human plasma prior to use. Red blood cells O (Rh+) type were obtained from the Australian Red Cross Blood Service and added to 4% haematocrit. Cultures were routinely synchronized using D‐sorbitol.^[^ ^34^ ^]^ To evaluate the antimalarial activity of the ANPs, the ^3^H‐hypoxanthine growth inhibition assay was utilized, where the uptake of ^3^H‐hypoxanthine by malaria parasites is used as a surrogate marker for parasite growth. For these assays, stock solutions of the tested compounds were made to concentrations of 20–40 mM in DMSO or water and subsequently diluted in hypoxanthine‐free complete media prior to assay. The assays (in 96‐well plate format) were initiated when most parasites (> 90%) were at the early trophozoite (ring) stage. Parasite cultures (100 μL per well) at 0.5% initial parasitemia and 2% hematocrit in hypoxanthine‐free RPMI1640‐LPLF medium were exposed to ten twofold serial dilutions of the compounds and chloroquine (reference drug) for 96 h, with ^3^H‐hypoxanthine (0.2 μCi well^−1^) added ≈48 h after the beginning of the experiment.

The ^3^H‐hypoxanthine incorporation data were analyzed, and sigmoidal growth inhibition curves were produced by nonlinear regression analysis of the ^3^H‐hypoxanthine incorporation data versus log‐transformed concentrations of the compounds using Graphpad Prism V5.0 software (GraphPad Software Inc. USA). The inhibitory concentration (IC_50_) that results in 50% inhibition of parasite growth was determined. The IC_50_ values were based on three independent experiments, with mean ± SD calculated.

##### Cytotoxicity of the Prodrug in Human Cells

Compound cytotoxicity was evaluated in nontumor human dermal fibroblasts (NHDF), CCRF‐CEM, HL‐60, HeLa, and HepG2 cells. All cell lines were obtained from ATCC (Manassas, VA, USA). The cytotoxicity was assayed using CellTiter‐Glo 2.0 detection reagent (Promega, Madison, USA) according to the manufacturer's protocol. The concentration of prodrug used in this assay was 10 μM. This concentration was chosen as it would more accurately reflect the expected concentration in an in vitro assessment.

## Conflict of Interest

The authors declare no conflict of interest.

## Supporting information

Supplementary Material

## Data Availability

The data that support the findings of this study are available from the corresponding author upon reasonable request.

## References

[1] T. Booden , R. W. Hull , Exp. Parasitol. 1973, 34, 220. 4744840 10.1016/0014-4894(73)90081-7

[2] M. J. Downie , K. Kirk , C. B. Mamoun , Eukaryot Cell. 2008, 7, 1231. 18567789 10.1128/EC.00159-08 PMC2519781

[3] P. Ward , L. Equinet , J. Packer , C. Doerig , BMC Genomics 2004, 5, 79. 15479470 10.1186/1471-2164-5-79 PMC526369

[4] M. B. Cassera , K. Z. Hazleton , P. M. Riegelhaupt , E. F. Merino , M. Luo , M. H. Akbas , V. L. Schramm , J. Biol. Chem. 2008, 283, 32889. 18799466 10.1074/jbc.M804497200 PMC2583302

[5] T. Cheviet , I. Lefebvre‐Tournier , S. Wein , S. Peyrottes , J. Med. Chem. 2019, 62, 8365. 30964283 10.1021/acs.jmedchem.9b00182

[6] S. A. Queen , D. V. Jagt , P. Reyes , Mol. Biochem. Parasitol. 1988, 30, 123. 3050515 10.1016/0166-6851(88)90105-3

[7] D. T. Keough , A. L. Ng , D. J. Winzor , B. T. Emmerson , J. de Jersey , Mol. Biochem. Parasitol. 1999, 98, 29. 10029307 10.1016/s0166-6851(98)00139-x

[8] D. T. Keough , T. Skinner‐Adams , M. K. Jones , A. L. Ng , I. M. Brereton , L. W. Guddat , J. de Jersey , J. Med. Chem. 2006, 49, 7479. 17149876 10.1021/jm061012j

[9] K. Z. Hazleton , M. C. Ho , M. B. Cassera , K. Clinch , D. R. Crump , E. F. M. I. Rosario Jr., S. C. Almo , P. C. Tyler , V. L. Schramm , Chem. Biol. 2012, 19, 721. 22726686 10.1016/j.chembiol.2012.04.012 PMC3397391

[10] D. T. Keough , D. Hocková , A. Holý , L. M. Naesens , T. S. Skinner‐Adams , J. Jersey , L. W. Guddat , J. Med. Chem. 2009, 52, 4391. 19527031 10.1021/jm900267n

[11] D. T. Keough , D. Rejman , R. Pohl , E. Zborníková , D. Hocková , T. Croll , M. D. Edstein , G. W. Birrell , M. Chavchich , L. M. J. Naesens , G. K. Pierens , I. M. Brereton , L. W. Guddat , ACS Chem. Biol. 2018, 13, 82. 29161011 10.1021/acschembio.7b00916

[12] D. T. Keough , M. Petrová , G. King , M. Kratochvíl , R. Pohl , E. Doleželová , A. Zíková , L. W. Guddat , D. Rejman , J. Med. Chem. 2024, 67, 7158. 38651522 10.1021/acs.jmedchem.4c00021 PMC11089518

[13] W. Shi , C. M. Li , P. C. Tyler , R. H. Furneaux , C. Grubmeyer , V. L. Schramm , S. C. Almo , Nat. Struct. Biol. 1999, 6, 588. 10360366 10.1038/9376

[14] W. Shi , C. M. Li , P. C. Tyler , R. H. Furneaux , S. M. Cahill , M. E. Girvin , C. Grubmeyer , V. L. Schramm , S. C. Almo , Biochemistry 1999, 38, 9872. 10433693 10.1021/bi990664p

[15] J. K. Meena , J. H. Wang , N. J. Neill , D. Keough , N. Putluri , P. Katsonis , A. M. Koire , H. Lee , E. A. Bowling , S. Tyagi , M. Orellana , R. Dominguez‐Vidana , H. Li , K. Eagle , C. Danan , H. C. Chung , A. D. Yang , W. Wu , S. J. Kurley , M. B., J. R. Z. Ho , C. M. Olson , K. L. Meerbrey , O. Lichtarge , A. Sreekumar , C. C. Dacso , L. W. Guddat , D. Rejman , D. Hocková , Z. Janeba , L. M. Simon , C. Y. Lin , M. C. Pillon , T. F. Westbrook , Cancer Discov. 2024, 14, 1699. 39193992 10.1158/2159-8290.CD-22-0649 PMC11372365

[16] C. M. Li , P. C. Tyler , R. H. Furneaux , G. Kicska , Y. Xu , C. Grubmeyer , M. E. Girvin , V. L. Schramm , Nat. Struct. Biol. 1999, 6, 582. 10360365 10.1038/9367

[17] D. T. Keough , P. Špaček , D. Hocková , T. Tichý , S. Vrbková , L. Slavětínská , Z. Janeba , L. Naesens , M. D. Edstein , M. Chavchich , T. H. Wang , J. de Jersey , L. W. Guddat , J. Med. Chem. 2013, 56, 2513. 23448281 10.1021/jm301893b

[18] P. Špaček , D. T. Keough , M. Chavchich , M. Dračínský , Z. Janeba , L. Naesens , M. D. Edstein , L. W. Guddat , D. Hocková , J. Med. Chem. 2017, 60, 7539. 28813147 10.1021/acs.jmedchem.7b00926

[19] D. Hocková , D. T. Keough , Z. Janeba , T. H. Wang , J. de Jersey , L. W. Guddat , J. Med. Chem. 2012, 55, 6209. 22725979 10.1021/jm300662d

[20] D. T. Keough , D. Hocková , Z. Janeba , T. H. Wang , L. Naesens , M. D. Edstein , M. Chavchich , L. W. Guddat , J. Med. Chem. 2015, 58, 827. 25494538 10.1021/jm501416t

[21] W. S. Eng , D. Hocková , P. Špaček , Z. Janeba , N. P. West , K. Woods , L. M. Naesens , D. T. Keough , L. W.Guddat , J. Med. Chem. 2015, 58, 4822. 25915781 10.1021/acs.jmedchem.5b00611

[22] J. Frydrych , J. Skácel , M. Šmídková , H. Mertlíková‐Kaiserová , M. Dračínský , R. Gnanasekaran , M. Lepšík , M. Soto‐Velasquez , V. J. Watts , Z. Janeba , ChemMedChem 2018, 13, 199. 29235265 10.1002/cmdc.201700715

[23] J. Frydrych , L. P.štová Slavětínská , M. Dračínský , Z. Janeba , Molecules 2020, 25, 4307. 32961820 10.3390/molecules25184307 PMC7571146

[24] F. Kalčic , M. Dračínský , Z. Janeba , Z. Org. Biomol. Chem. 2021, 19, 6958. 10.1039/d1ob00751c 34032256

[25] F. Kalčic , J. Frydrych , E. Doleželová , M. Slapničková , P. Pachl , L. P.štová Slavětínská , M. Dračínský , D. Hocková , A. Zíková , Z. Janeba , Eur. J. Med. Chem. 2021, 225, 113798. 34482272 10.1016/j.ejmech.2021.113798

[26] D. T. Keough , I. M. Brereton , J. de Jersey , L. W. Guddat , J. Mol. Biol. 2025, 351, 170. 10.1016/j.jmb.2005.05.061 15990111

[27] G. K. Balendiran , J. A. Molina , Y. Xu , J. Torres‐Martinez , R. Stevens , P. J. Focia , A. E. Eakin , J. C. Sacchettini , S. P. Craig , Protein Sci. 1999, 8, 1023. 10338013 10.1110/ps.8.5.1023 PMC2144341

[28] J. C. Eads , G. Scapin , Y. Xu , C. Grubmeyer , J. C. Sacchettini , Cell 1994, 78, 325. 8044844 10.1016/0092-8674(94)90301-8

[29] T. Klejch , D. T. Keough , G. King , E. Doleželová , M. Česnek , M. Buděšínský , A. Zíková , Z. Janeba , L. W. Guddat , D. Hockova , J. Med. Chem. 2022, 65, 4030. 35175749 10.1021/acs.jmedchem.1c01881

[30] N. M. O’Boyle , M. Banck , C. A. James , C. Morley , T. Vandermeersch , G. R. Hutchison , J Cheminform. 2011, 3, 33. 21982300 10.1186/1758-2946-3-33 PMC3198950

[31] G. Jones , P. Willett , R. C. Glen , A. R. Leach , R. Taylor , J. Mol Biol. 1997, 267, 727. 9126849 10.1006/jmbi.1996.0897

[32] M. M. Kaiser , D. Hocková , T. H. Wang , M. Dračínský , L. Poštová‐Slavětínská , E. Procházková , M. D. Edstein , M. Chavchich , D. T. Keough , L. W. Guddat , Z. Janeba , ChemMedChem 2015, 10, 1707. 26368337 10.1002/cmdc.201500322

[33] W. Trager , J. B. Jensen , Science 1976, 193, 673. 781840 10.1126/science.781840

[34] C. Lambros , J. P. Vanderberg , J. Parasitol. 1979, 65, 418. 383936

