# Supplementary material for: C1′‐Branched Acyclic Nucleoside Phosphonates as Inhibitors of Plasmodium Falciparum 6‐Oxopurine Phosphoribosyltransferase
Source: ChemMedChem. 2025 Aug 22;20(19):e202500575. doi: 10.1002/cmdc.202500575 (PMC12503909; doi:10.1002/cmdc.202500575)
Supplement: Supplementary file 1 — Supplementary Material [file CMDC-20-e202500575-s001.pdf]

C1'-Branched Acyclic Nucleoside Phosphonates as Inhibitors of *Plasmodium falciparum* 6-Oxopurine Phosphoribosyltransferase

Jan Frydrych,[a] Dianne T. Keough,[b] Haojun Xia,[b] Lenka Poštová Slavětínská,[a] Martin Dračínský,[a] Michal Česnek,[a] Jye Travis,[c] Marina Chavchich,[c] Michael Edstein,[c] Dana Hocková,[a] Luke W. Guddat,*[b] and Zlatko Janeba*[a]

[a] *Institute of Organic Chemistry and Biochemistry of the Czech Academy of Sciences, Flemingovo nám. 2, 160 00 Prague 6, Czech Republic*

[b] *School of Chemistry and Molecular Biosciences, The University of Queensland, Brisbane 4072, Australia*

[c] *Department of Drug Evaluation, Australian Defence Force Malaria and Infectious Disease Institute, Enoggera, Brisbane, Queensland 4051, Australia*

* Corresponding author. luke.guddat@uq.edu.au (L. W. Guddat), zlatko.janeba@uochb.cas.cz (Z. Janeba)

TABLE OF CONTENTS

Viability of prepared prodrugs in selected human cell lines (**Table S1**) ….……………………… S2

^{1}H NMR, ^{13}C NMR, ^{31}P NMR, and HRMS spectra of prepared compounds S3

Table S1. Viability of compounds **19a** and **21a**–**21g** in five human cell lines (HL60, CCRF-CEM, HeLa, HepG2, and NHDF) at 10 μM concentration of tested compounds versus untreated control.[a)]

Prodrug	HL-60<br>% control	CCRF-CEM<br>% control	HeLa<br>% control	HepG2<br>% control	NHDF<br>% control
**19a**	112 ± 1	106 ± 1	105 ± 1	116 ± 1	114 ± 5
**21a**	103 ± 0	101 ± 2	97 ± 2	98 ± 3	104 ± 8
**21b**	100 ± 2	97 ± 1	91 ± 2	93 ± 4	94 ± 8
**21c**	104 ± 2	100 ± 2	97 ± 2	97 ± 2	102 ± 6
**21d**	105 ± 1	99 ± 4	100 ± 2	107 ± 2	112 ± 1
**21e**	110 ± 1	105 ± 2	105 ± 1	106 ± 3	110 ± 7
**21f**	105 ± 2	103 ± 1	106 ± 1	110 ± 2	116 ± 4
**21g**	107 ± 2	105 ± 2	106 ± 2	111 ± 6	114 ± 3

[a)]Data represent the mean ± SD of at least three independent experiments.

Sodium salt of (2-(2-(hypoxanthin-9-yl)-2-(2-(phosphonatomethoxy)ethoxy)ethoxy)ethyl)phosphonic acid (18a)

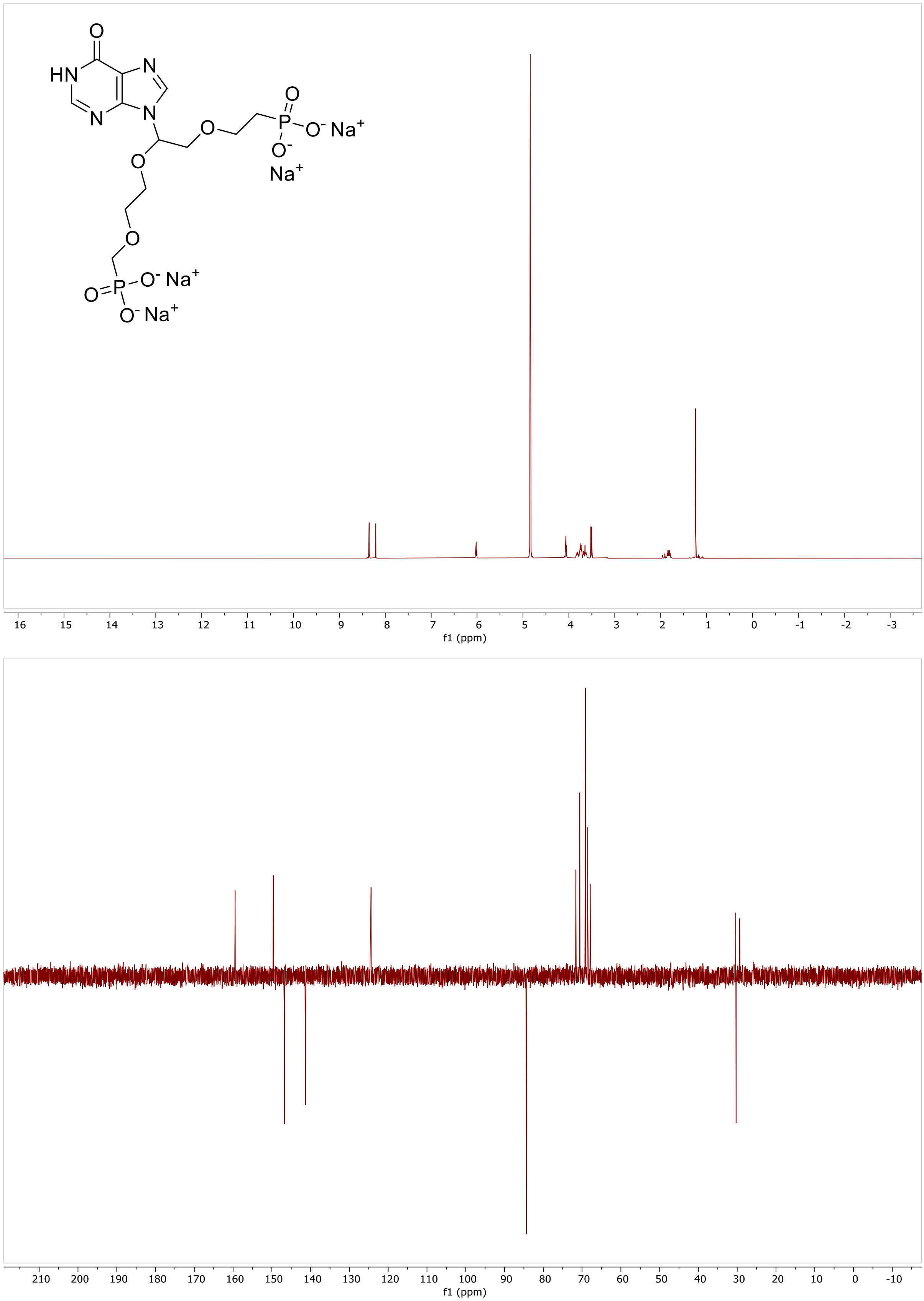

Fig. S1. ^{1}H (top) and ^{13}C (bottom) NMR spectra of compound **18a** (at rt) in D_2O containing 0.1% of *terc*-butyl alcohol as an internal standard.

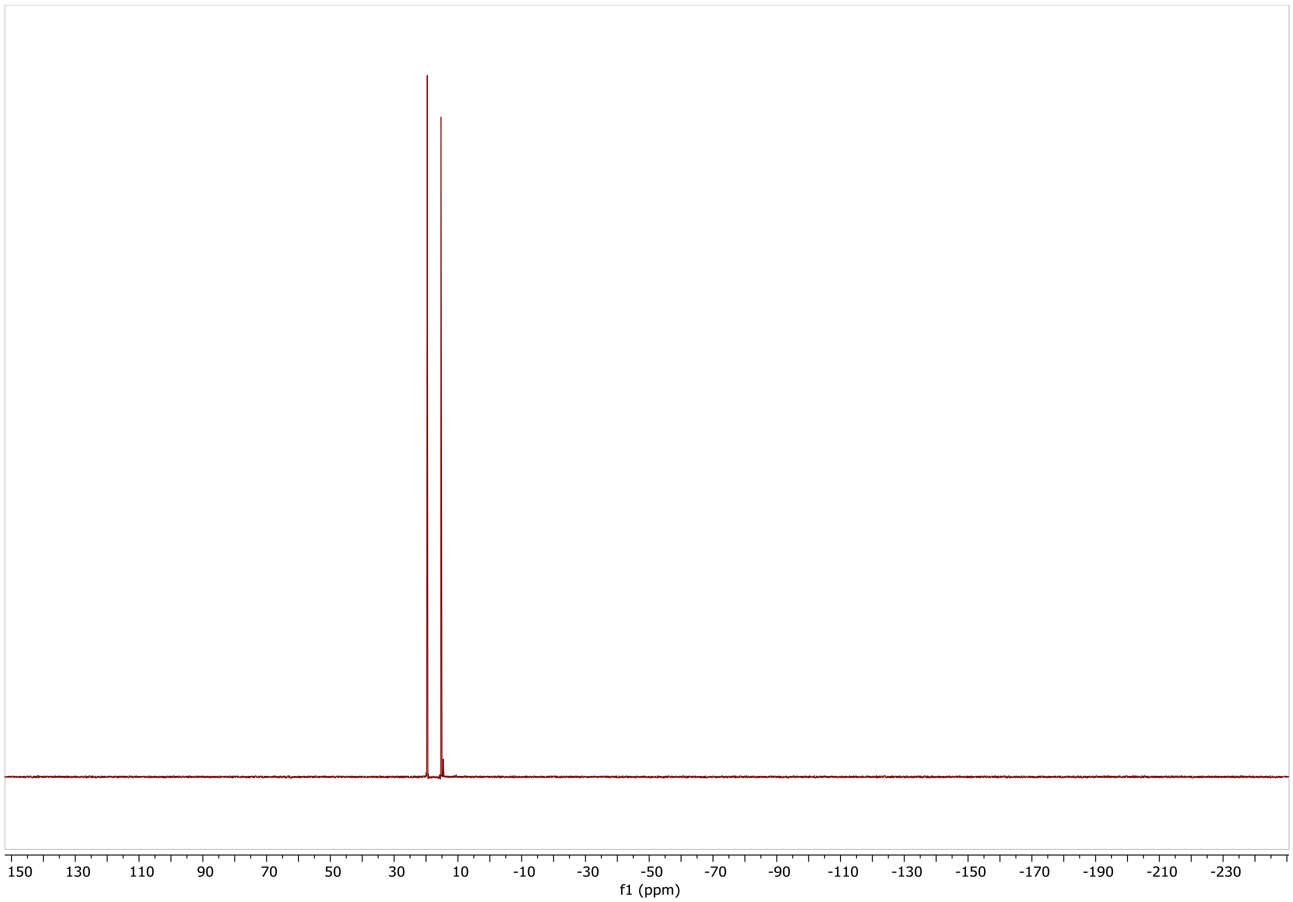

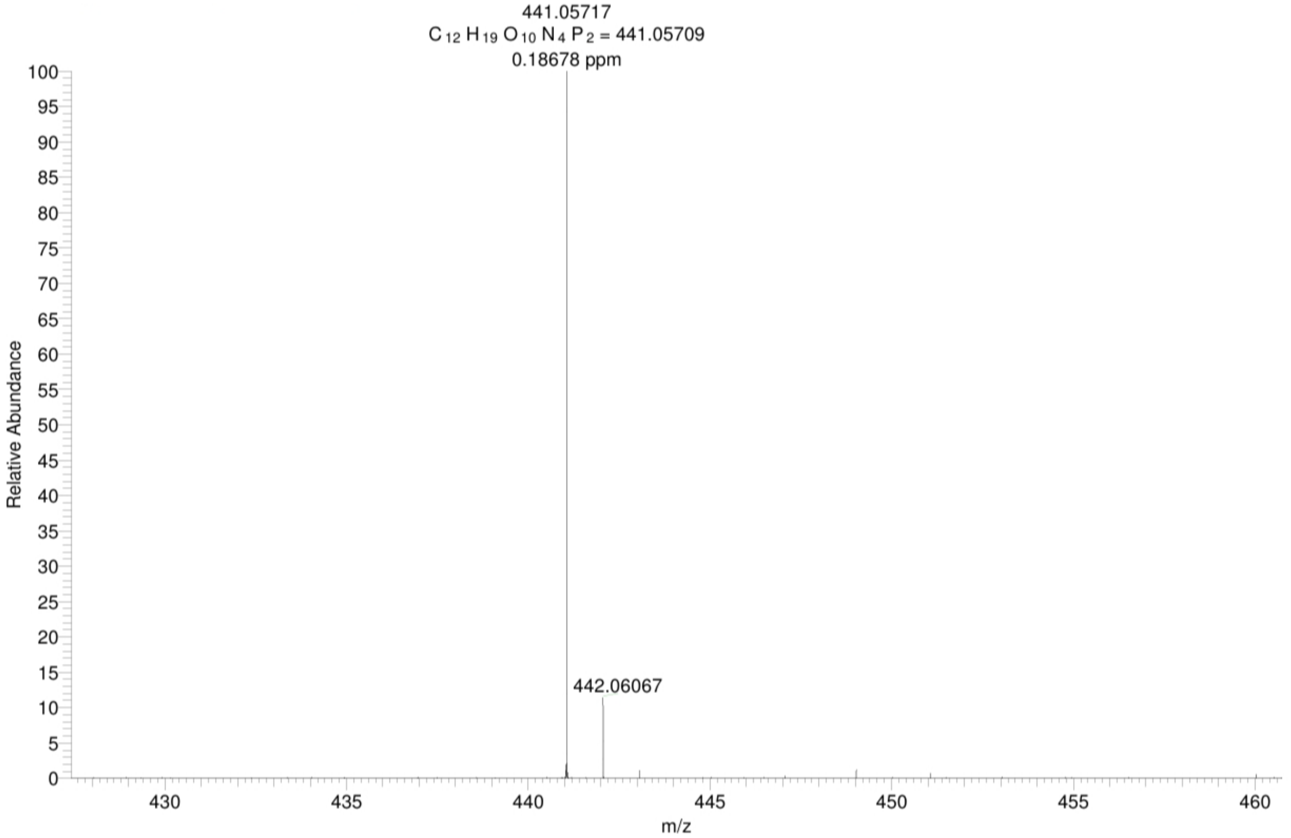

Fig. S2. ^{31}P NMR of compound **18a** (at rt) in D_2O containing 0.1% of *terc*-butyl alcohol as an internal standard (top) and high resolution mass spectrum (HRMS, bottom) of compound **18a**.

Sodium salt of (2-(2-(guanin-9-yl)-2-(2-(phosphonatomethoxy)ethoxy)ethoxy)ethyl)phosphonic acid (18b)

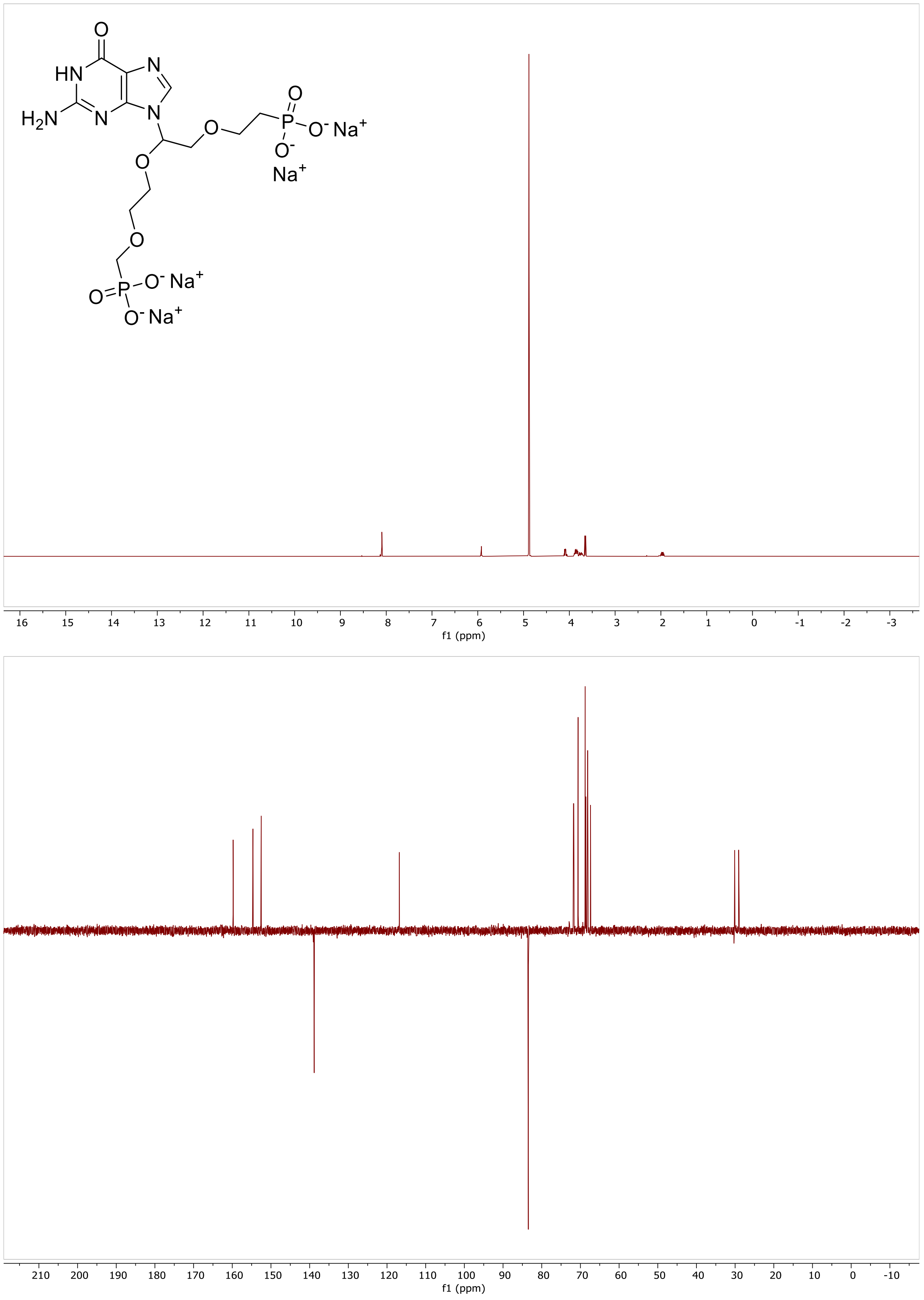

Fig. S3. ^{1}H (top) and ^{13}C (bottom) NMR spectra of compound **18b** (at rt) in D_2O containing 0.1% of dioxane as an internal standard.

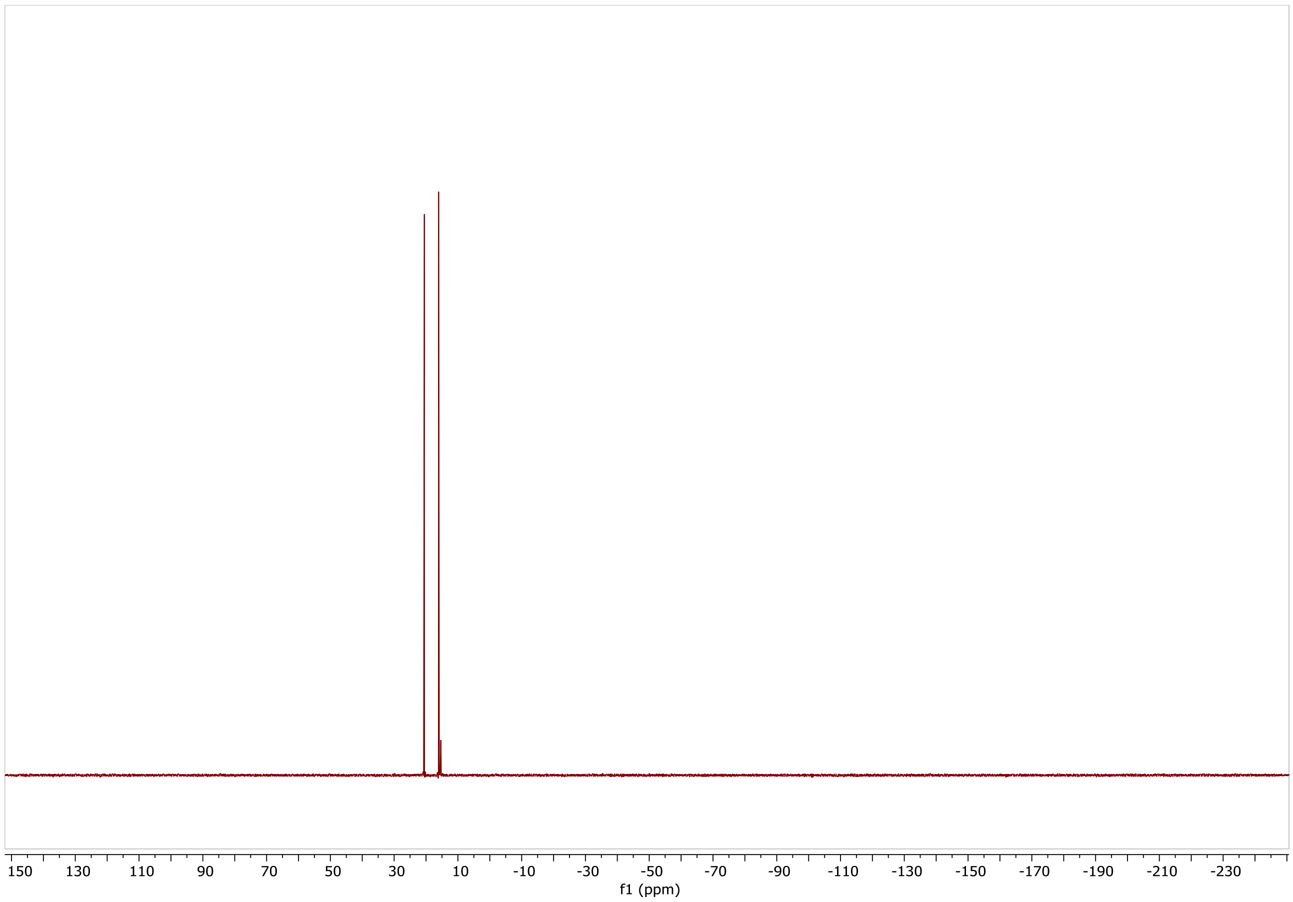

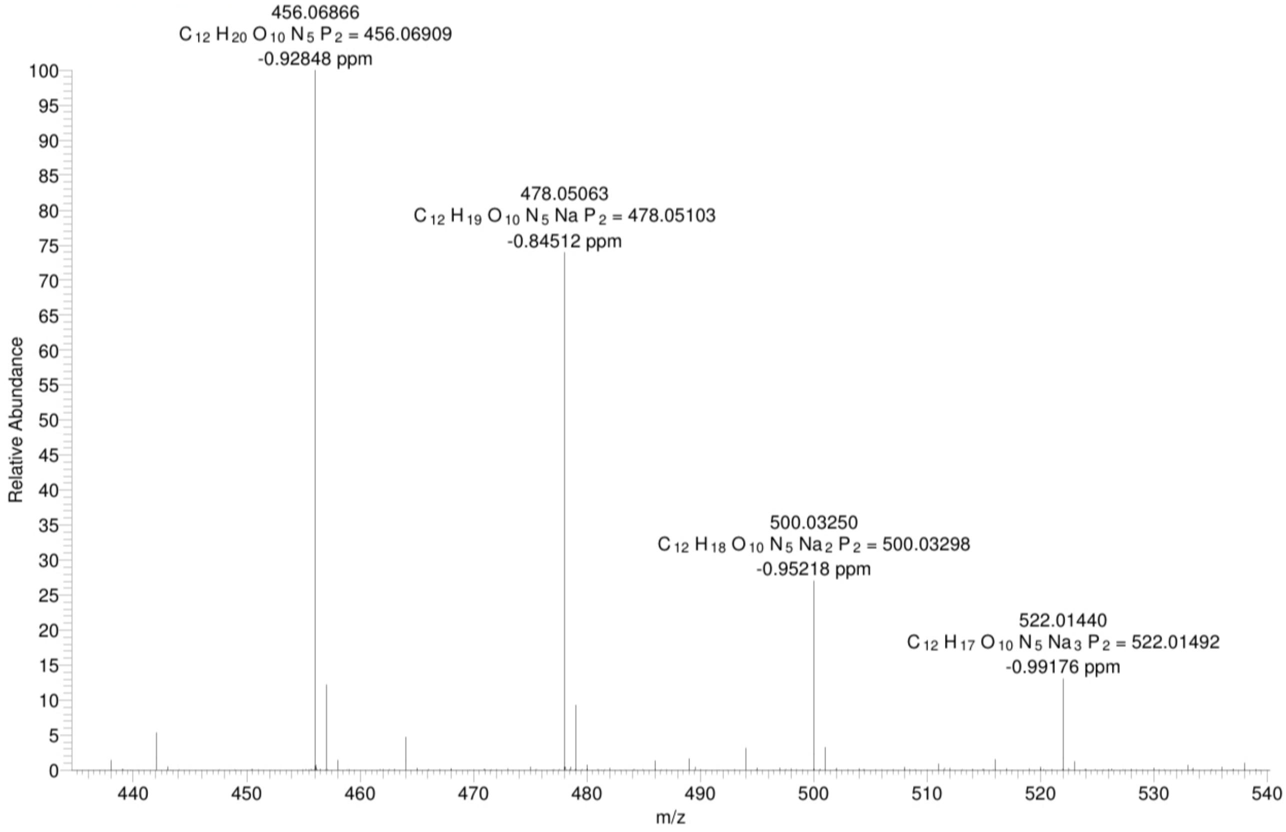

Fig. S4. ^{31}P NMR of compound **18b** (at rt) in D_2O containing 0.1% dioxane as an internal standard (top) and high resolution mass spectrum (HRMS, bottom) of compound **18b**.

Tetra-(L-phenylalaninate ethyl ester) prodrug of (2-(2-(hypoxanthin-9-yl)-2-(2-(phosphonatomethoxy)ethoxy)ethoxy)ethyl)phosphonic acid (19a)

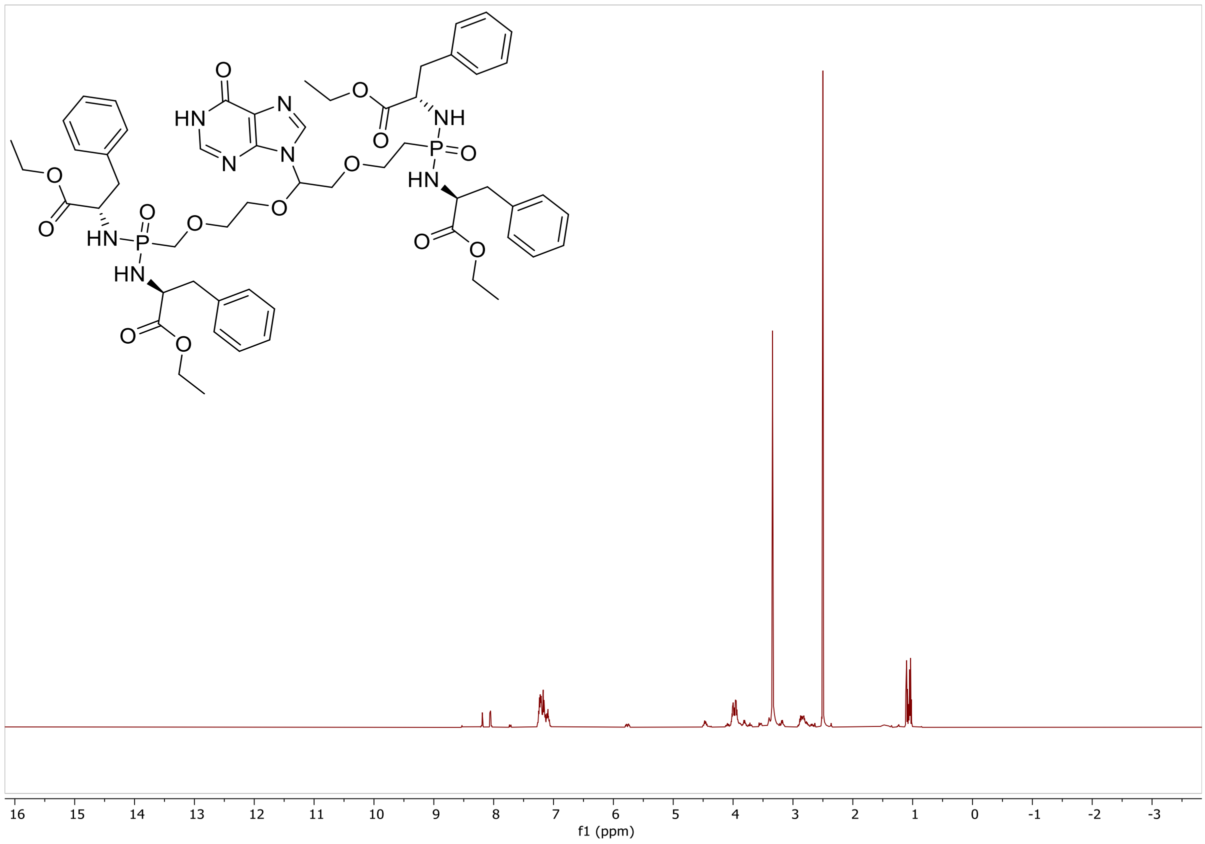

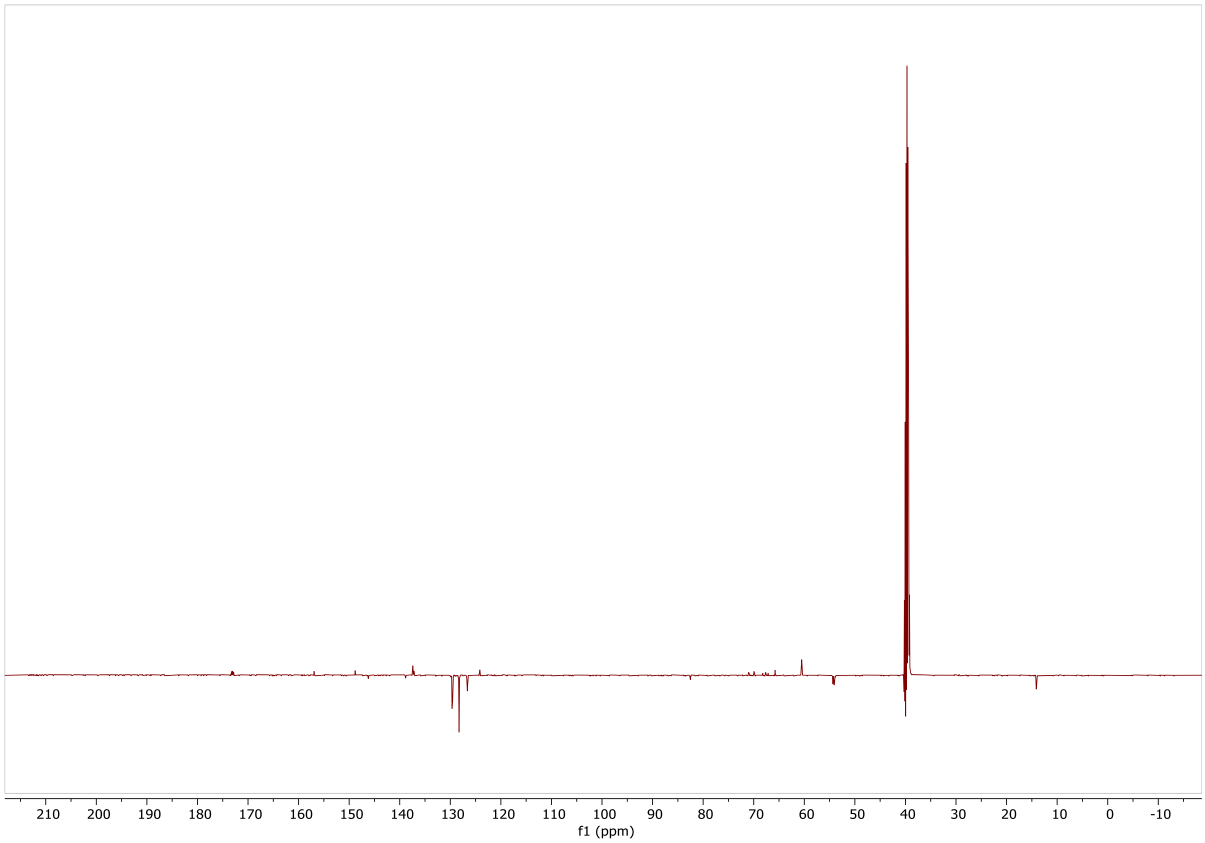

Fig. S5. ^{1}H (top) and ^{13}C (bottom) NMR spectra of compound **19a** (at rt) in DMSO.

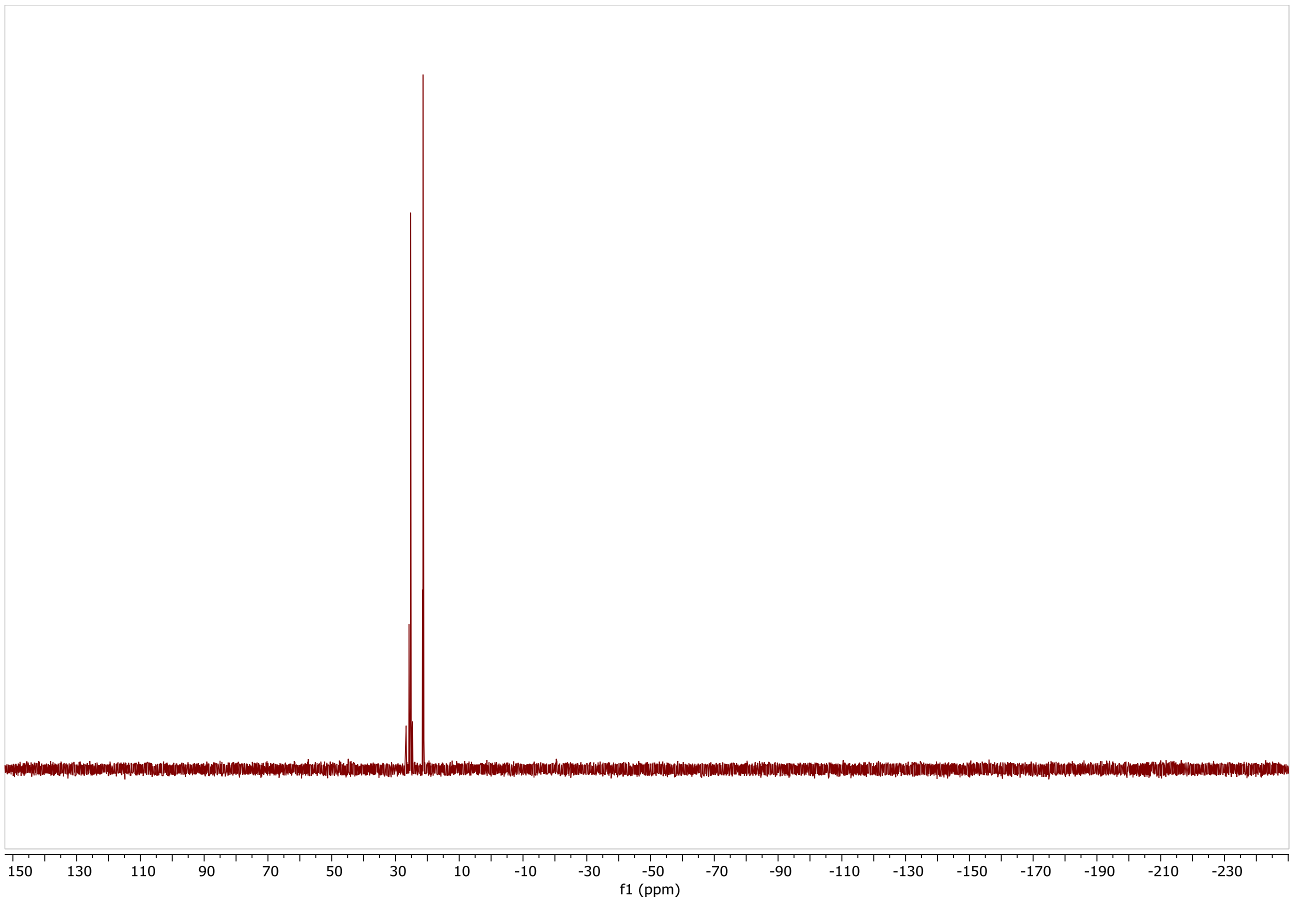

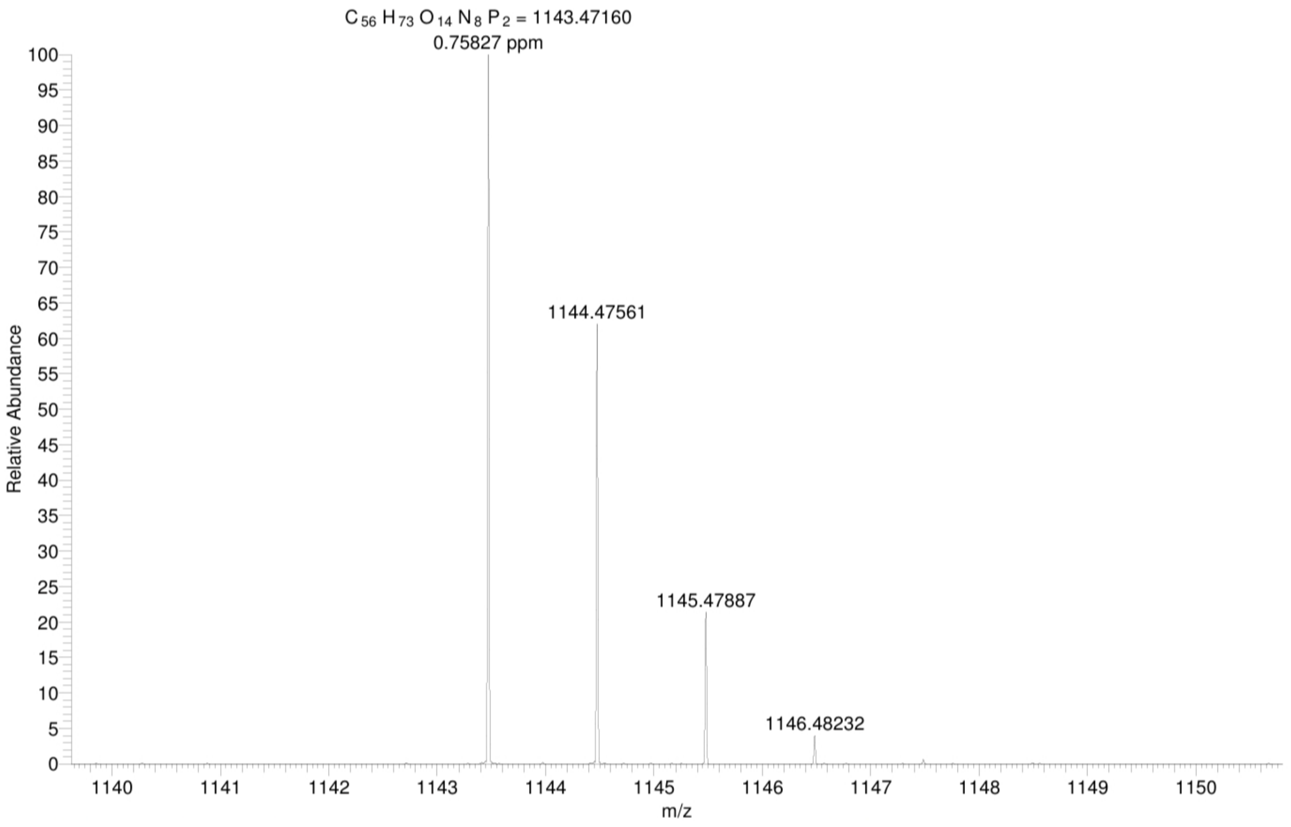

Fig. S6. ^{31}P NMR of compound **19a** (at rt) in DMSO (top) and high resolution mass spectrum (HRMS, bottom) of compound **19a**.

Tetra-(L-phenylalaninate ethyl ester) prodrug of (2-(2-(guanin-9-yl)-2-(2-(phosphonatomethoxy)ethoxy)ethoxy)ethyl)phosphonic acid (19b)

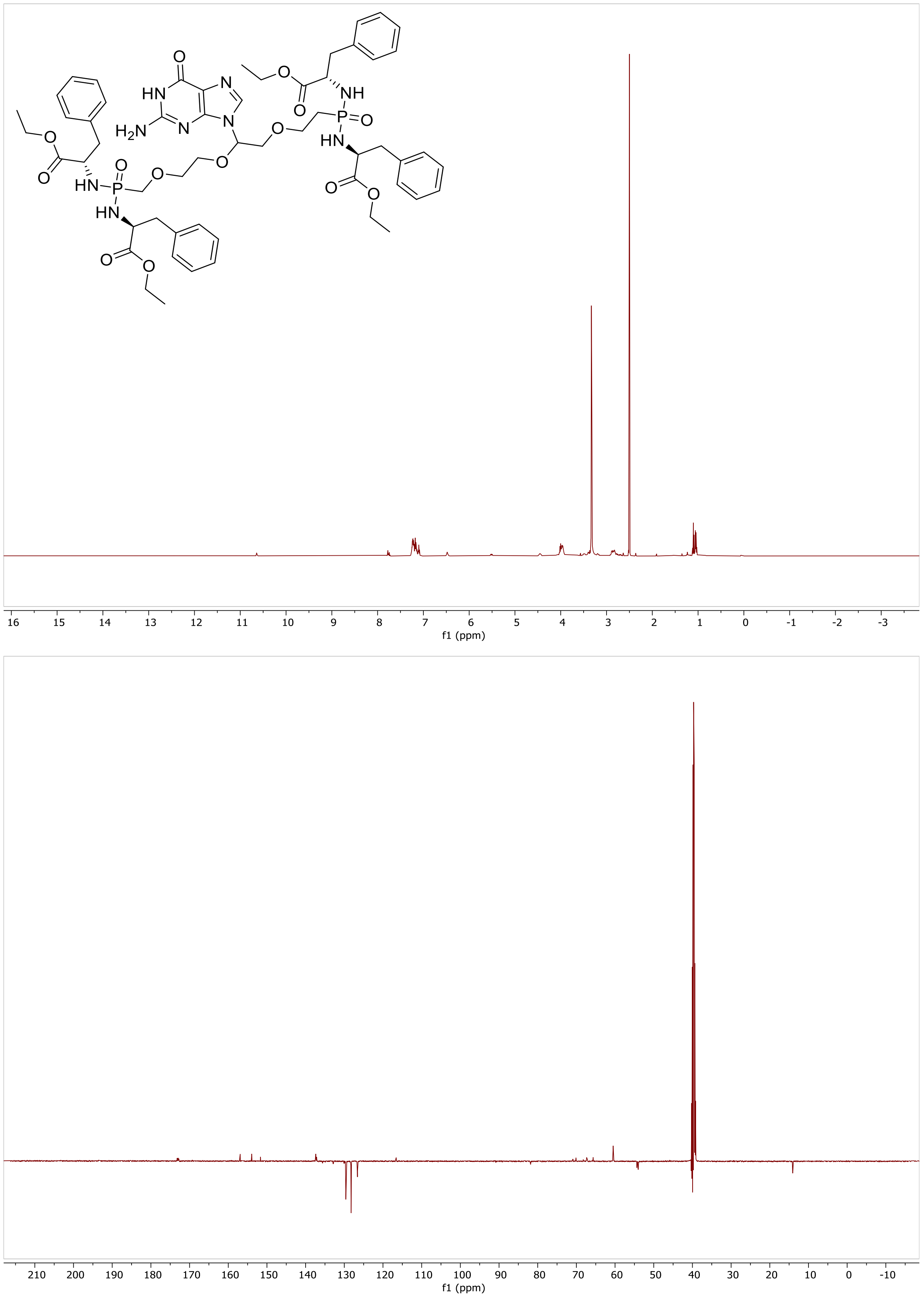

Fig. S7. ^{1}H (top) and ^{13}C (bottom) NMR spectra of compound **19b** measured at room temperature in DMSO.

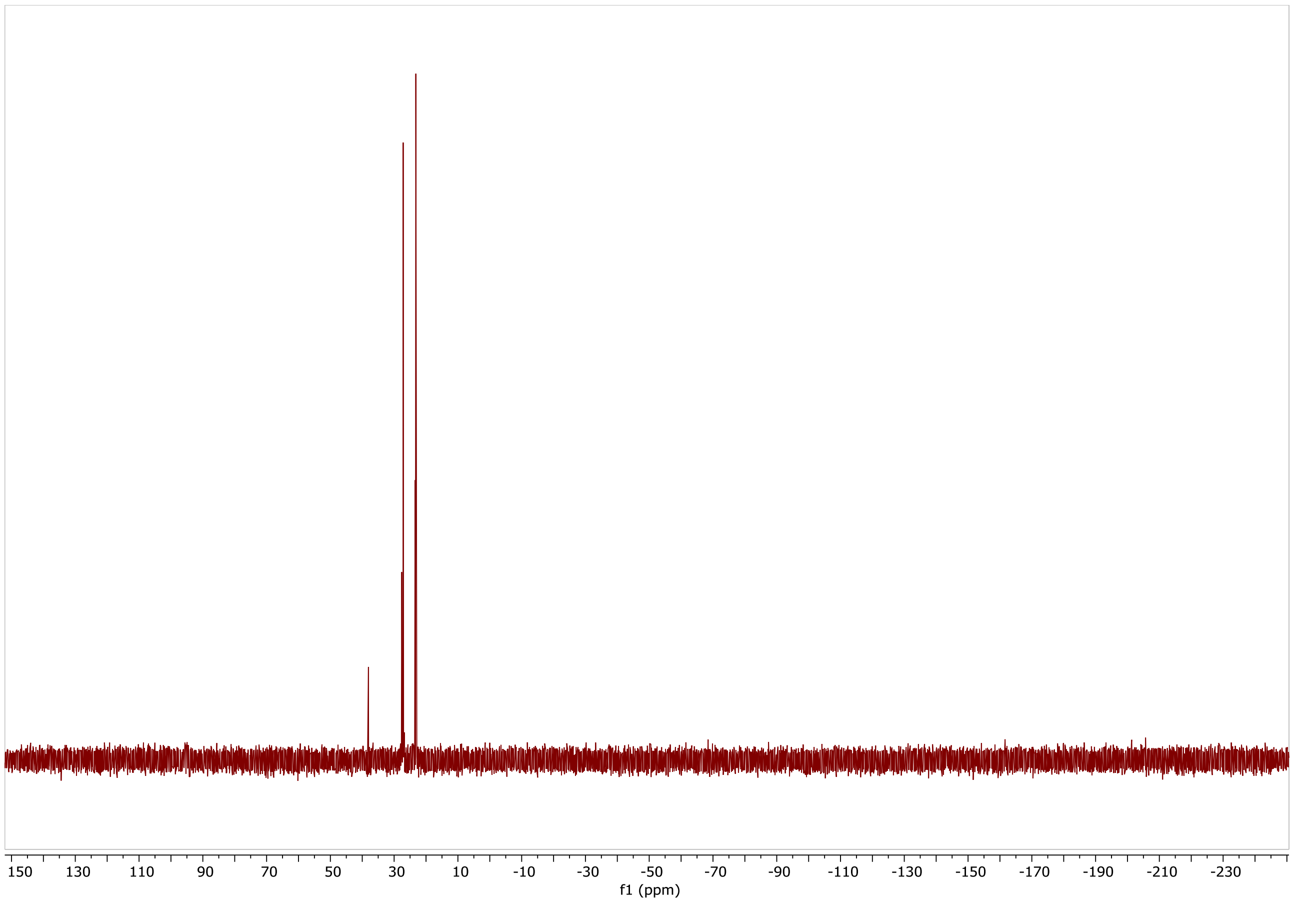

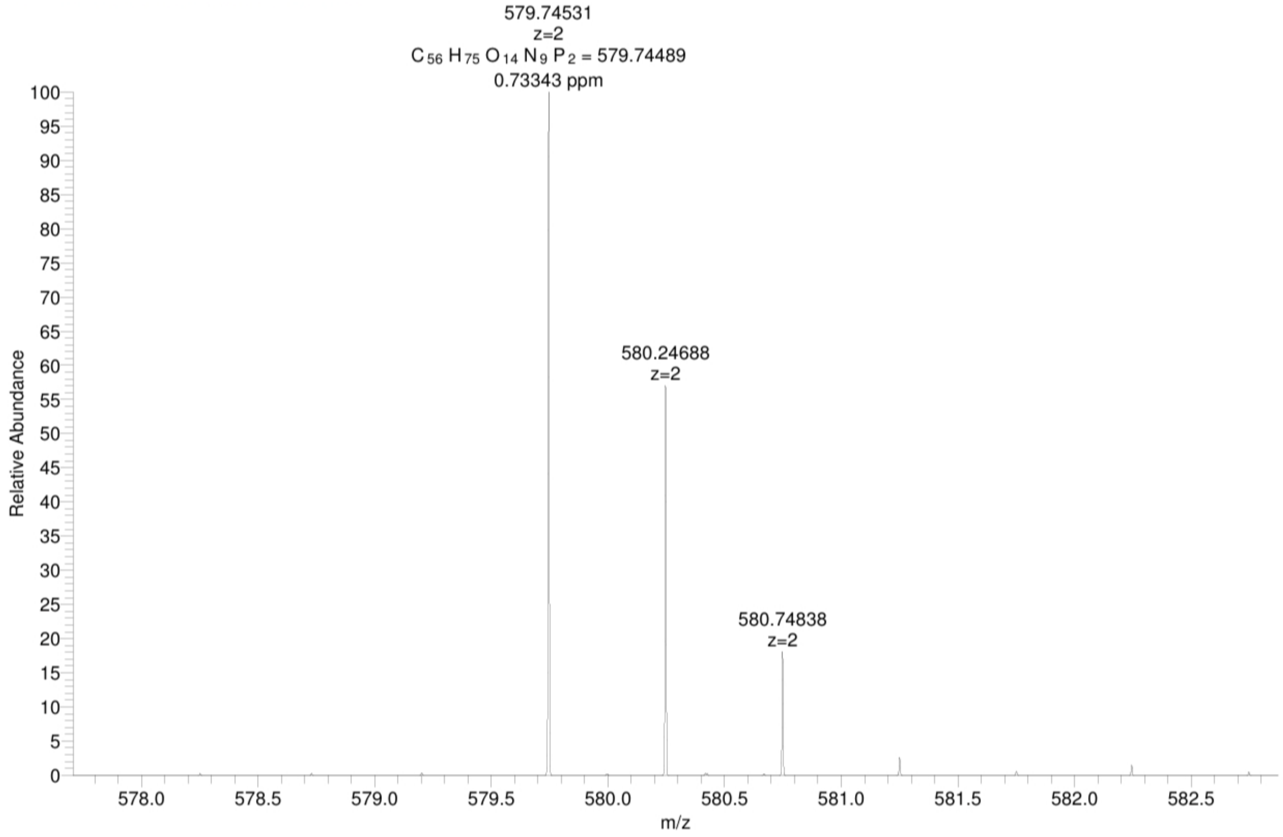

Fig. S8. ^{31}P NMR of compound **19b** (at rt) in DMSO (top) and high resolution mass spectrum (HRMS, bottom) of compound **19b**.

Sodium salt of ((2-(2-hydroxyethoxy)-2-(hypoxantine-9-yl)ethoxy)methyl) phosphonic acid (20a)

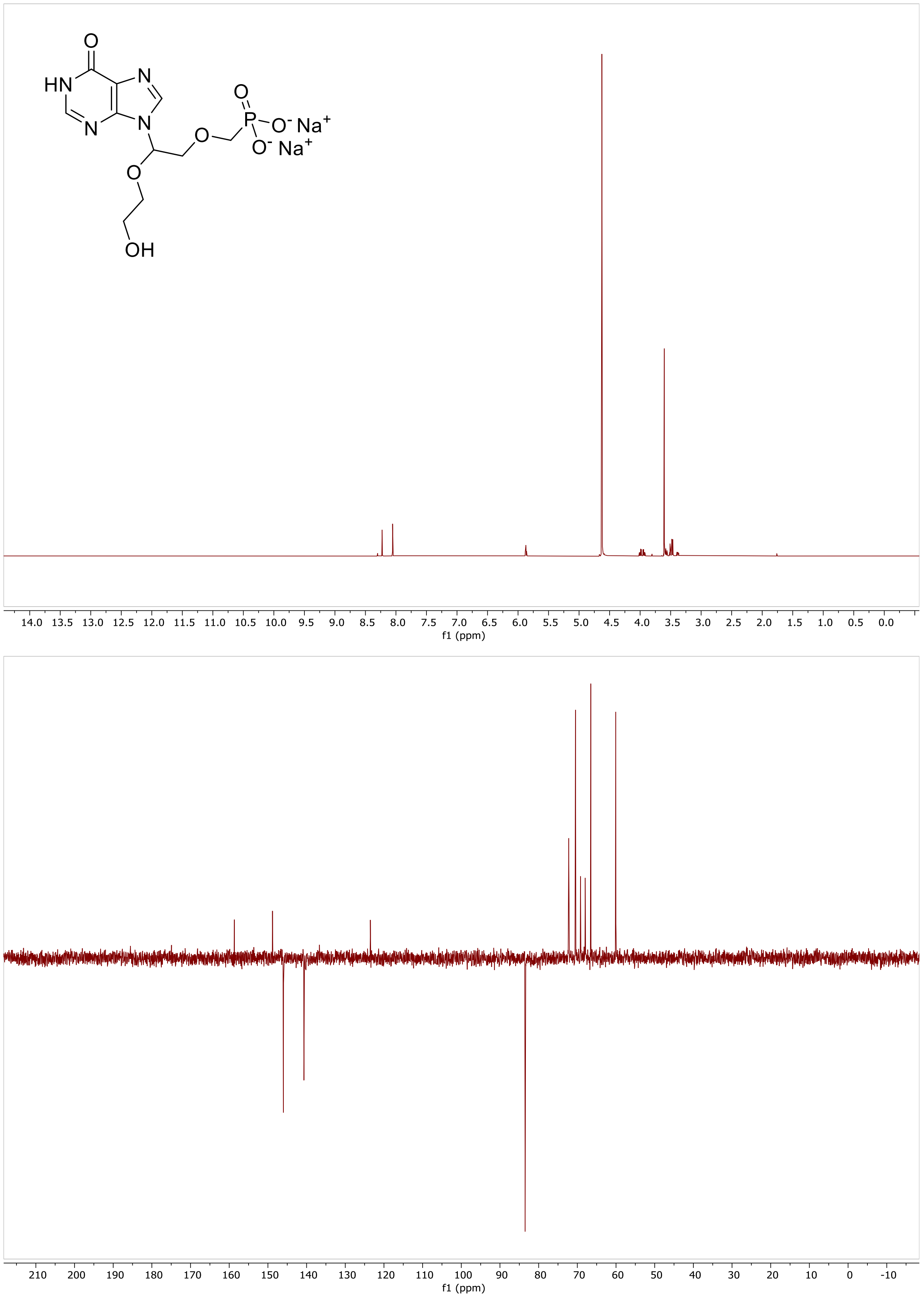

Fig. S9. ^{1}H (top) and ^{13}C (bottom) NMR spectra of compound **20a** (at rt) in D_2O containing 0.1% of dioxane as an internal standard.

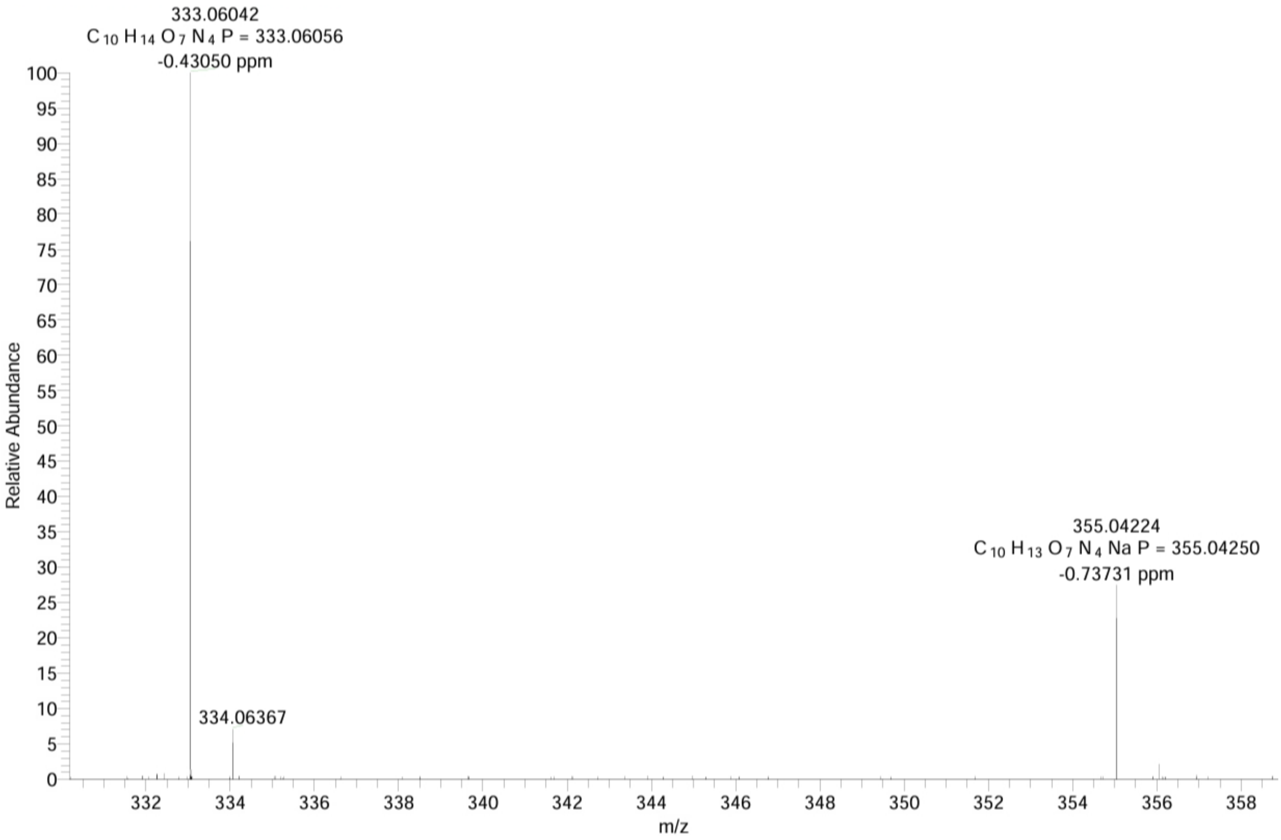

Fig. S10. High resolution mass spectrum (HRMS) of compound **20a**.

Sodium salt of ((2-(guanin-9-yl)-2-(2-hydroxyethoxy)ethoxy)methyl)phosphonic acid (20b)

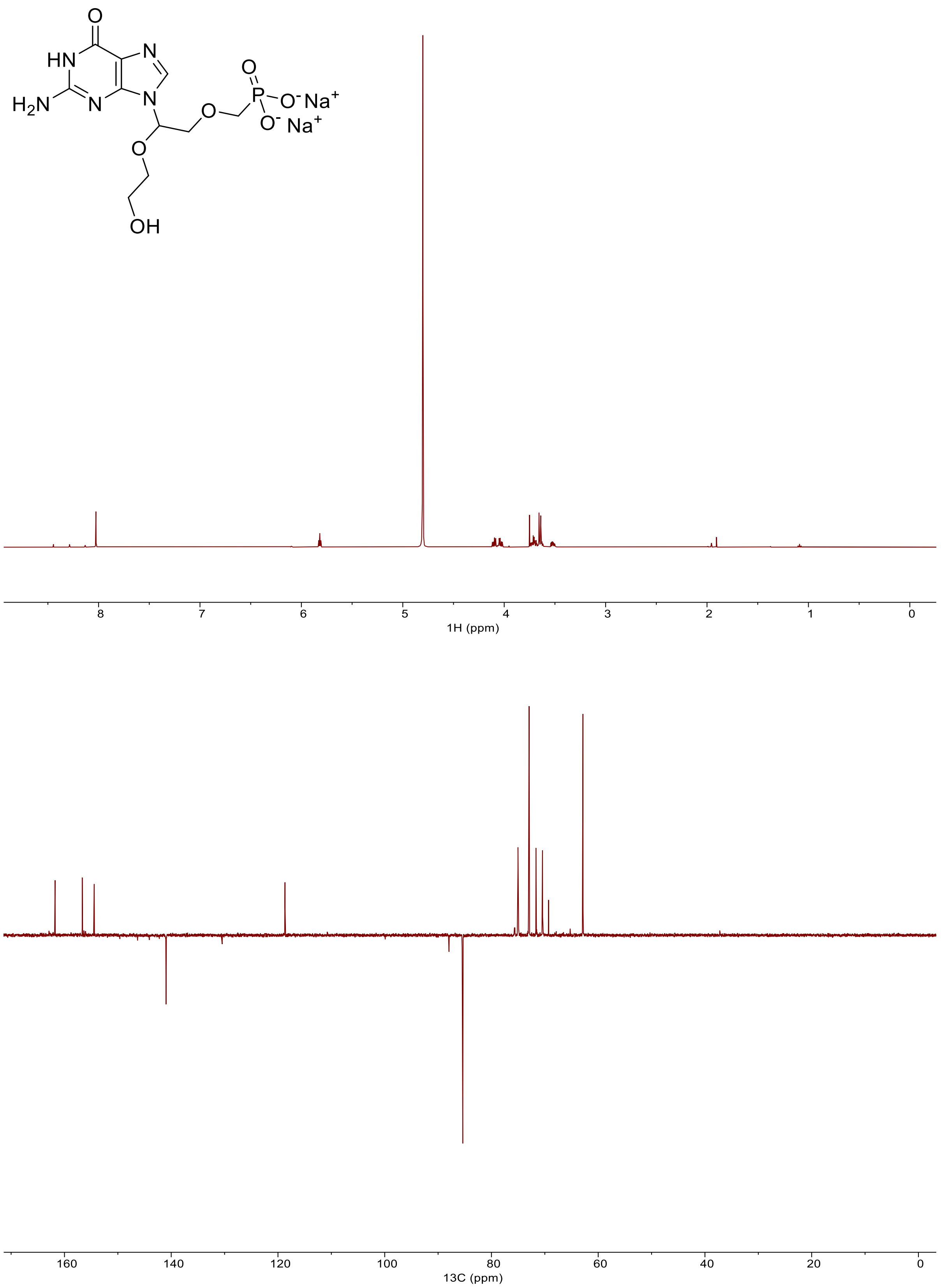

Fig. S11. ^{1}H (top) and ^{13}C (bottom) NMR spectra of compound **20b** (at rt) in D_2O containing 0.1% of dioxane as an internal standard.

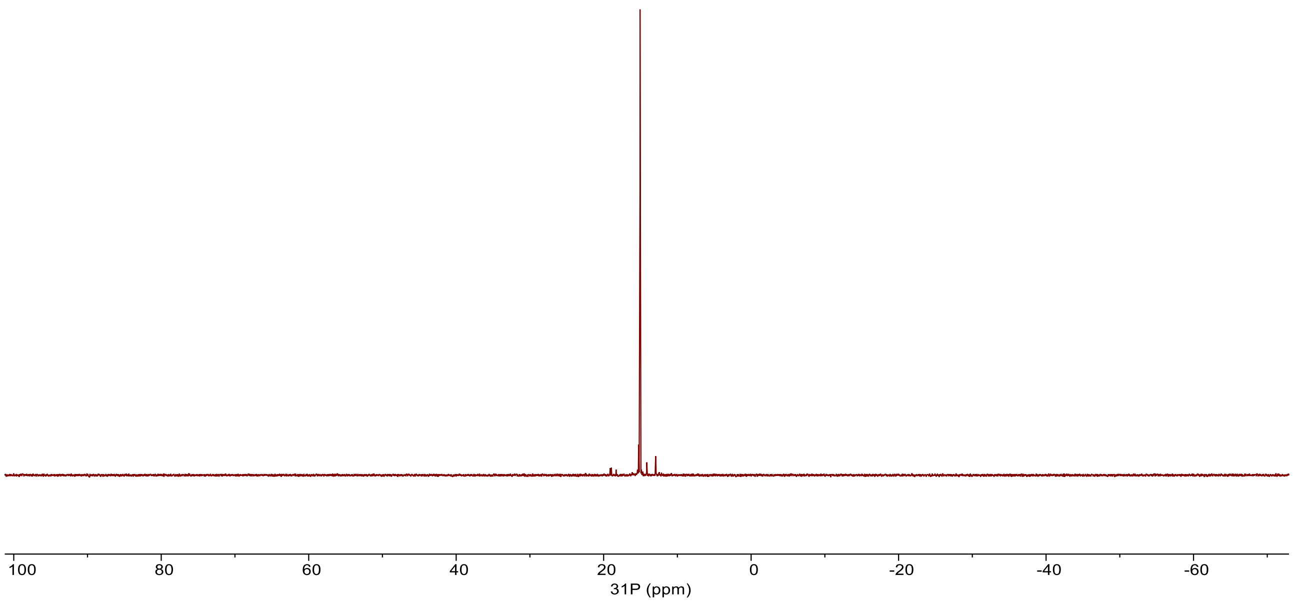

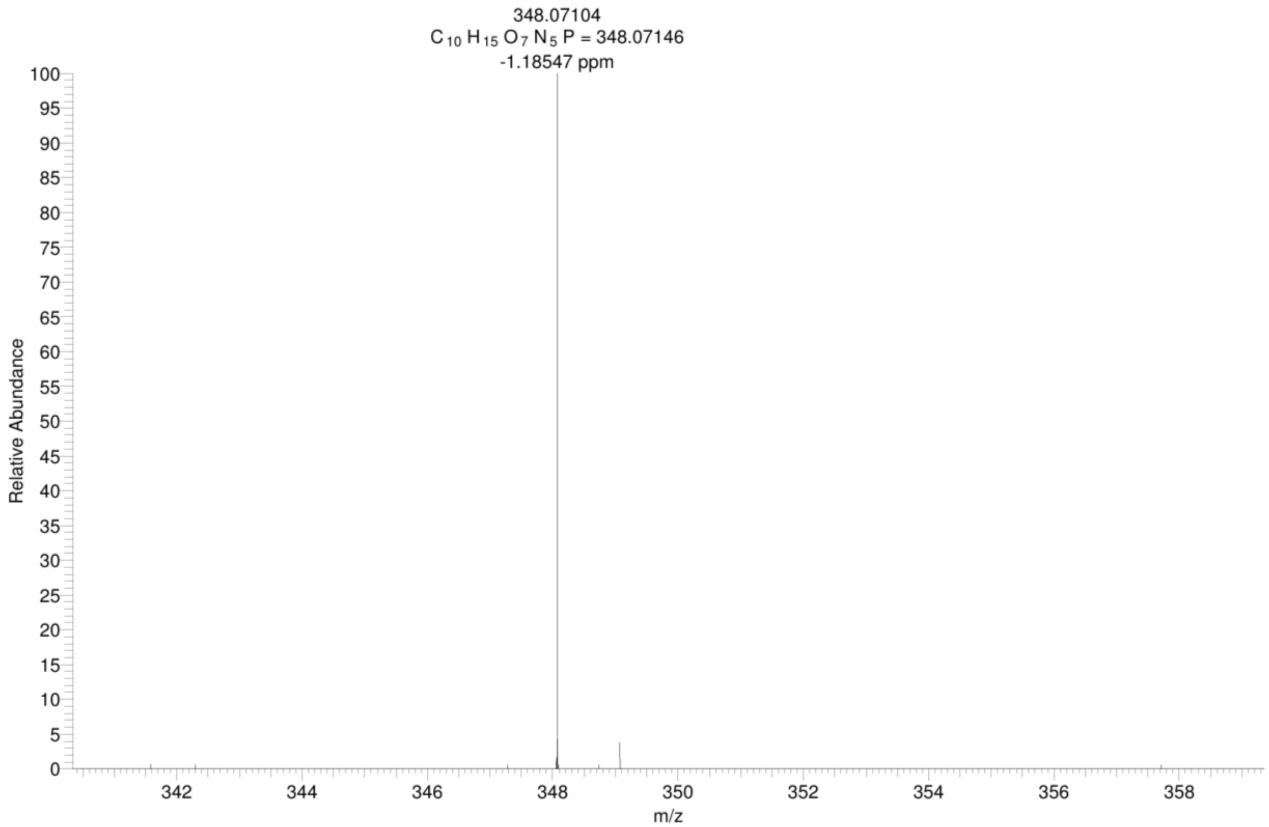

Fig. S12. ^{31}P NMR of compound **20b** (at rt) in D_2O containing 0.1% dioxane as an internal standard (top) and high resolution mass spectrum (HRMS, bottom) of compound **20b**.

Sodium salt of (2-(2-(2-hydroxyethoxy)-2-(hypoxanthin-9-yl)ethoxy)ethyl)phosphonic acid (20c)

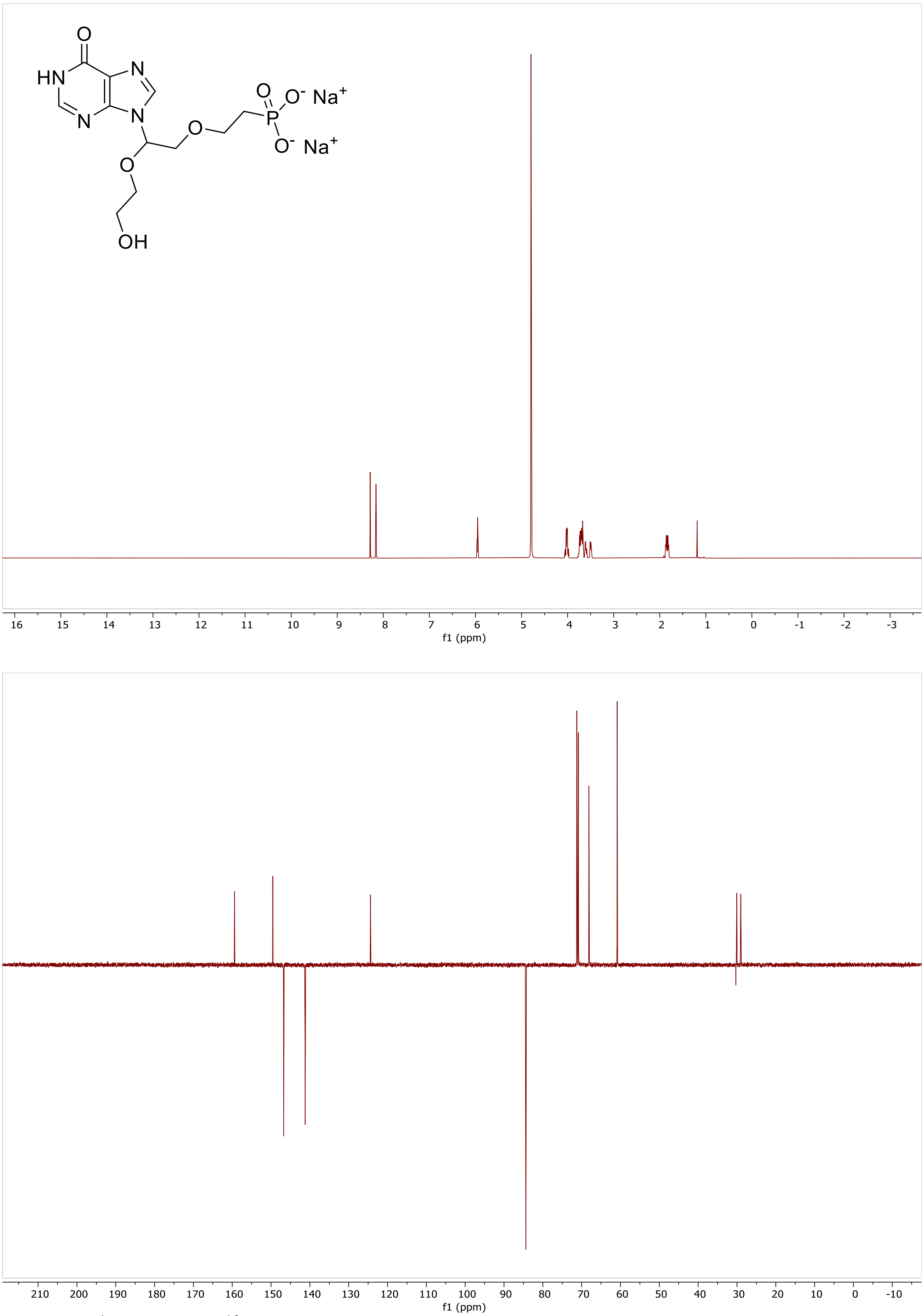

Fig. S13. ^{1}H (top) and ^{13}C (bottom) NMR spectra of compound **20c** (at rt) in D_2O containing 0.1% of *terc*-butyl alcohol as an internal standard.

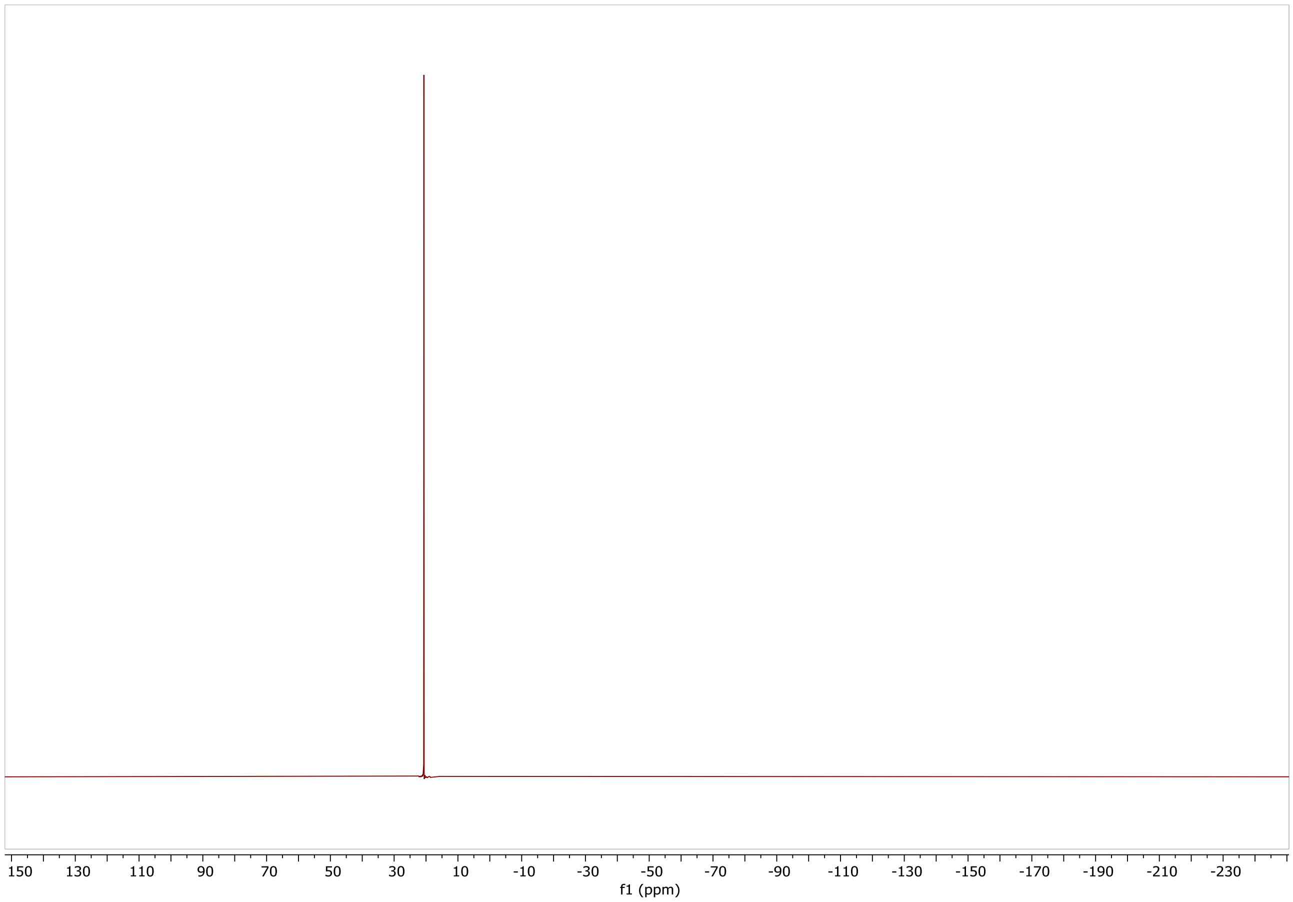

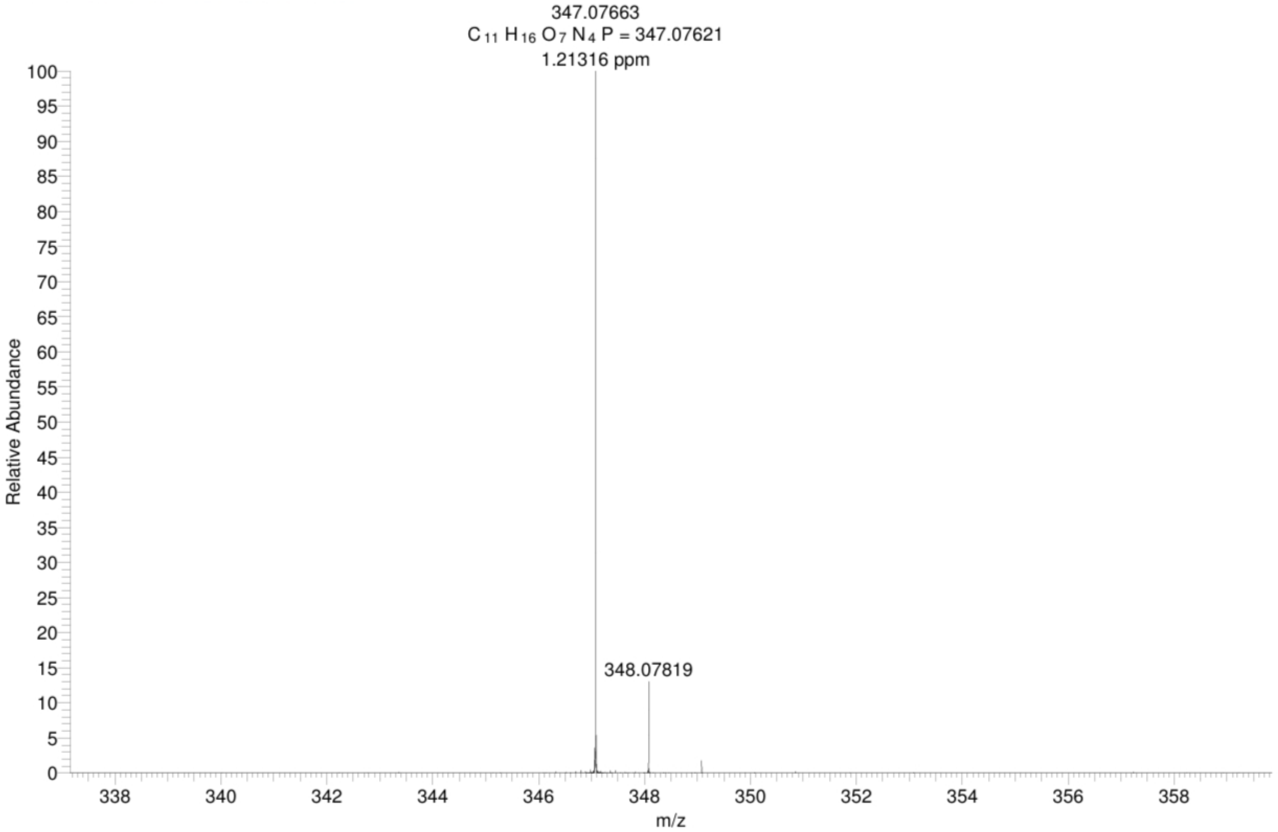

Fig. S14. ^{31}P NMR of compound **20c** (at rt) in D_2O containing 0.1% of *terc*-butyl alcohol as an internal standard (top) and high resolution mass spectrum (HRMS, bottom) of compound **20c**.

Sodium salt of (2-(2-(2-hydroxyethoxy)-2-(guanin-9-yl)ethoxy)ethyl)phosphonic acid (20d)

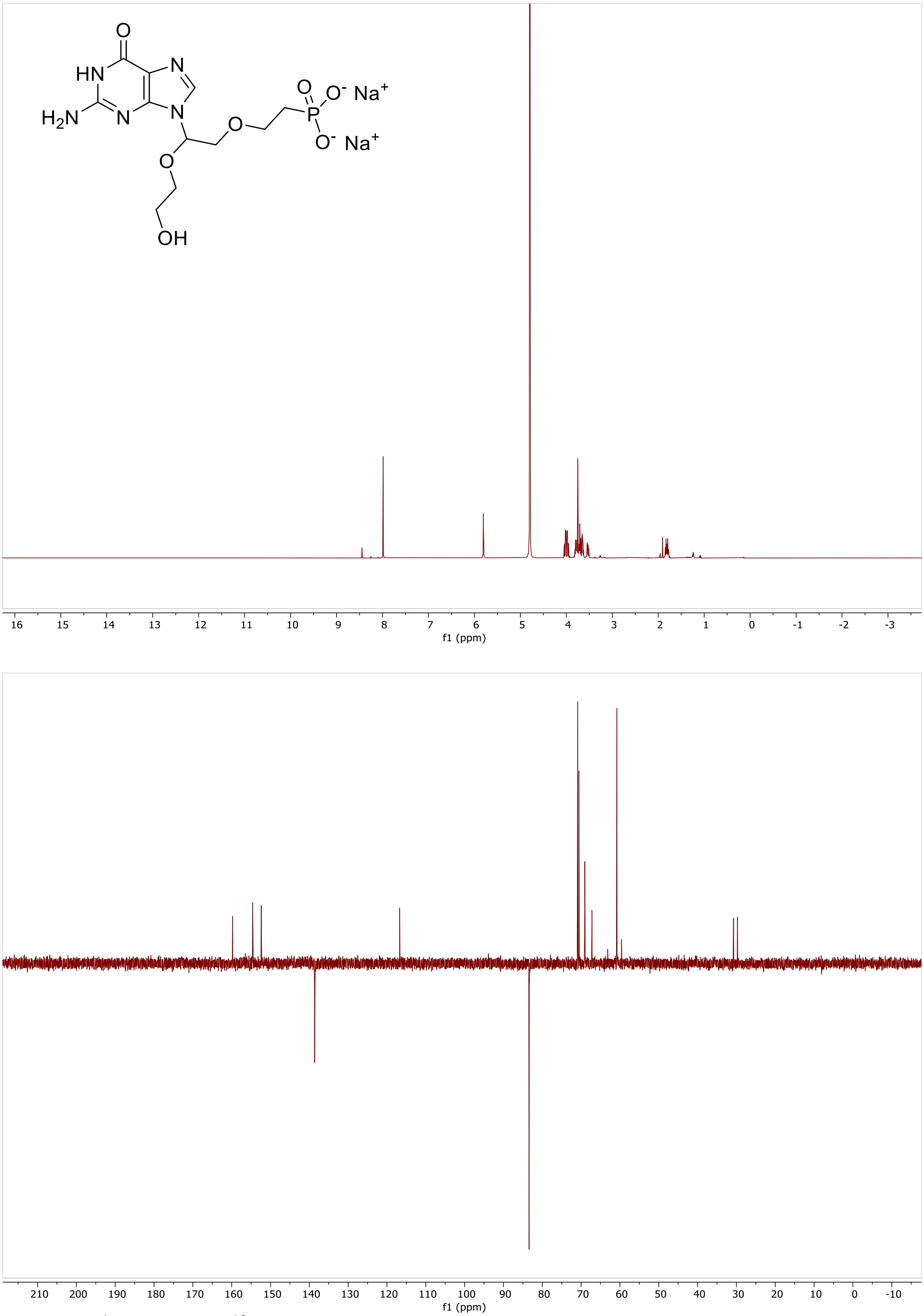

Fig. S15. ^{1}H (top) and ^{13}C (bottom) NMR spectra of compound **20d** (at rt) in D_2O containing 0.1% of dioxane as an internal standard.

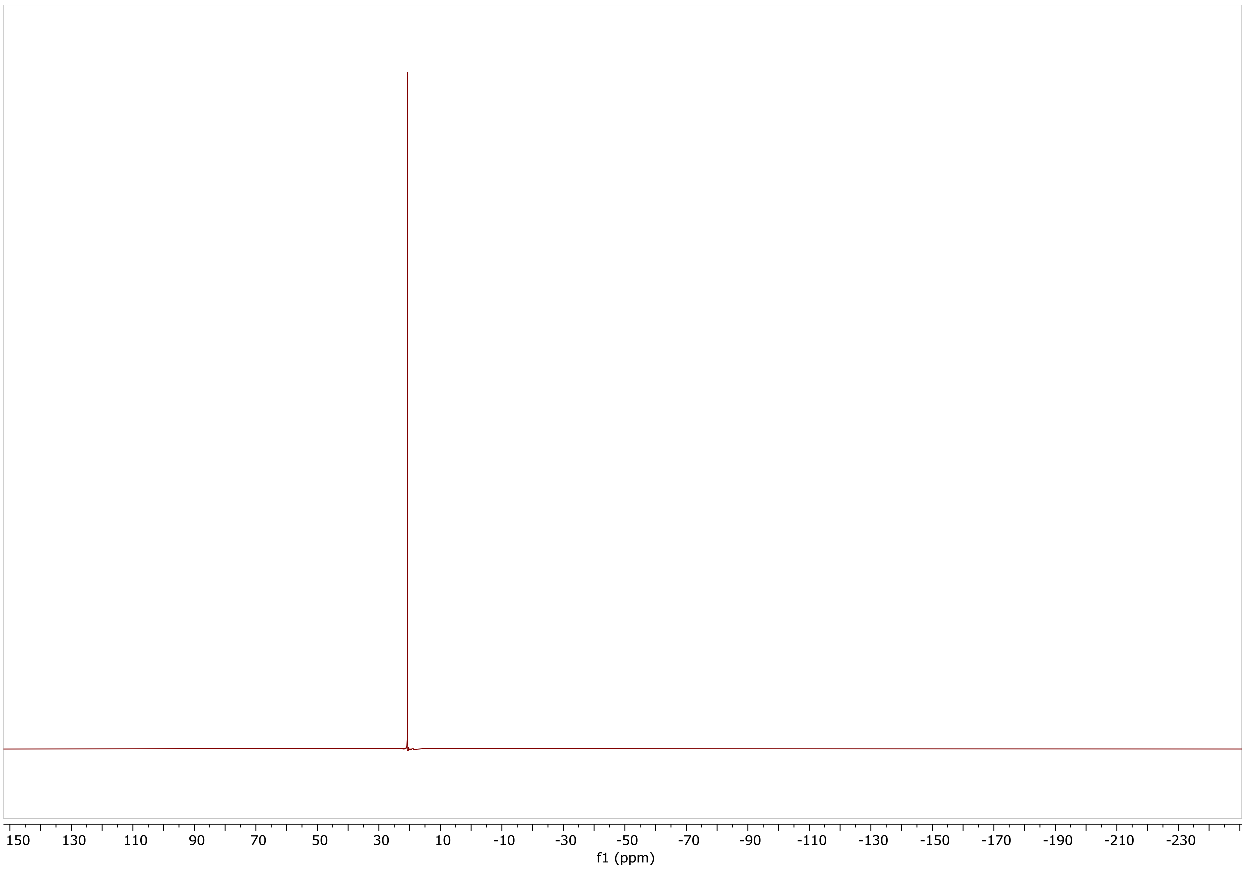

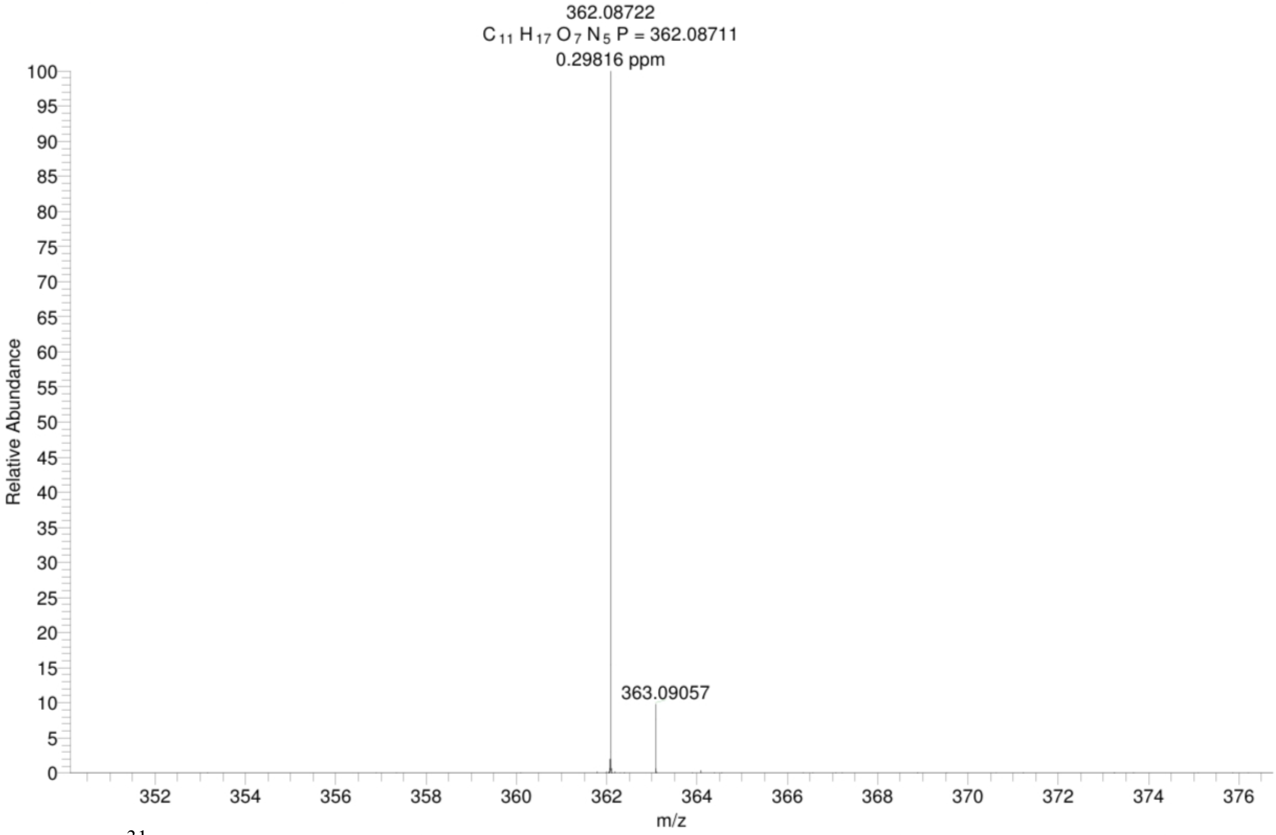

Fig. S16. ^{31}P NMR of compound **20d** (at rt) in D_2O containing 0.1% dioxane as an internal standard (top) and high resolution mass spectrum (HRMS, bottom) of compound **20d**.

Sodium salt of (2-(2-(2-chlorohypoxanthin-9-yl)-2-(2-hydroxyethoxy)ethoxy)ethyl)phosphonic acid (20e)

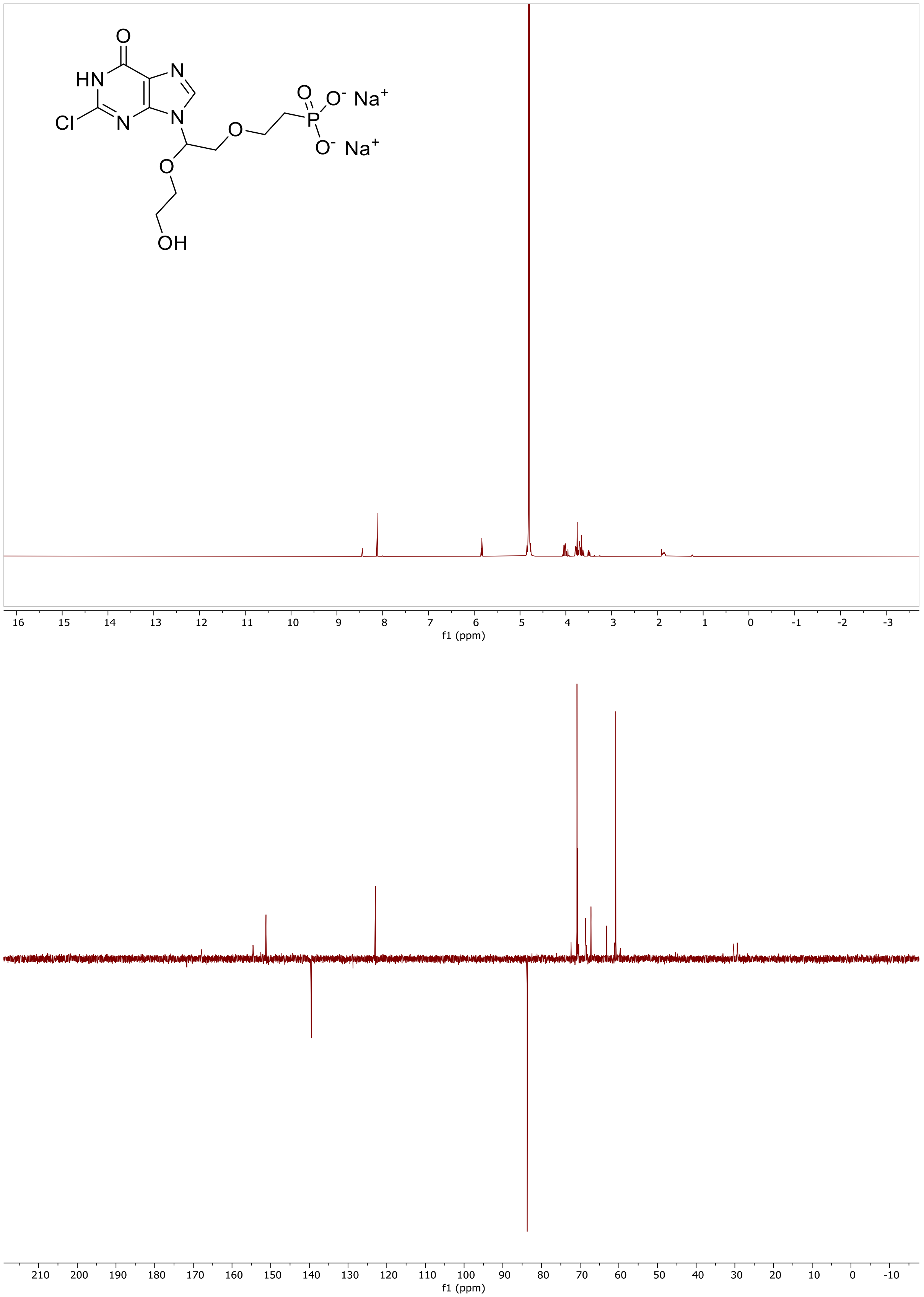

Fig. S17. ^{1}H (top) and ^{13}C (bottom) NMR spectra of compound **20e** (at rt) in D_2O containing 0.1% of dioxane as an internal standard.

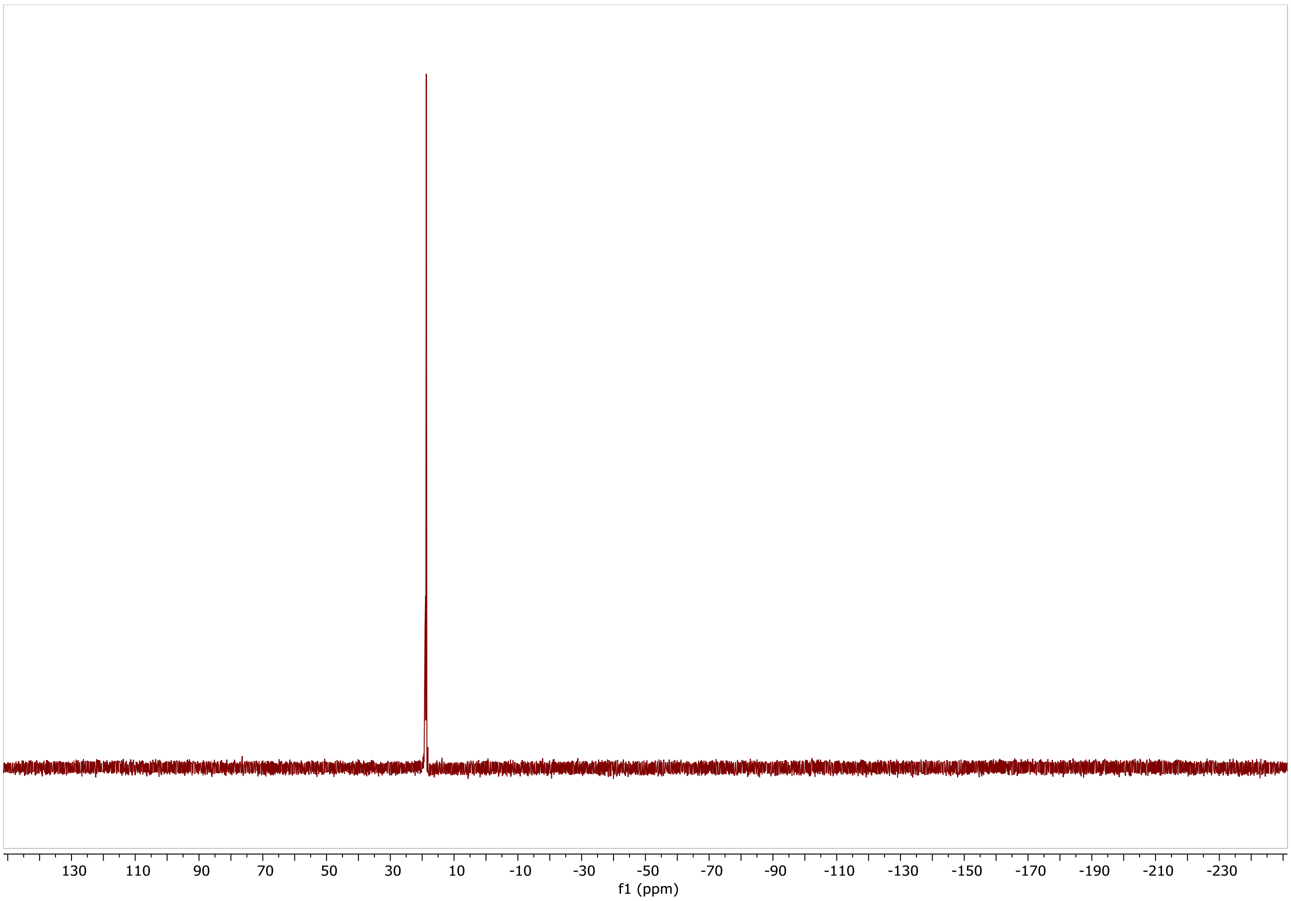

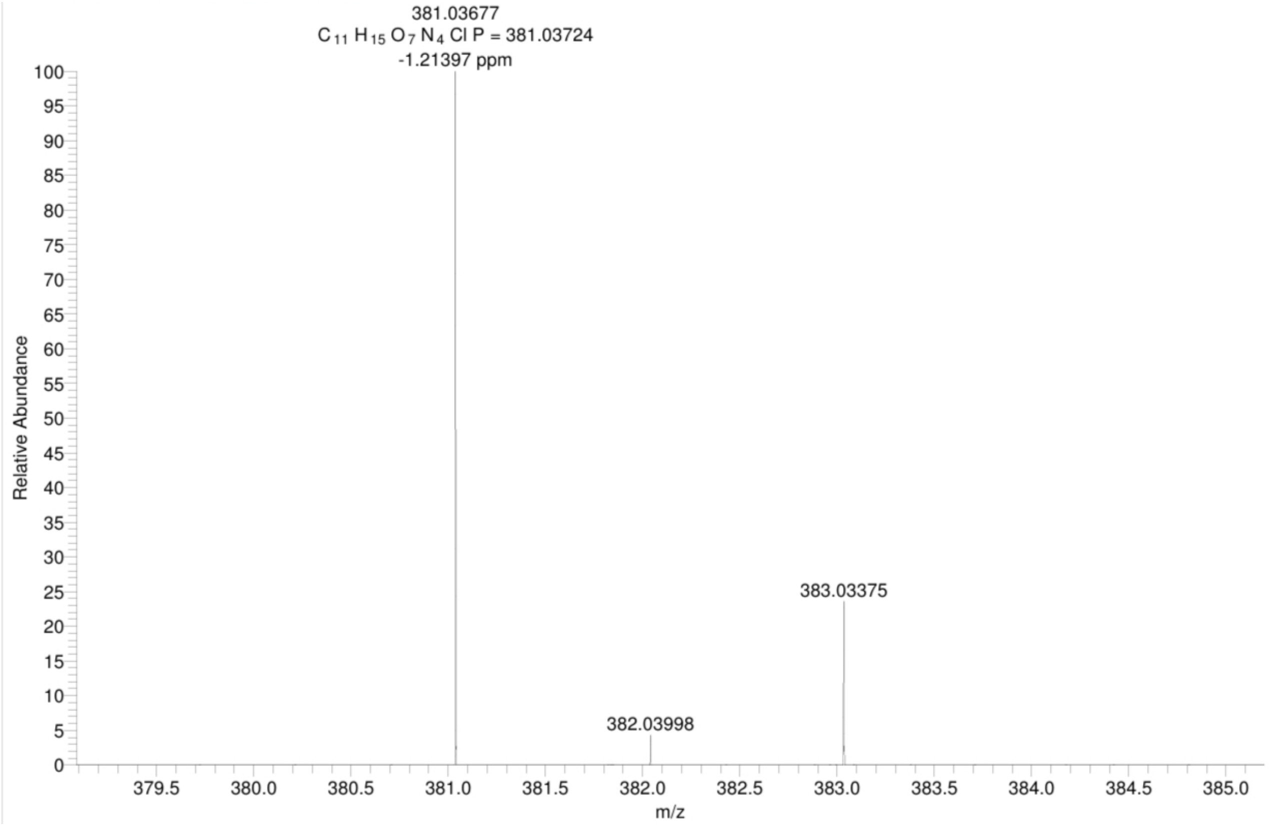

Fig. S18. ^{31}P NMR of compound **20e** (at rt) in D_2O containing 0.1% dioxane as an internal standard (top) and high resolution mass spectrum (HRMS, bottom) of compound **20e**.

Sodium salt of (2-(2-(2-hydroxyethoxy)-2-(4-oxo-4,5-dihydro-1*H*-pyrazolo[3,4-d]pyrimidin-1-yl)ethoxy)ethyl)phosphonic acid (20f)

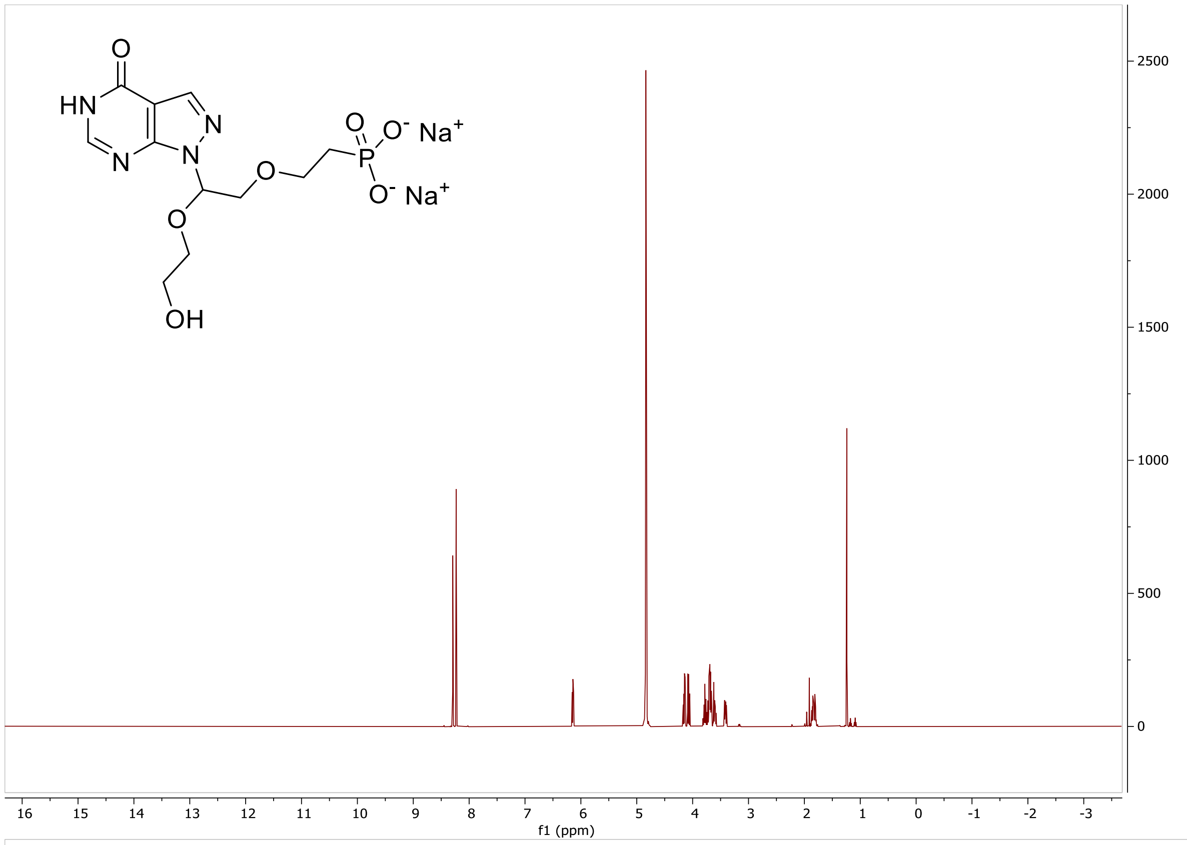

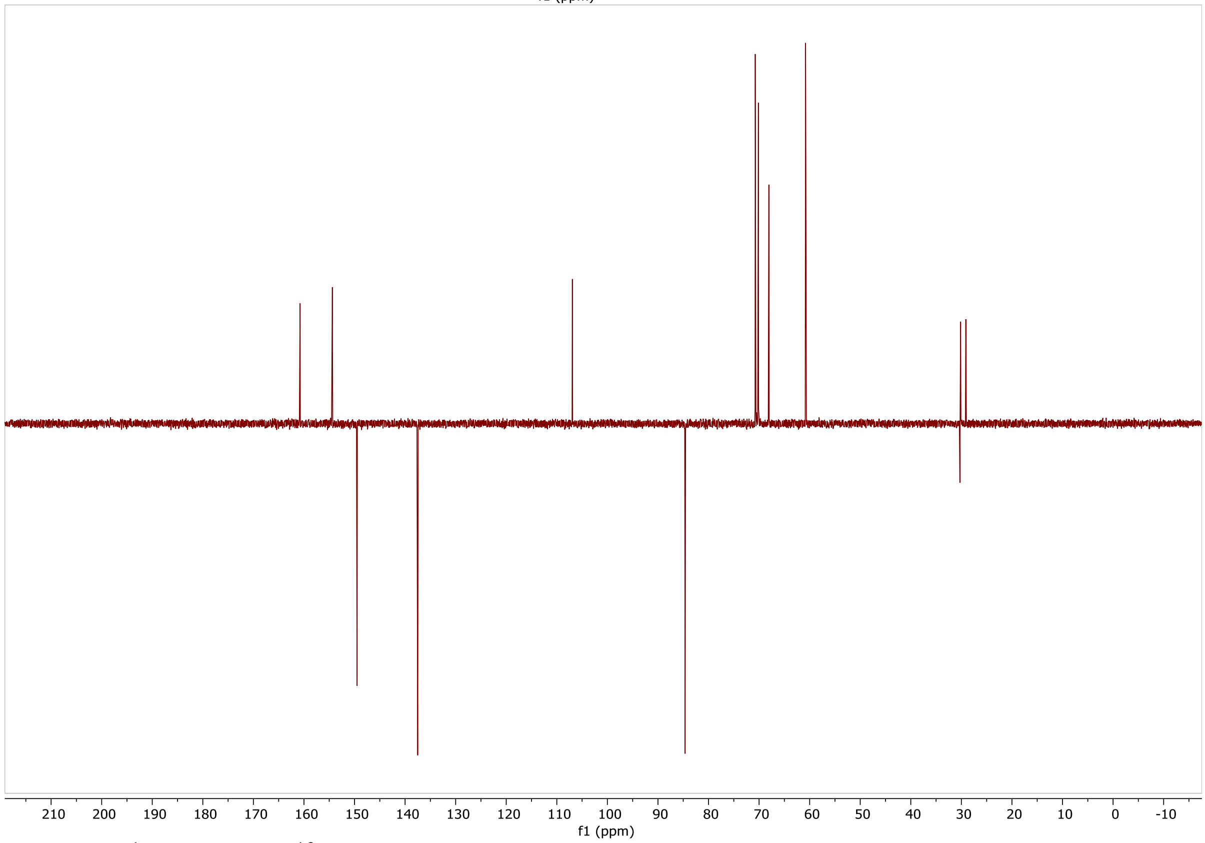

Fig. S19. ^{1}H (top) and ^{13}C (bottom) NMR spectra of compound **20f** (at rt) in D_2O containing 0.1% of *terc*-butyl alcohol as an internal standard.

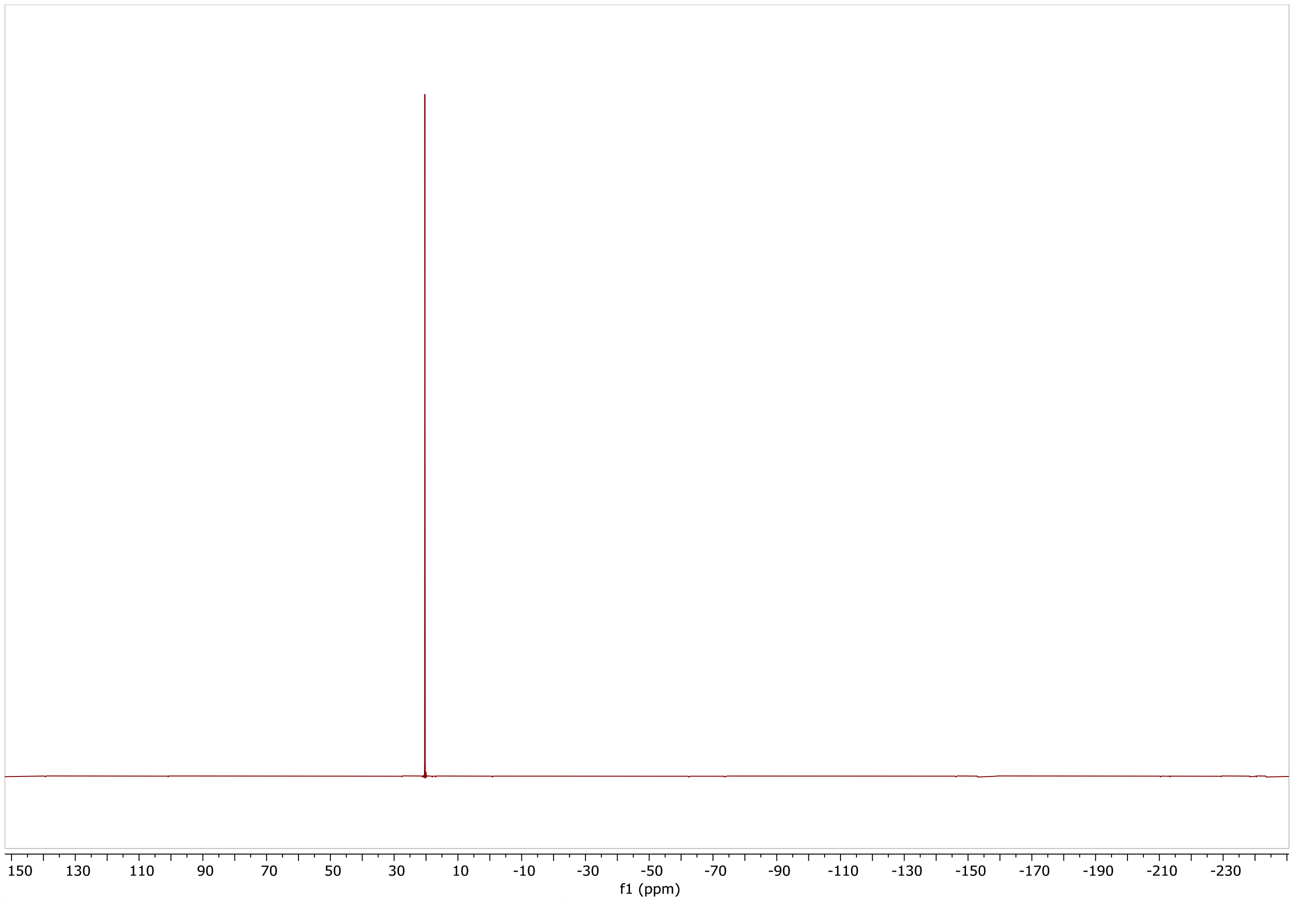

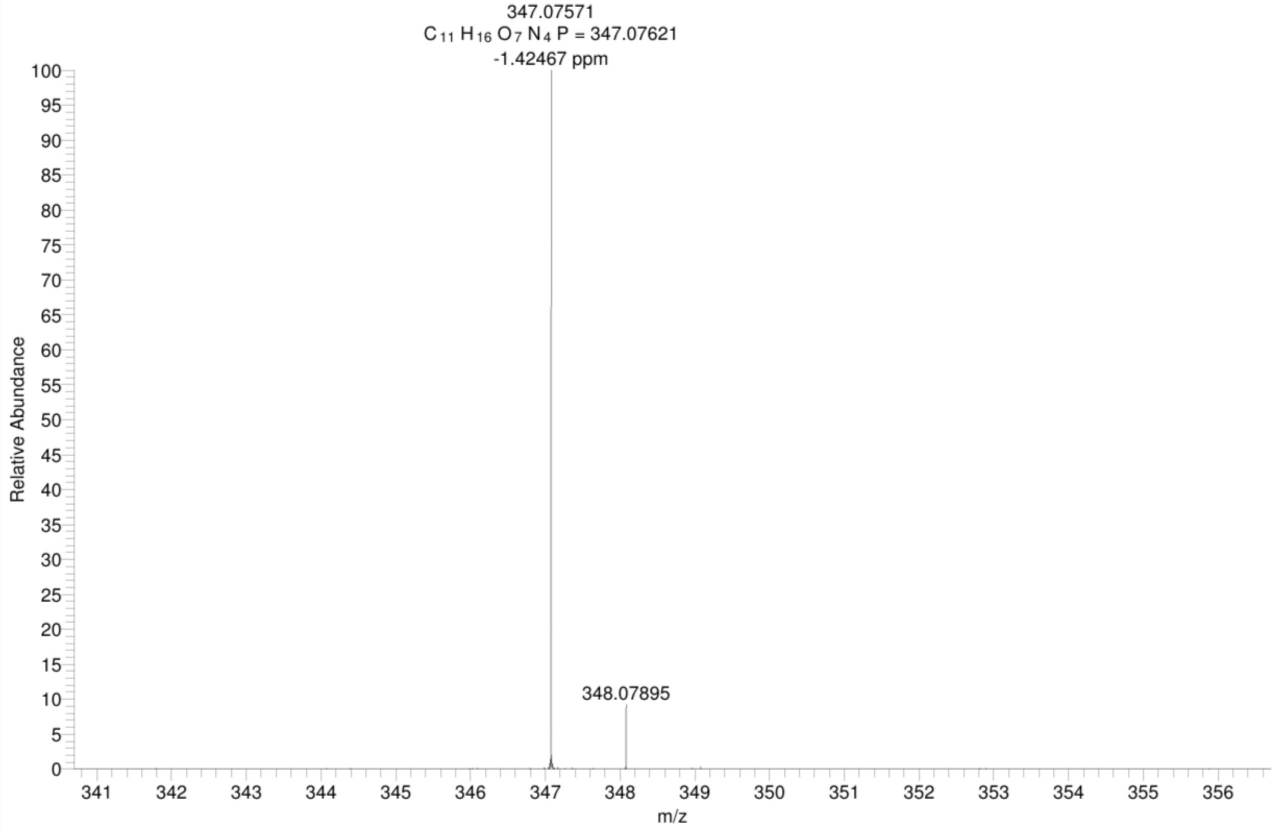

Fig. S20. ^{31}P NMR of compound **20f** (at rt) in D_2O containing 0.1% *terc*-butyl alcohol as internal standard (top) and high resolution mass spectrum (HRMS, bottom) of compound **20f**.

Sodium salt of ((3-(3-hydroxypropoxy)-3-(hypoxanthin-9-yl)propoxy)methyl)phosphonic acid (20g)

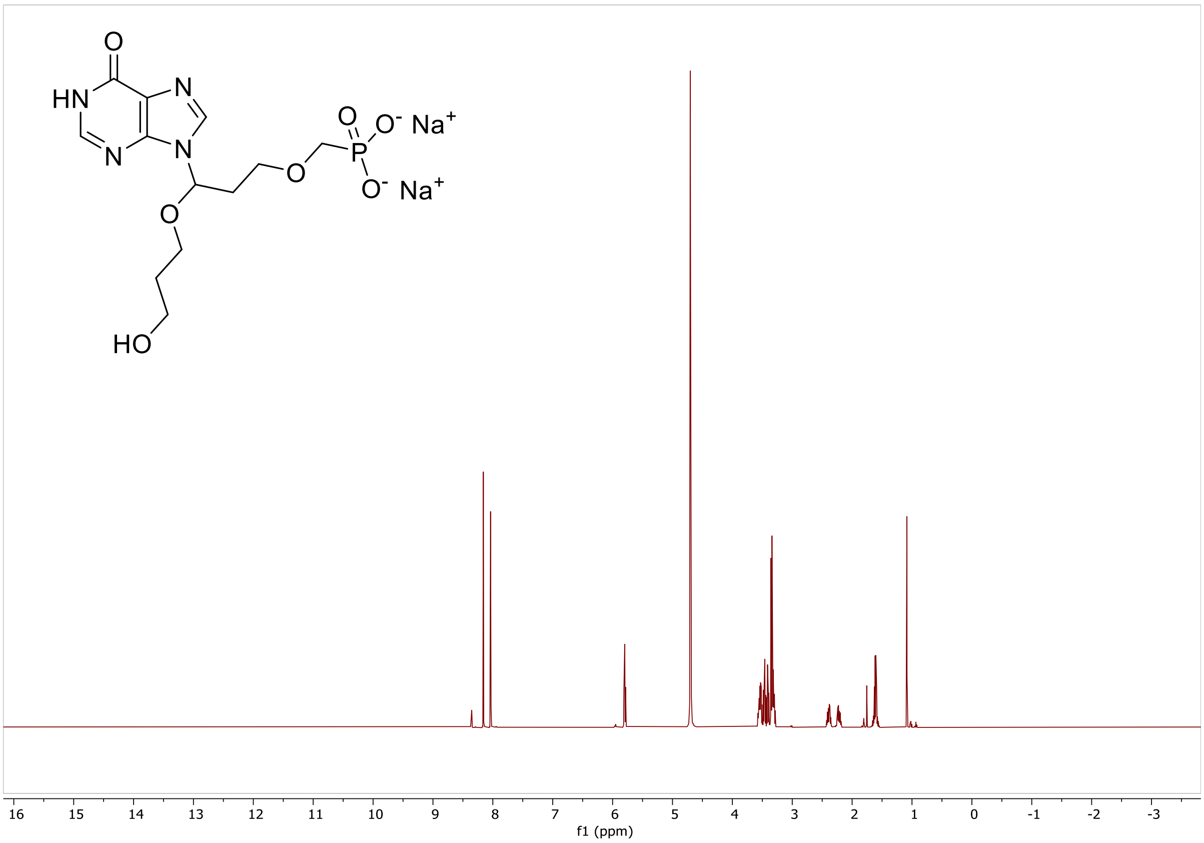

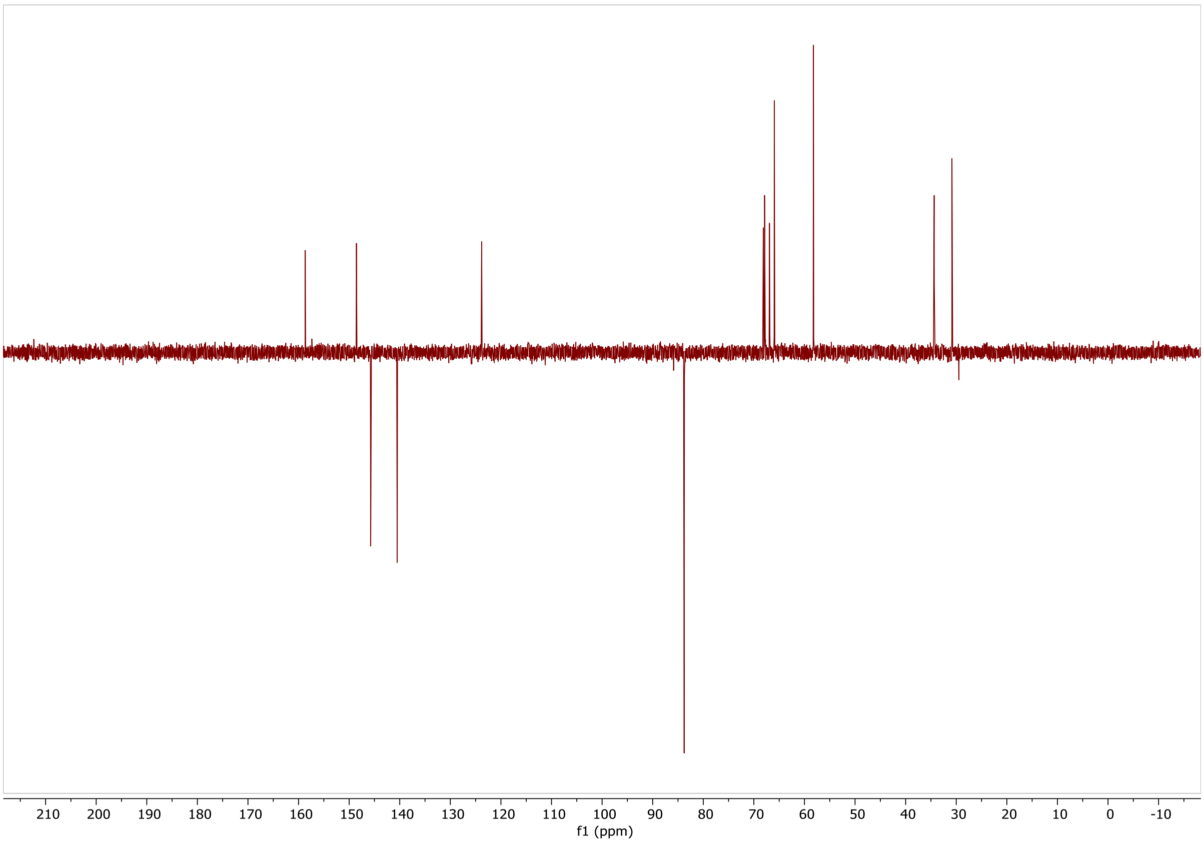

Fig. S21. ^{1}H (top) and ^{13}C (bottom) NMR spectra of compound **20g** (at rt) in D_2O containing 0.1% of *terc*-butyl alcohol as an internal standard.

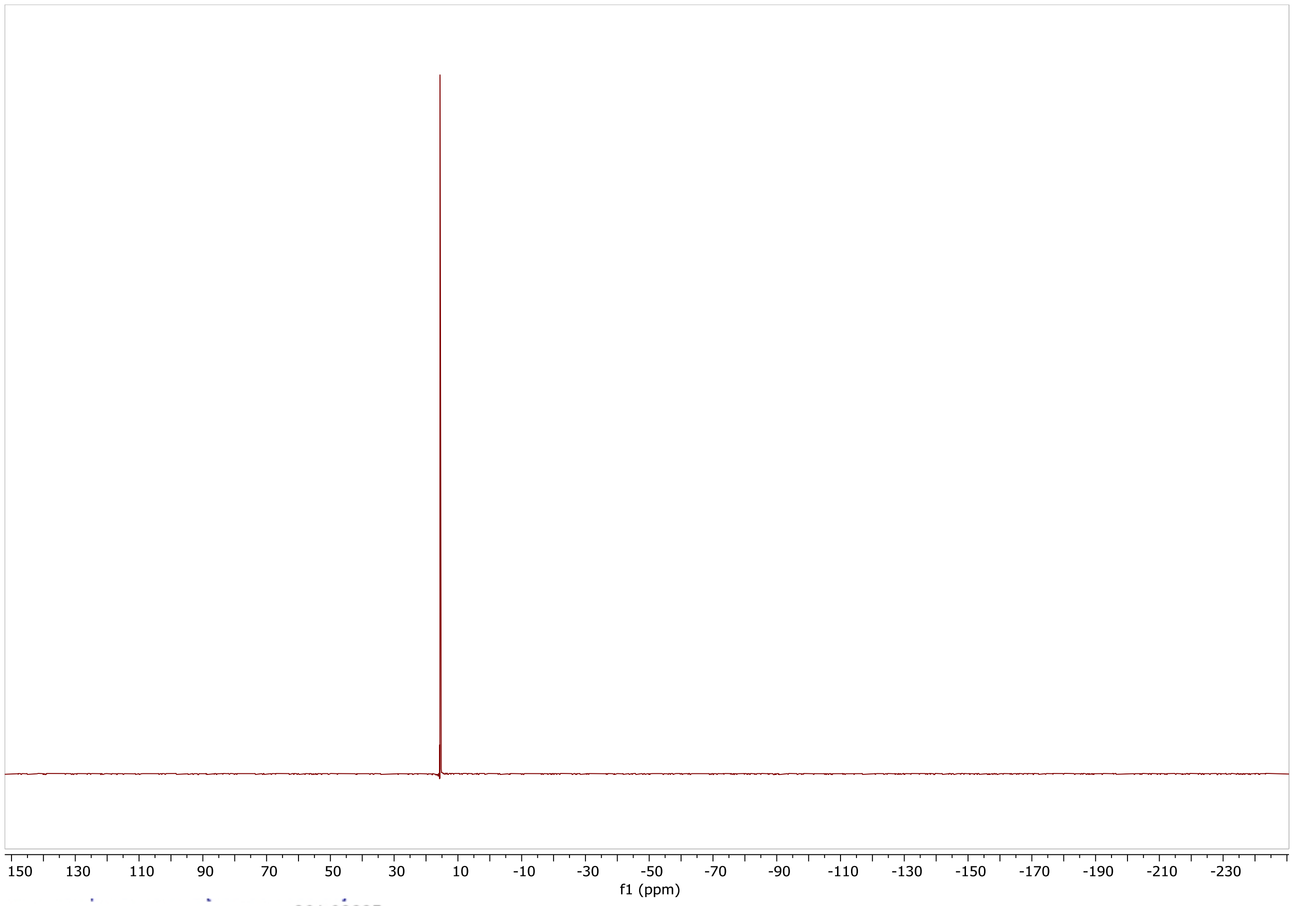

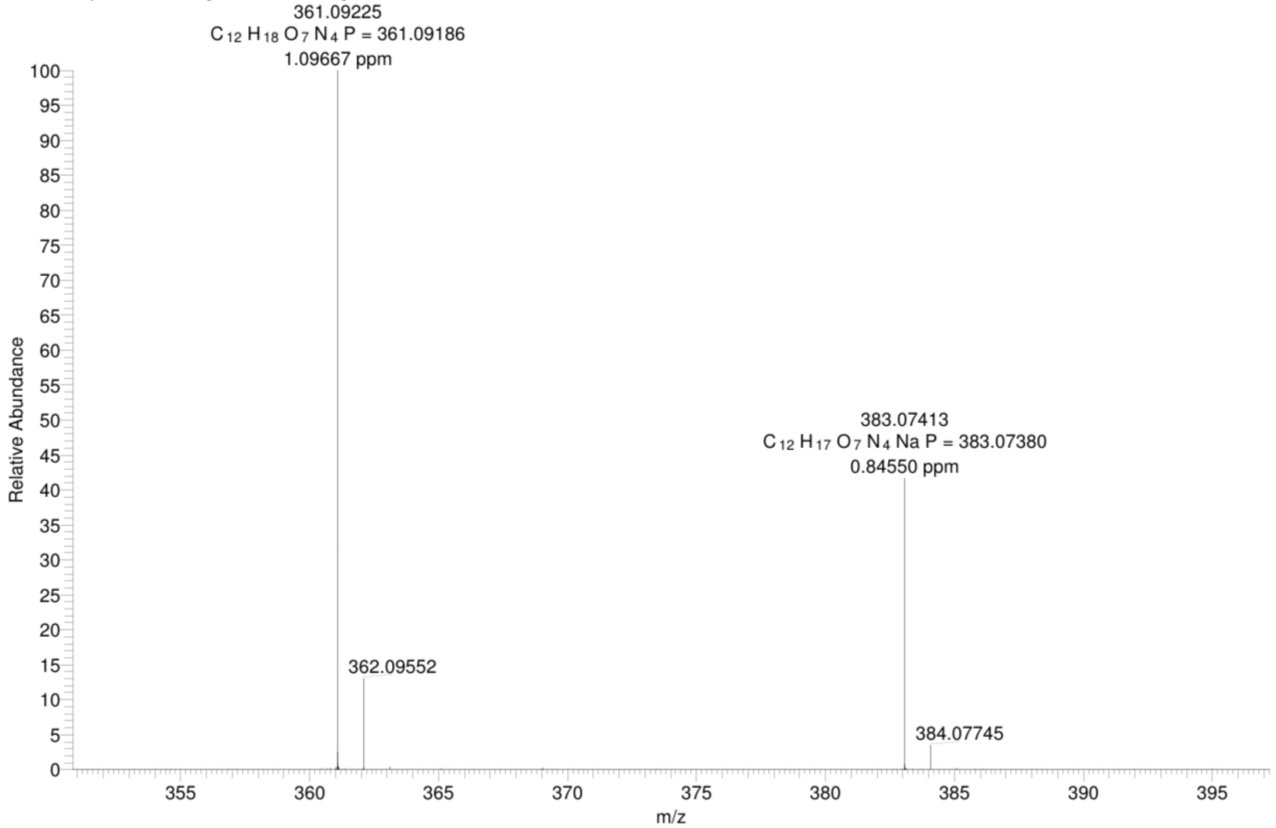

Fig. S22. ^{31}P NMR of compound **20g** (at rt) in D_2O containing 0.1% *terc*-butyl alcohol as an internal standard (top) and high resolution mass spectrum (HRMS, bottom) of compound **20g**.

Sodium salt of ((3-(guanin-9-yl)-3-(3-hydroxypropoxy)propoxy)methyl)phosphonic acid (20h)

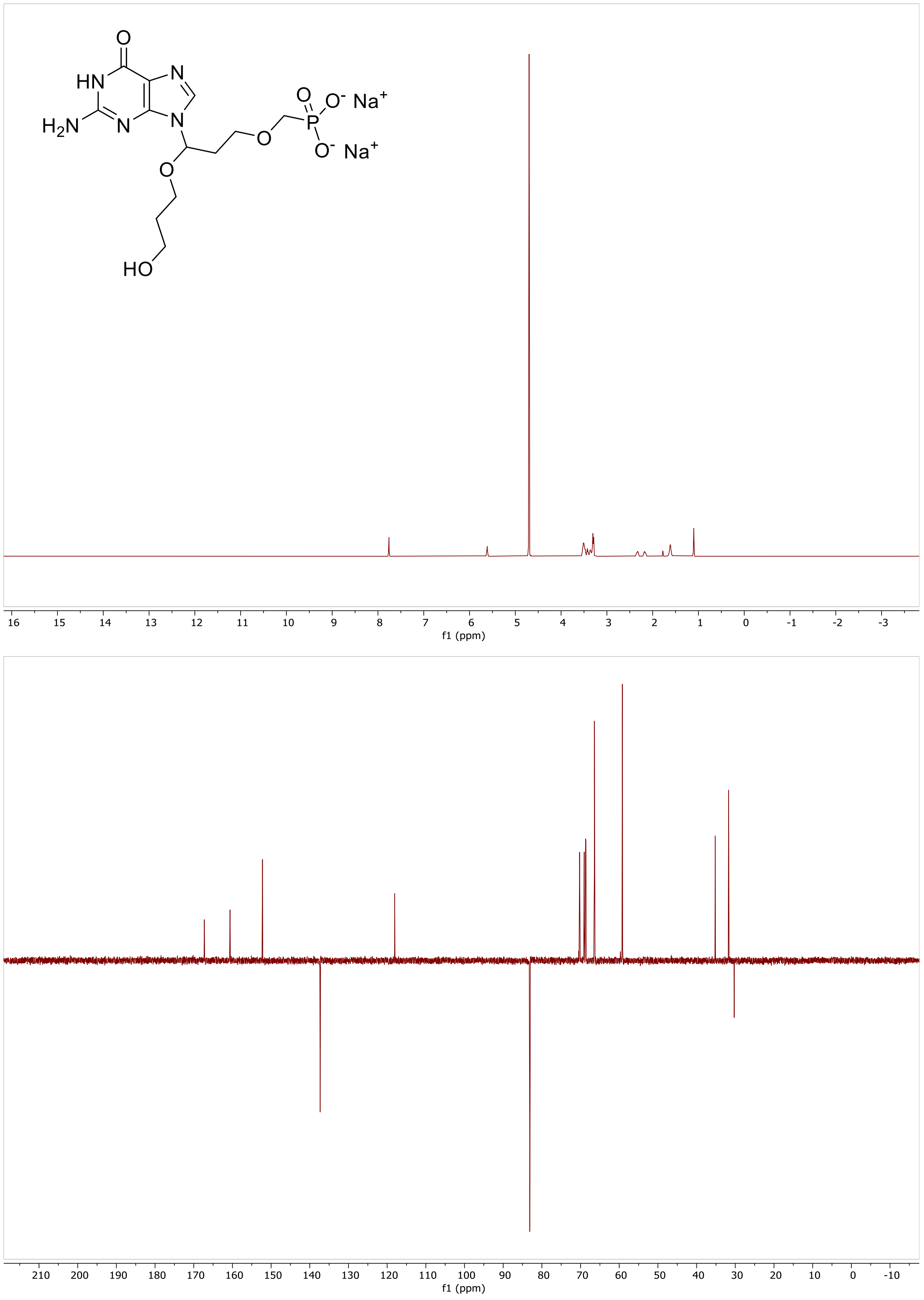

Fig. S23. ^{1}H (top) and ^{13}C (bottom) NMR spectra of compound **20h** (at rt) in D_2O containing 0.1% of *terc*-butyl alcohol as an internal standard.

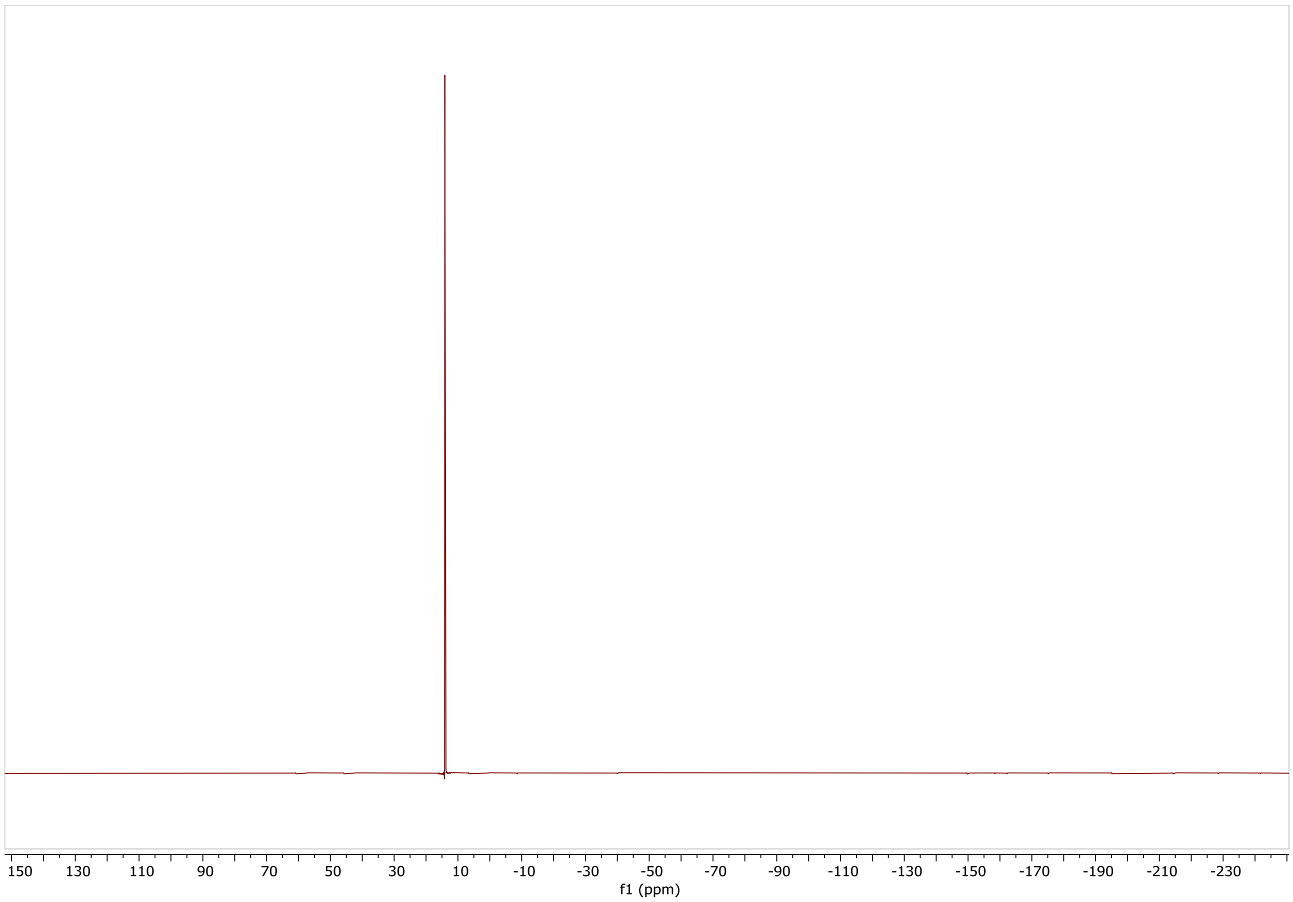

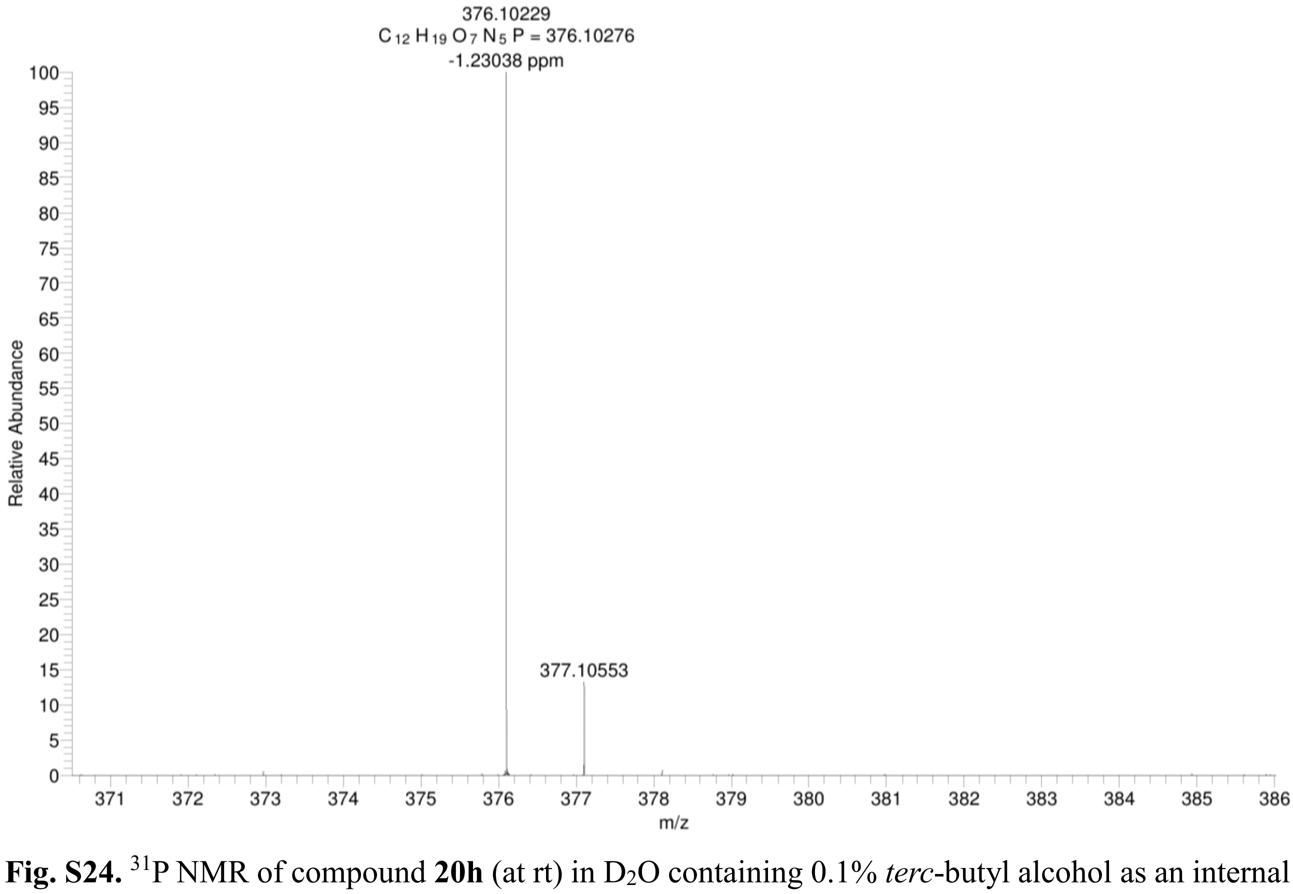

Fig. S24. ^{31}P NMR of compound **20h** (at rt) in D_2O containing 0.1% *terc*-butyl alcohol as an internal standard (top) and high resolution mass spectrum (HRMS, bottom) of compound **20h**.

Sodium salt of ((3-(3-hydroxypropoxy)-3-(4-oxo-4,5-dihydro-1*H*-pyrazolo[3,4-d]pyrimidin-1-yl)propoxy)methyl)phosphonic acid (20i)

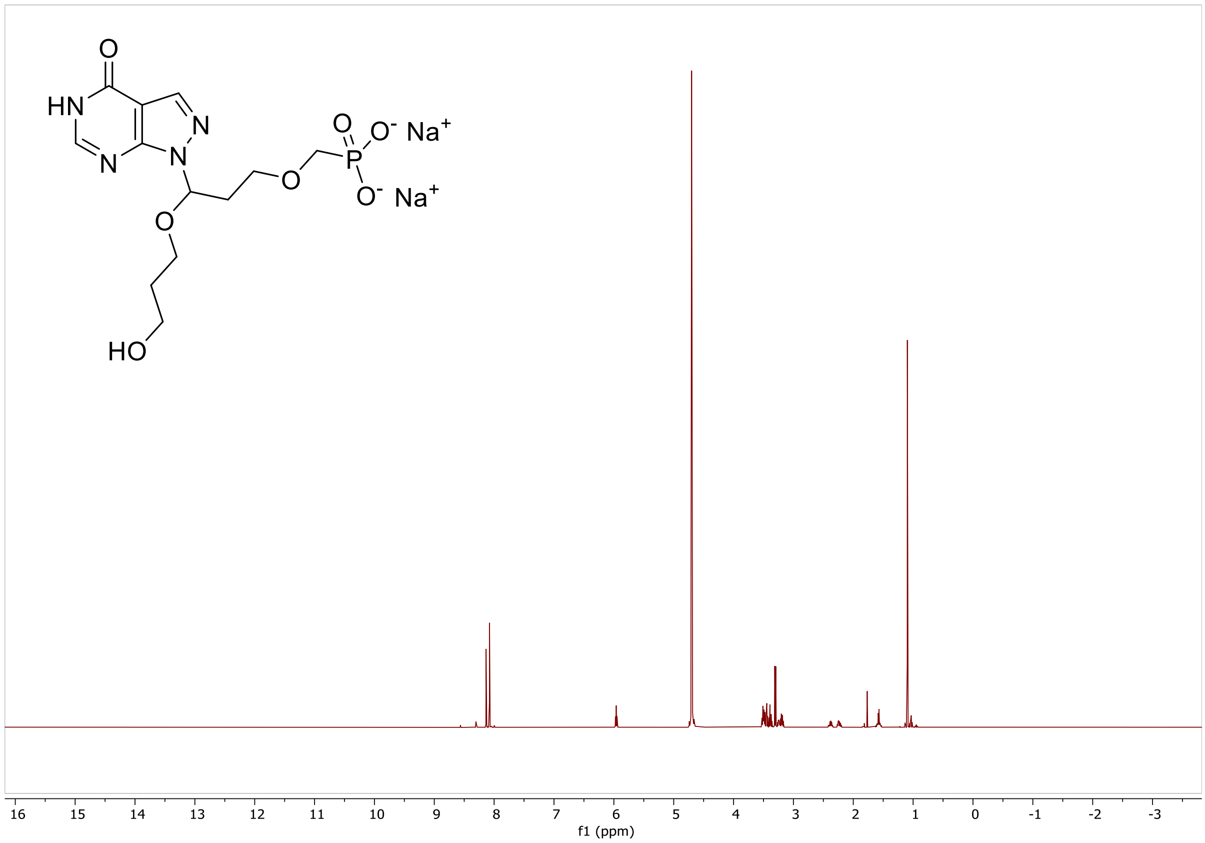

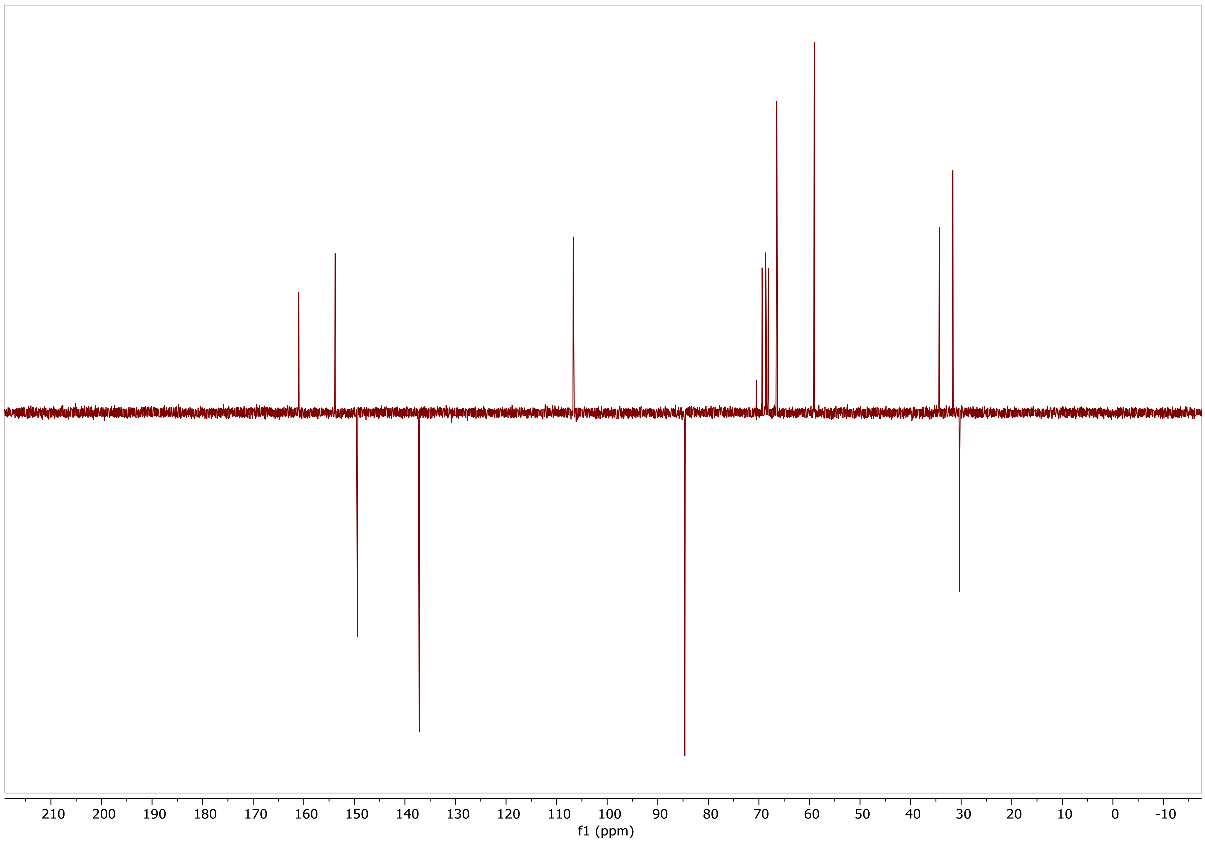

Fig. S25. ^{1}H (top) and ^{13}C (bottom) NMR spectra of compound **20i** (at rt) in D_2O containing 0.1% of *terc*-butyl alcohol as an internal standard.

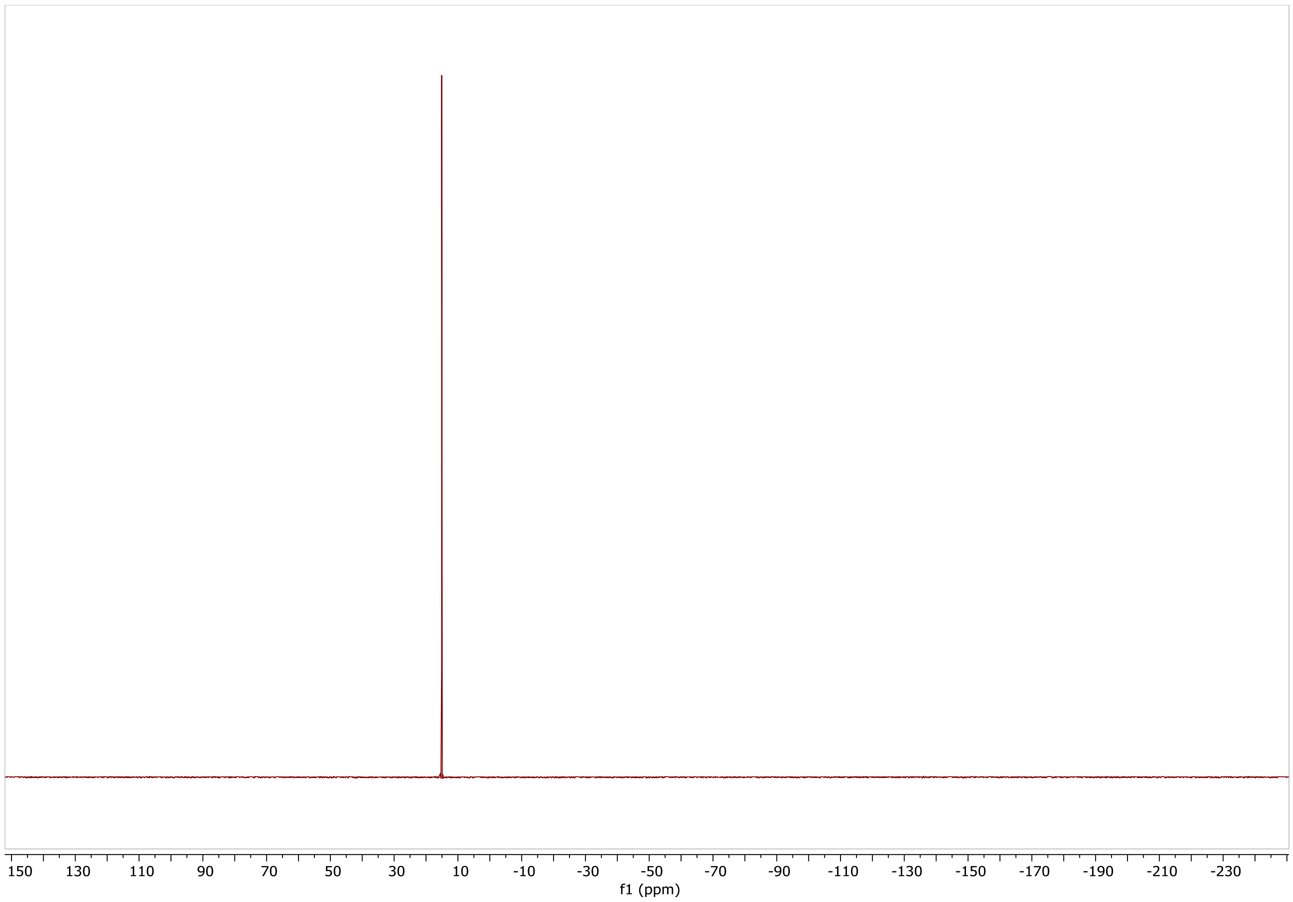

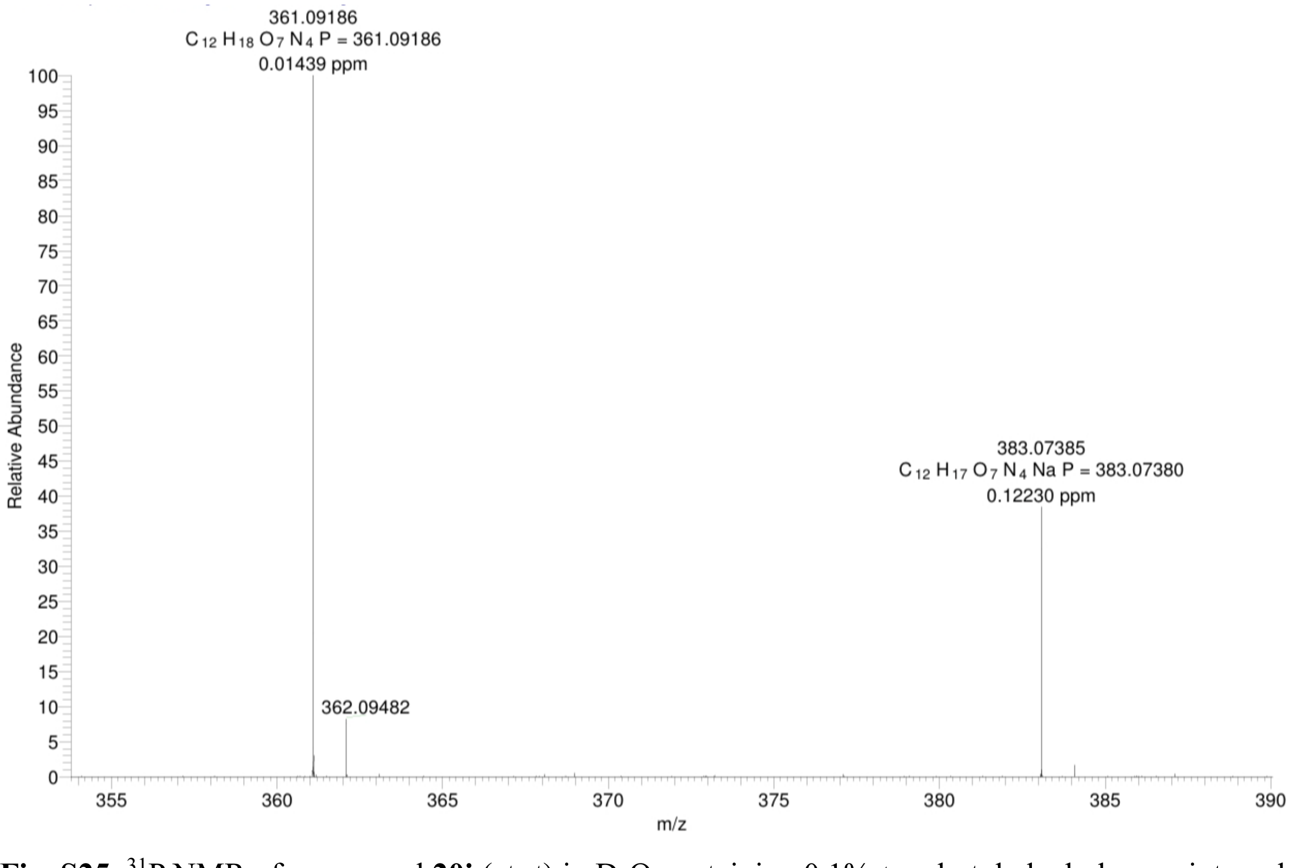

Fig. S25. ^{31}P NMR of compound **20i** (at rt) in D_2O containing 0.1% *terc*-butyl alcohol as an internal standard (top) and high resolution mass spectrum (HRMS, bottom) of compound **20i**.

Bis-(L-phenylalaninate ethyl ester) prodrug of ((2-(2-hydroxyethoxy)-2-(hypoxanthin-9-yl)ethoxy)methyl)phosphonic acid (21a)

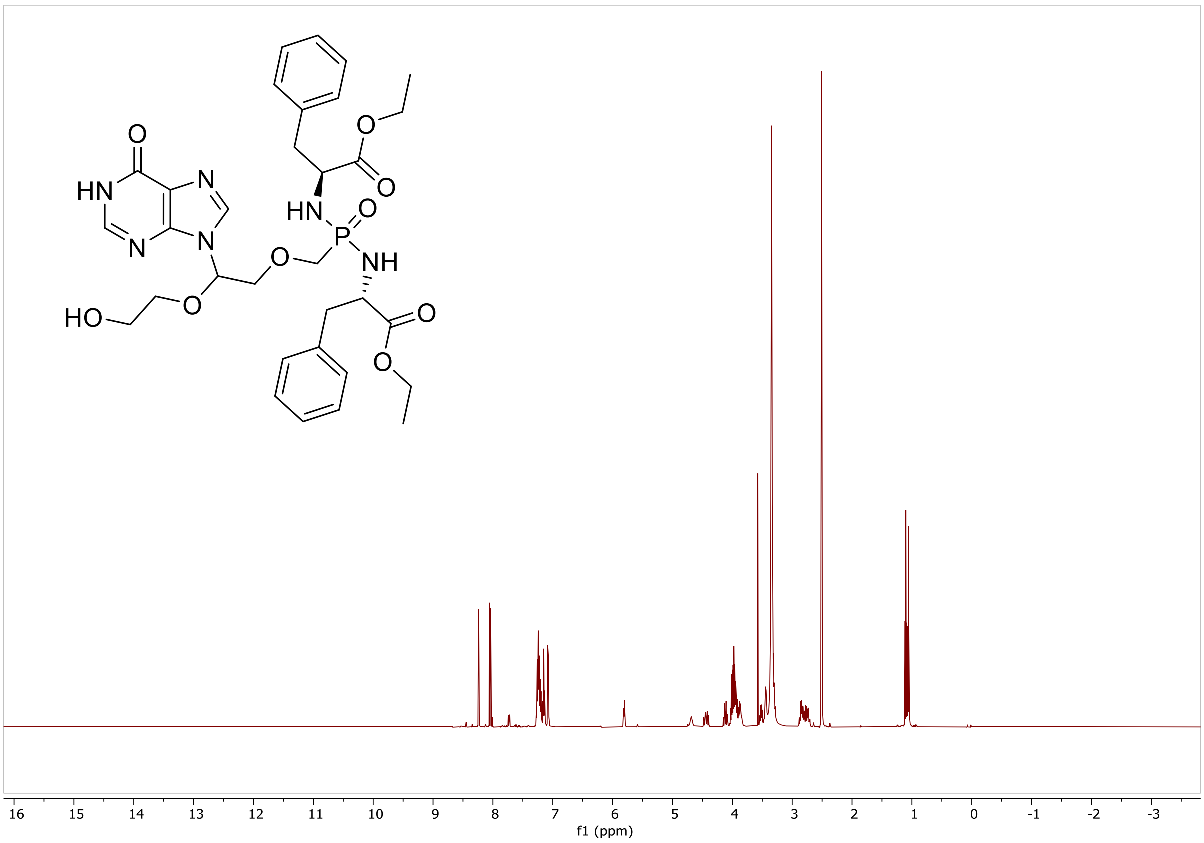

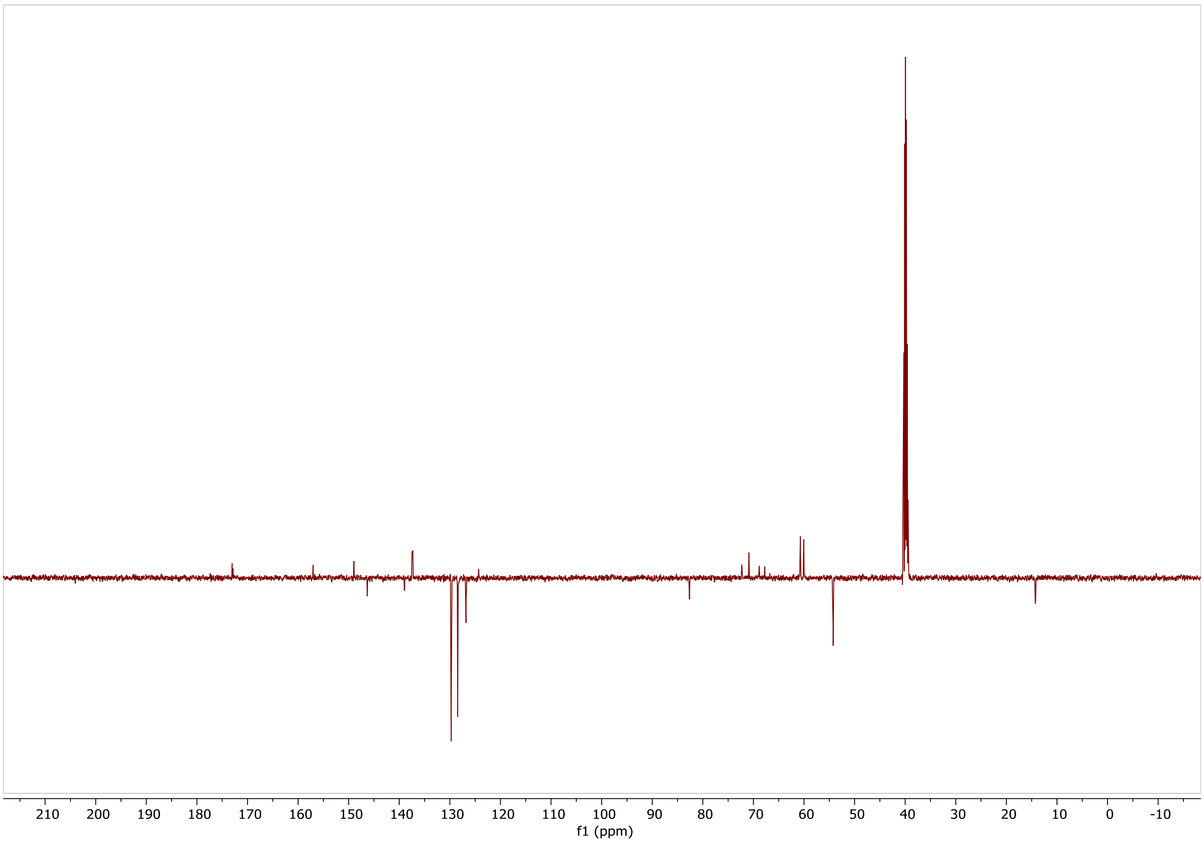

Fig. S26. ^{1}H (top) and ^{13}C (bottom) NMR spectra of compound **21a** (at rt) in DMSO.

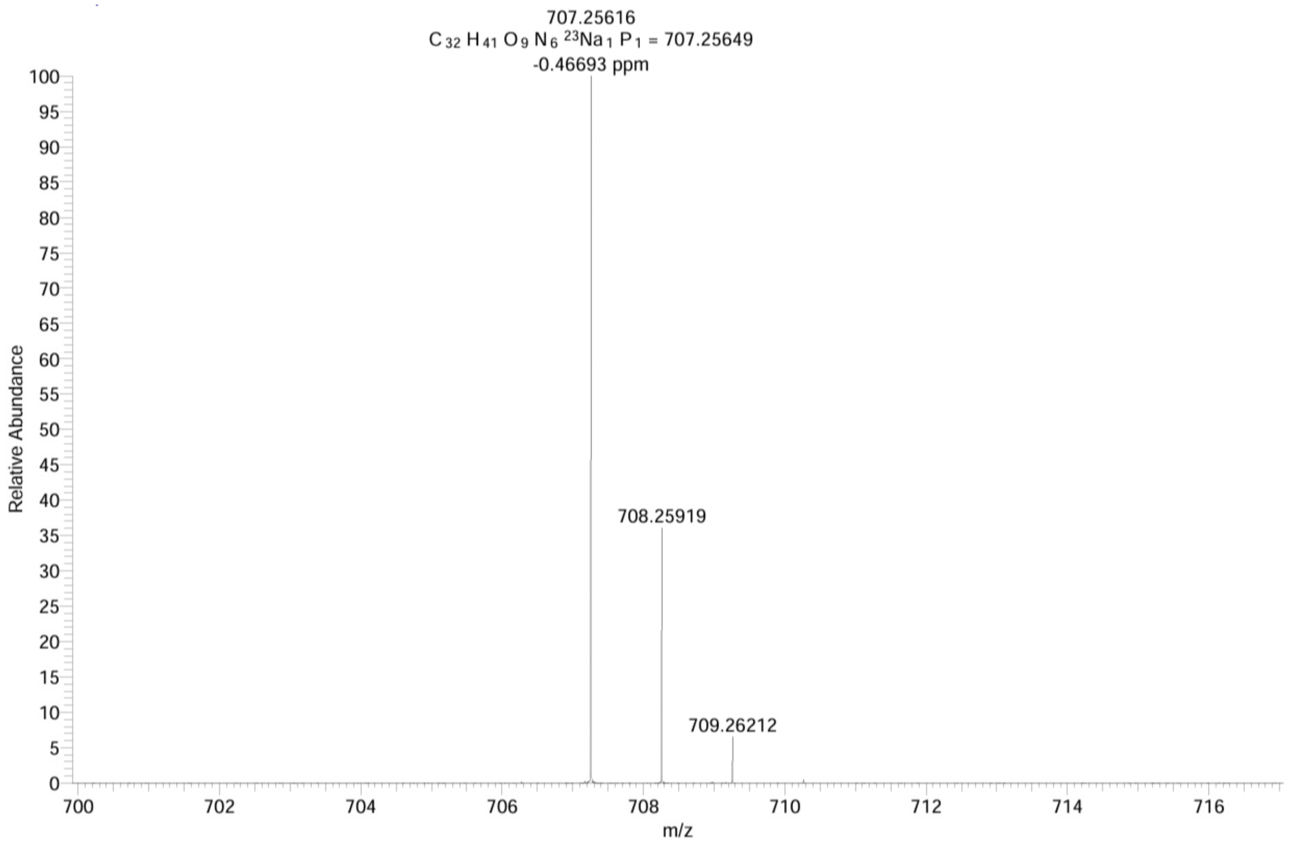

Fig. S27. High resolution mass spectrum (HRMS) of compound **21a**.

Bis-(L-phenylalaninate ethyl ester) prodrug of ((2-(guanin-9-yl)-2-(2-hydroxyethoxy)ethoxy)methyl)phosphonic acid (21b)

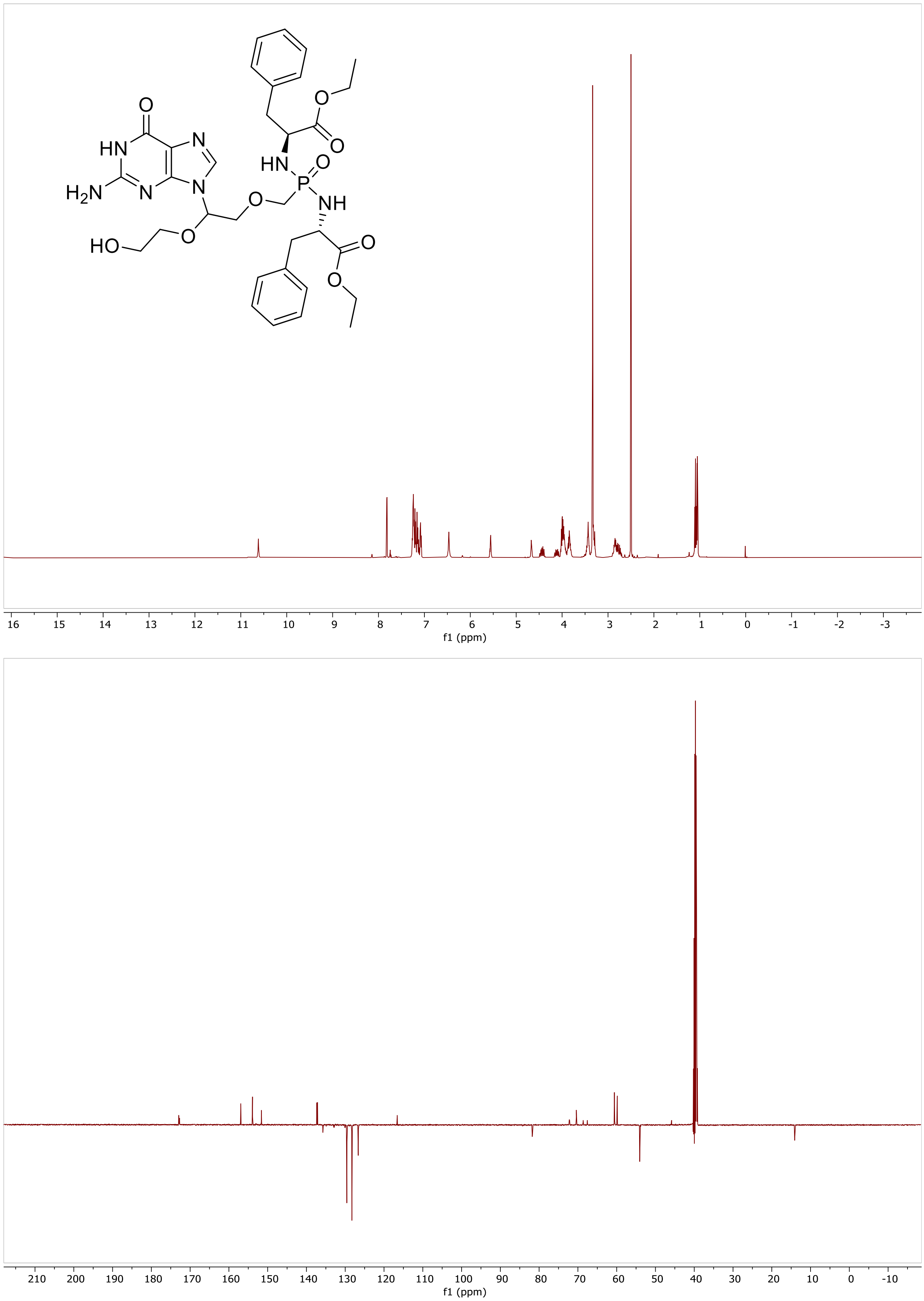

Fig. S28. ^{1}H (top) and ^{13}C (bottom) NMR spectra of compound **21b** (at rt) in DMSO.

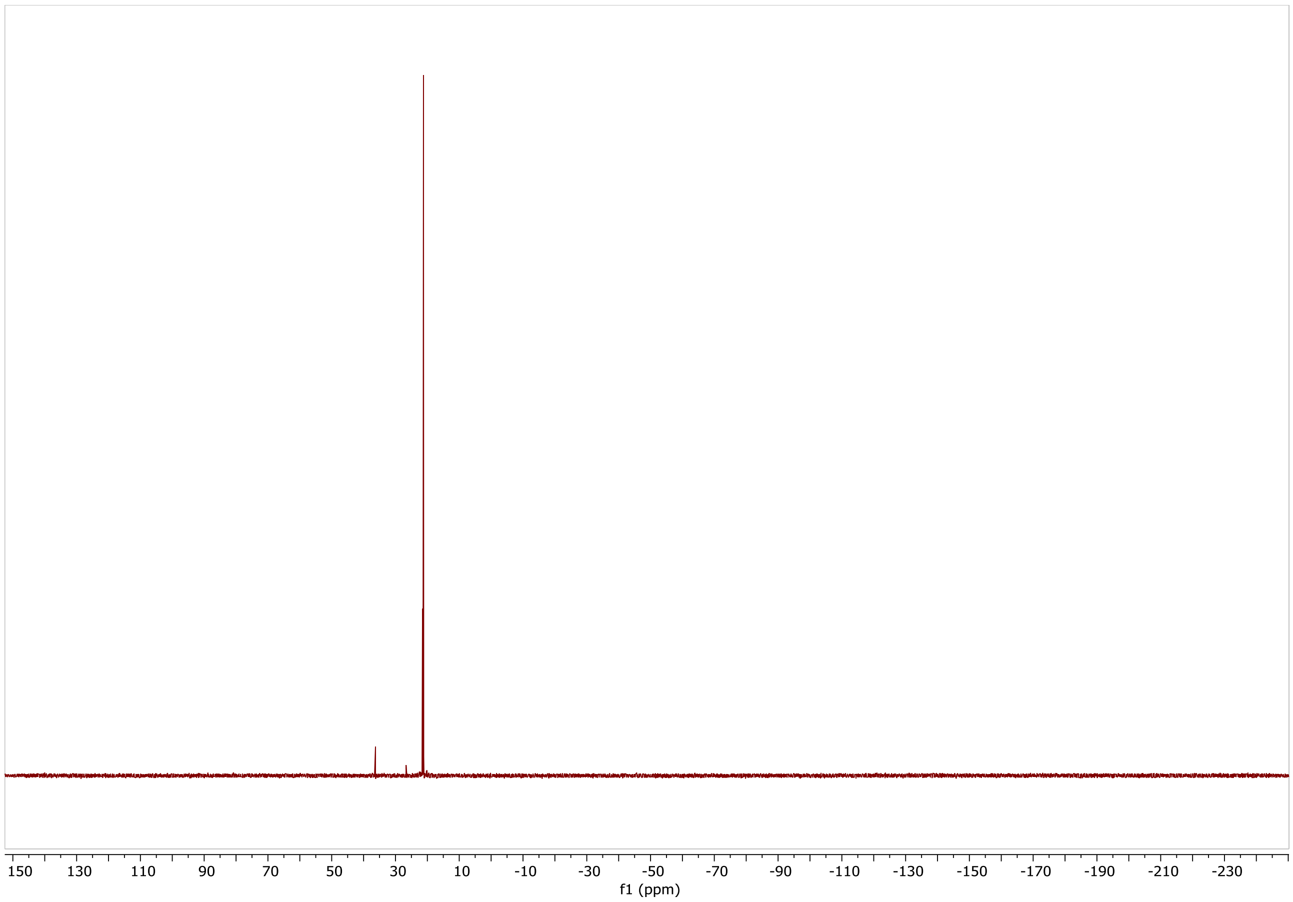

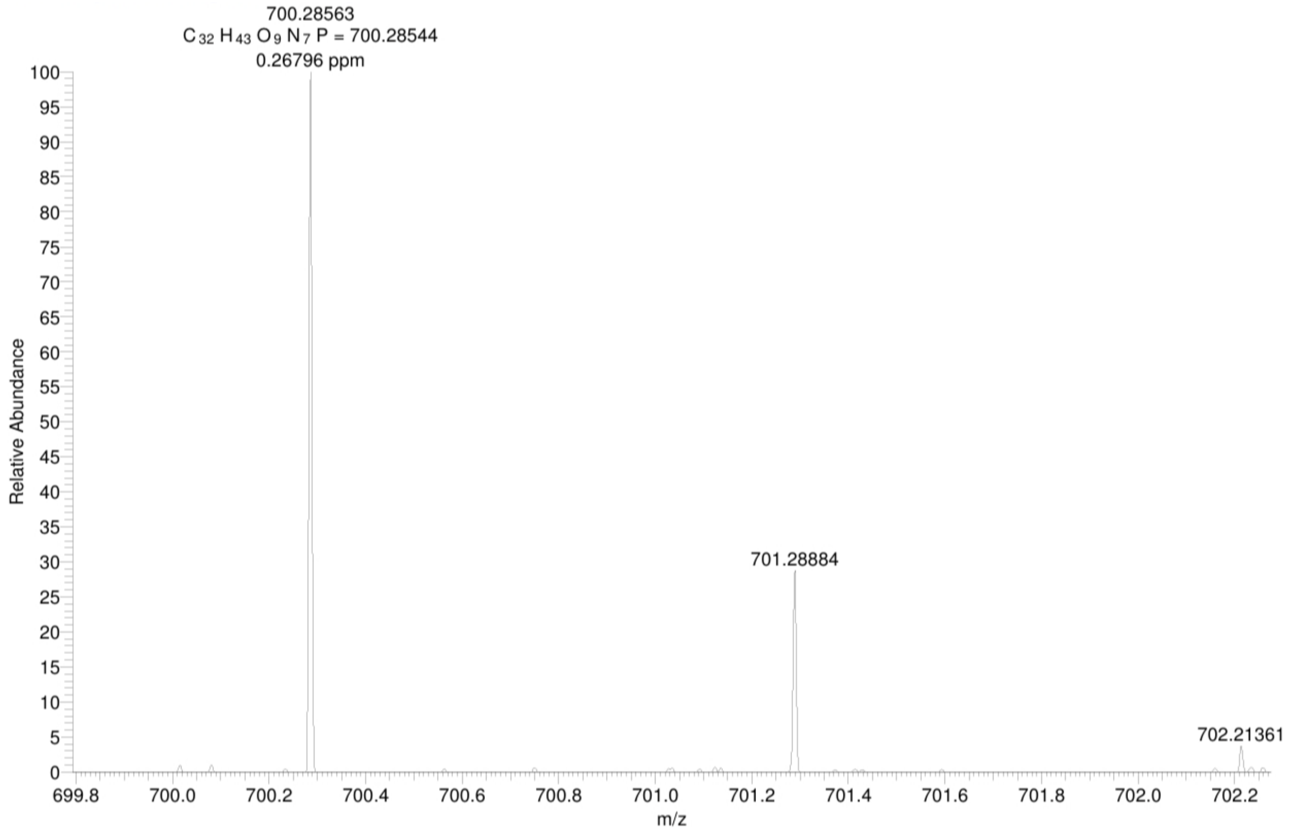

Fig. S29. ^{31}P NMR of compound **21b** (at rt) in DMSO (top) and high resolution mass spectrum (HRMS, bottom) of compound **21b**.

Bis-(L-phenylalaninate ethyl ester) prodrug of (2-(2-(2-hydroxyethoxy)-2-(hypoxanthin-9-yl)ethoxy)ethyl) phosphonic acid (21c)

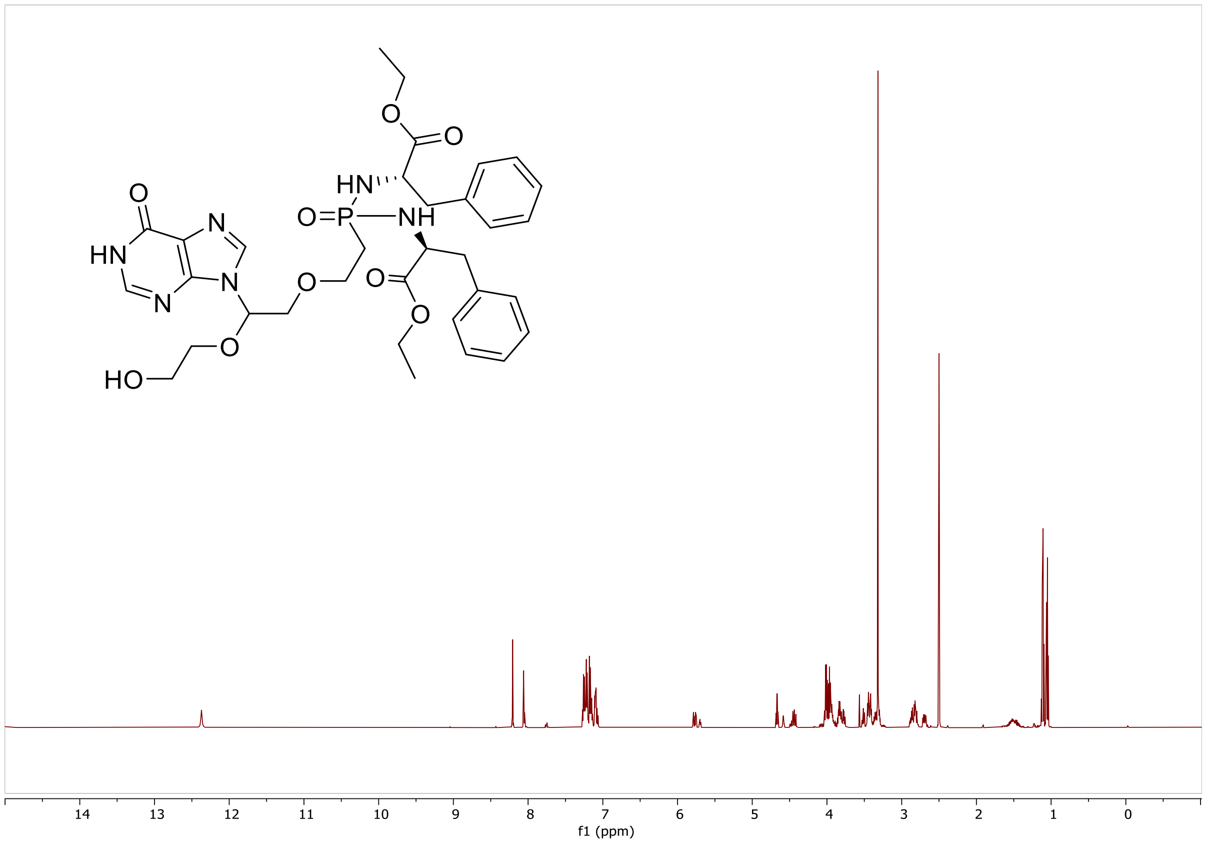

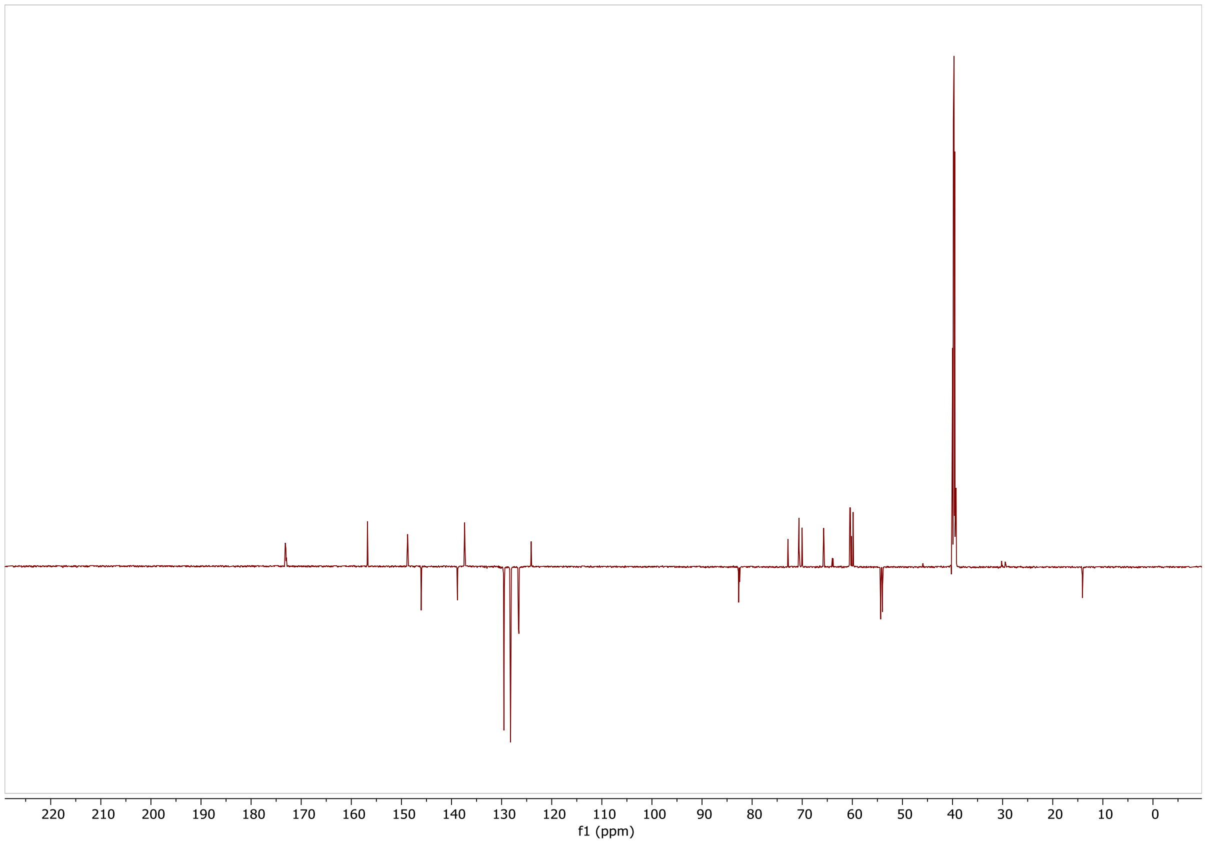

Fig. S30. ^{1}H (top) and ^{13}C (bottom) NMR spectra of compound **21c** (at rt) in DMSO.

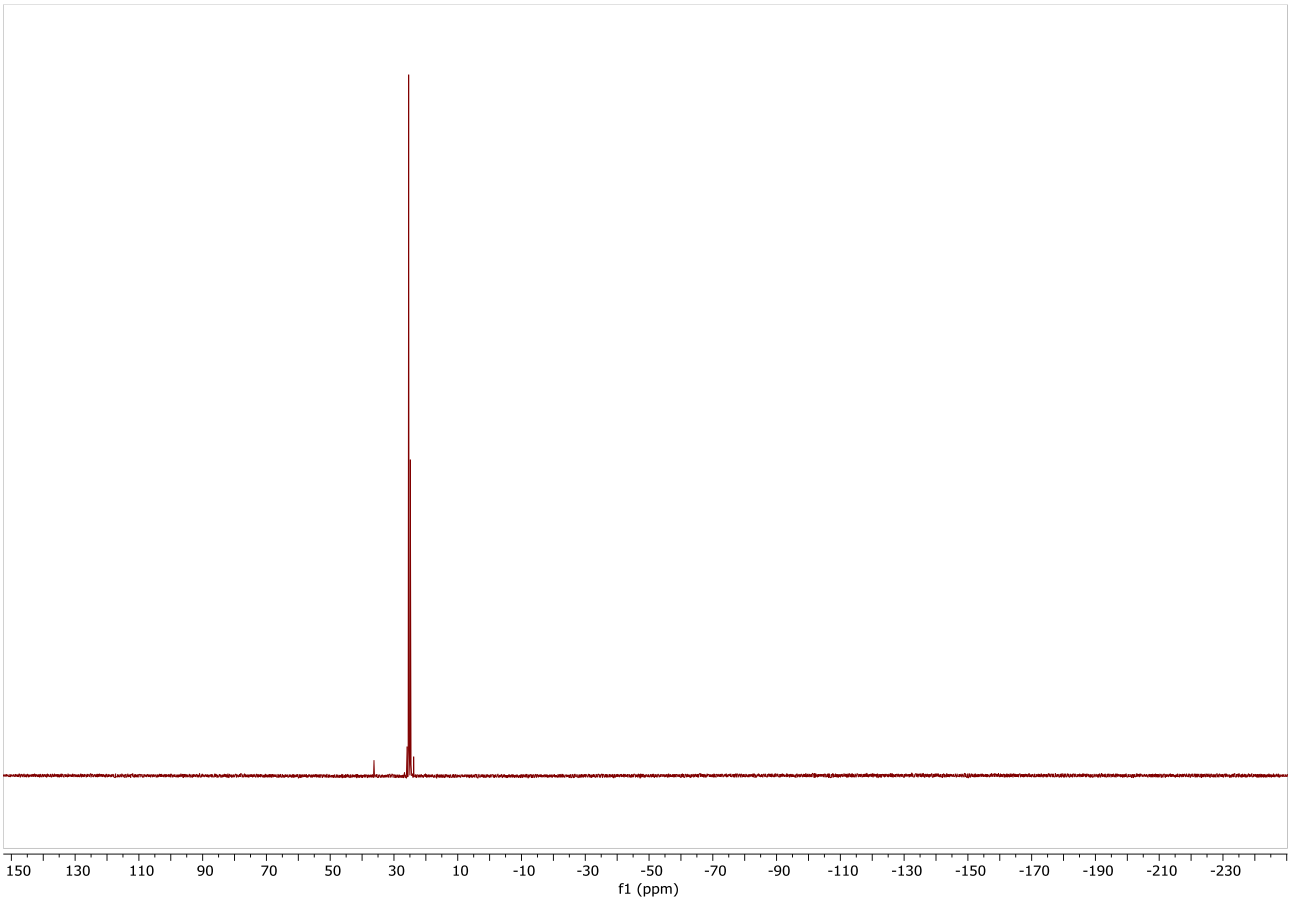

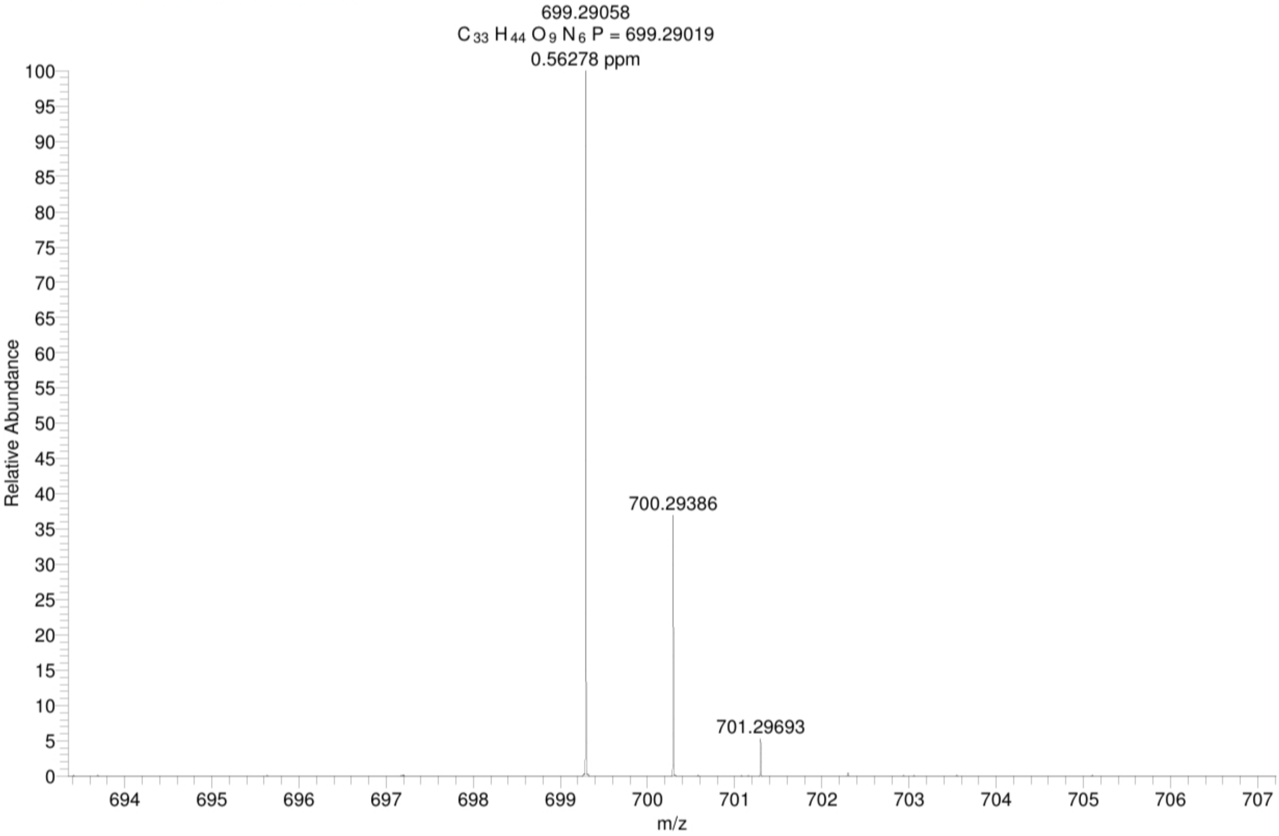

Fig. S31. ^{31}P NMR of compound **21c** (at rt) in DMSO (top) and high resolution mass spectrum (HRMS, bottom) of compound **21c**.

Bis-(L-phenylalaninate ethyl ester) prodrug of (2-(2-(guanin-9-yl)-2-(2-hydroxyethoxy)ethoxy)ethyl) phosphonic acid (21d)

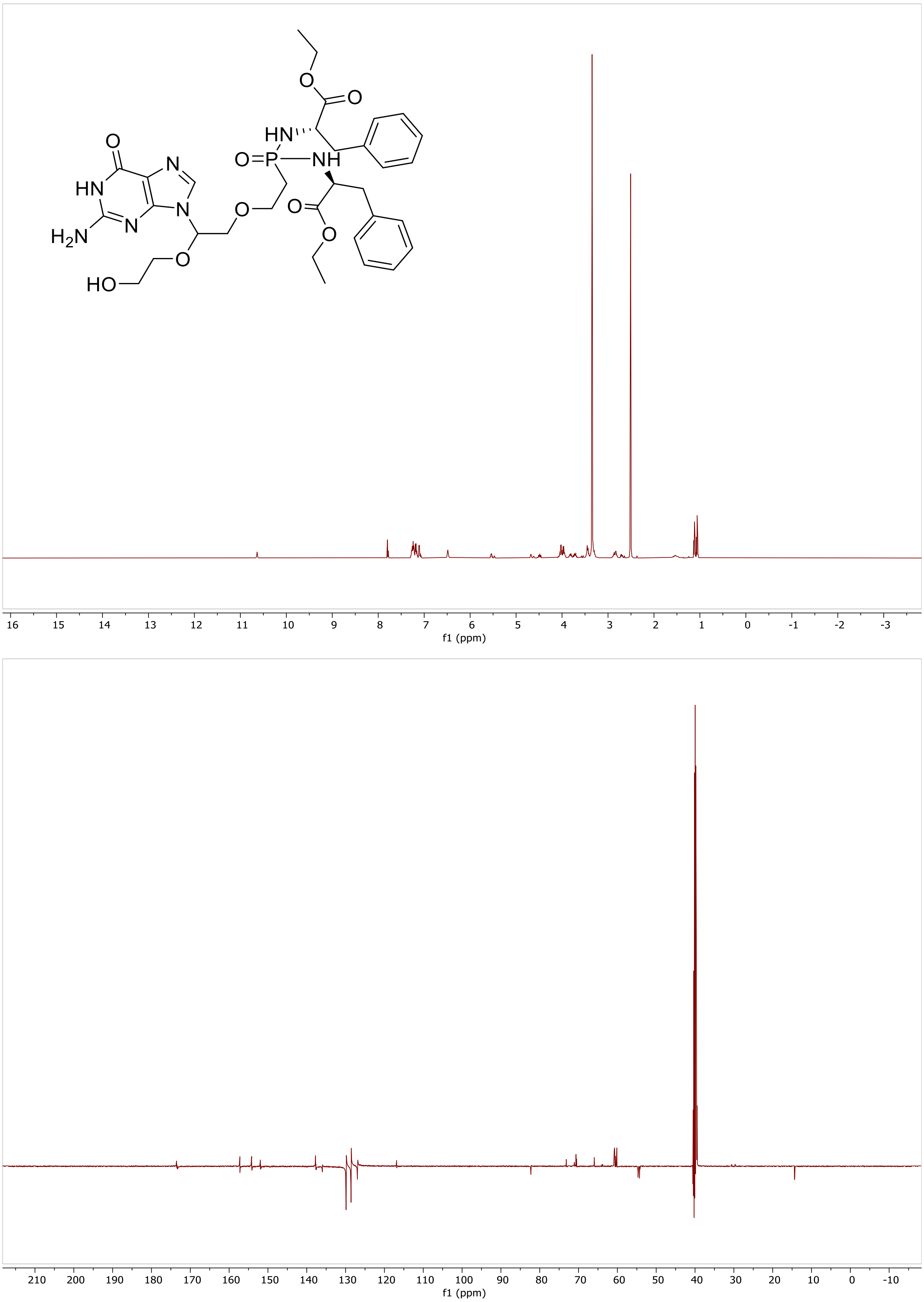

Fig. S32. ^{1}H (top) and ^{13}C (bottom) NMR spectra of compound **21d** (at rt) in DMSO.

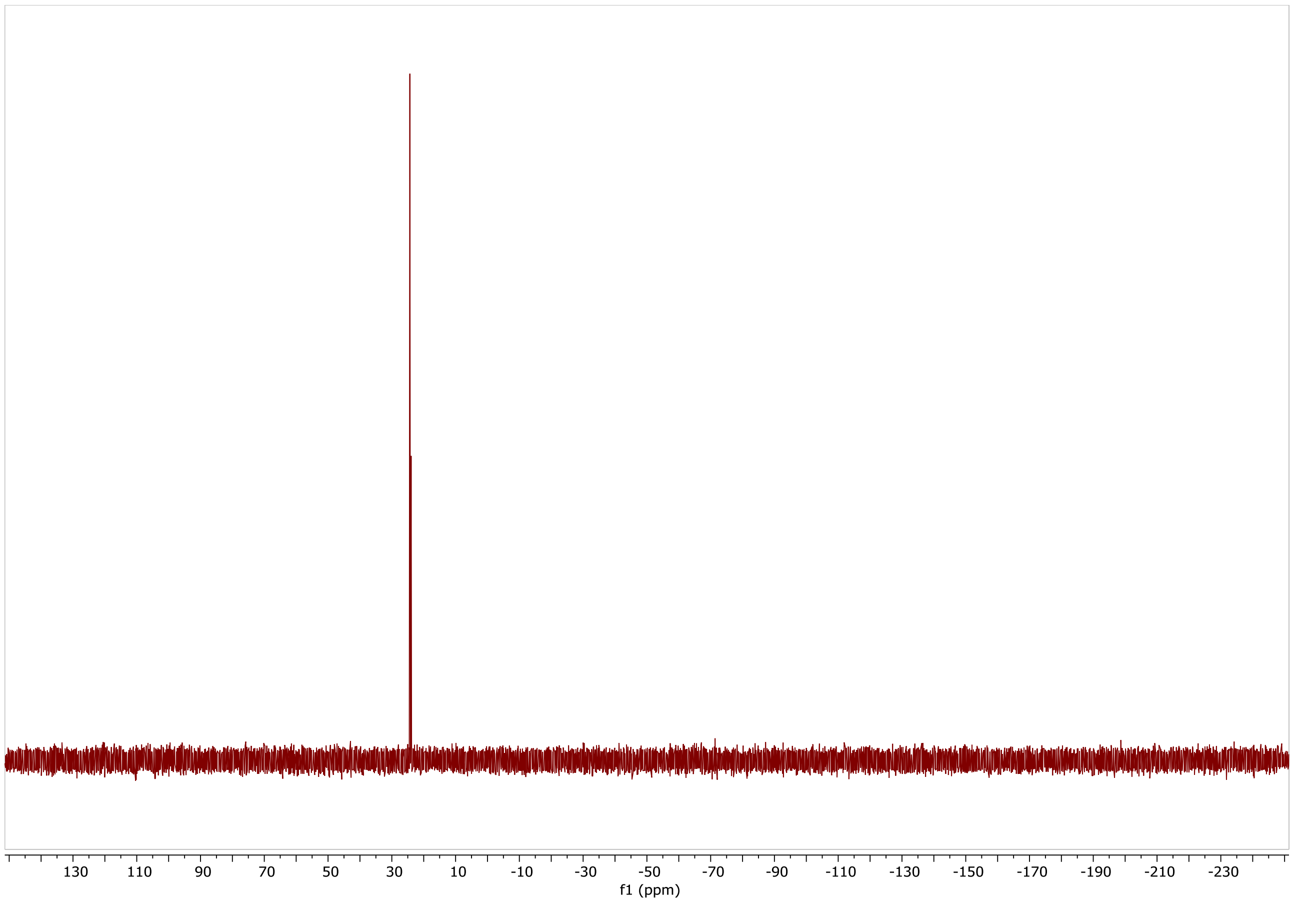

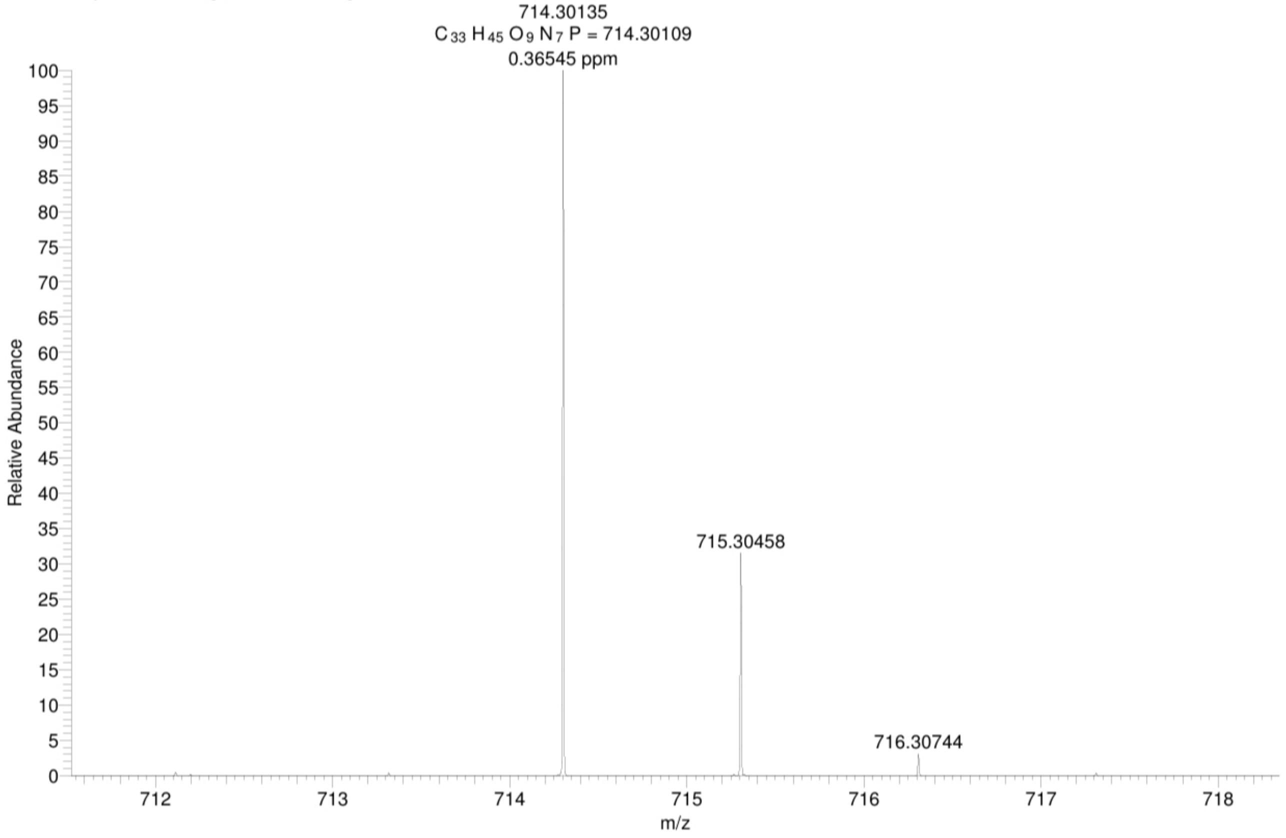

Fig. S33. ^{31}P NMR of compound **21d** (at rt) in DMSO (top) and high resolution mass spectrum (HRMS, bottom) of compound **21d**.

Bis-(L-phenylalaninate ethyl ester) prodrug of ((3-(3-hydroxypropoxy)-3-(hypoxanthin-9-yl)propoxy)methyl) phosphonic acid (21e)

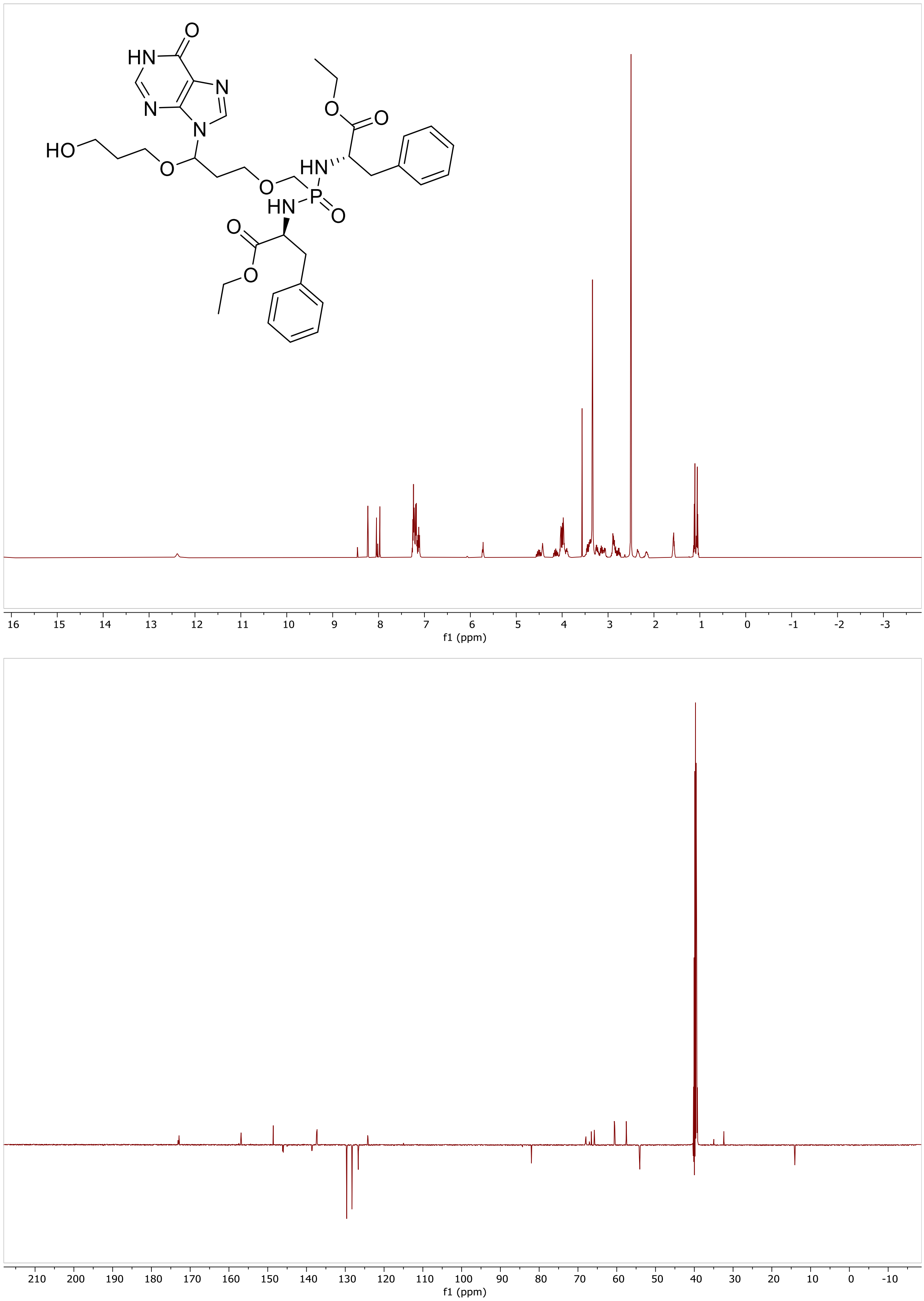

Fig. S34. ^{1}H (top) and ^{13}C (bottom) NMR spectra of compound **21e** (at rt) in DMSO.

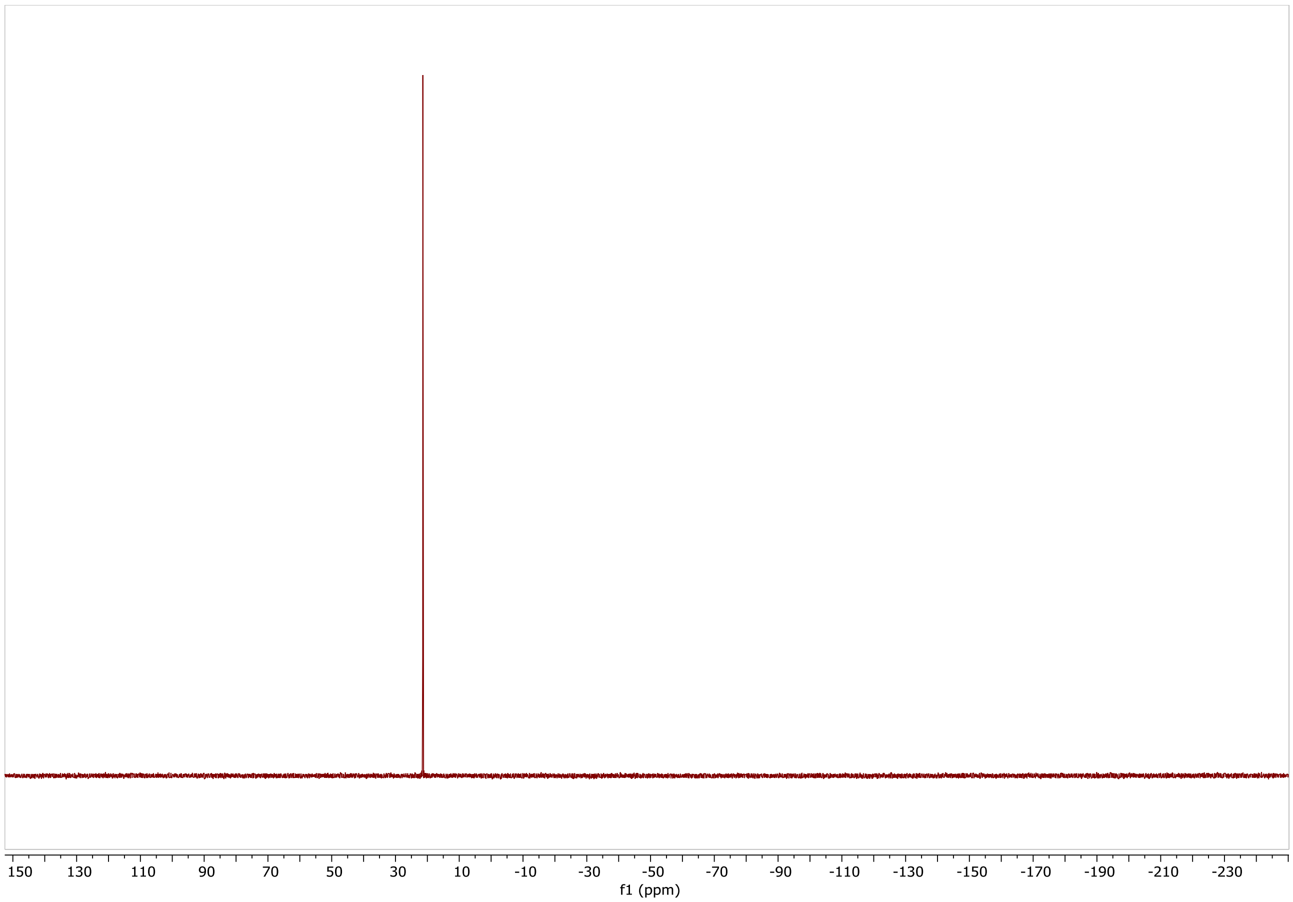

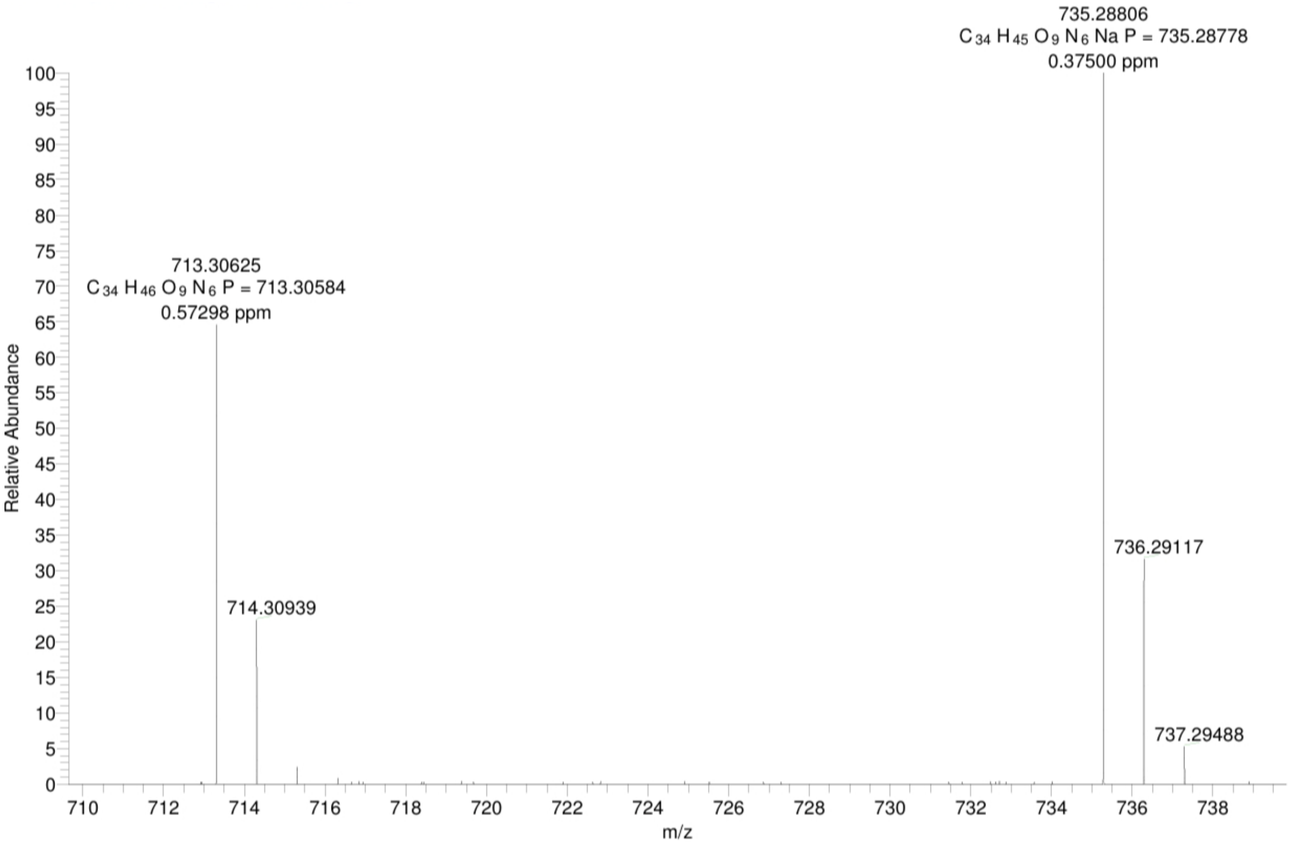

Fig. S35. ^{31}P NMR of compound **21e** (at rt) in DMSO (top) and high resolution mass spectrum (HRMS, bottom) of compound **21e**.

Bis-(L-phenylalaninate ethyl ester) prodrug of ((3-(3-hydroxypropoxy)-3-(guanin-9-yl)propoxy)methyl) phosphonic acid (21f)

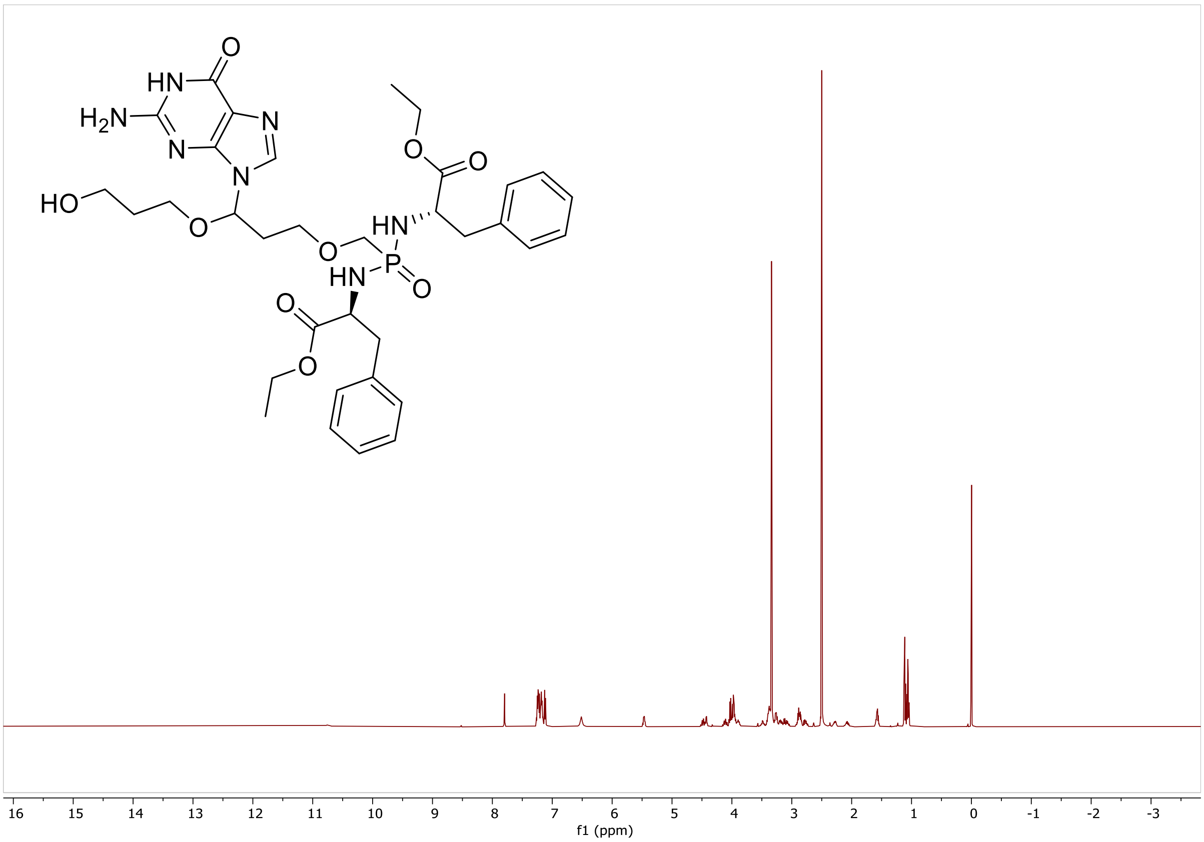

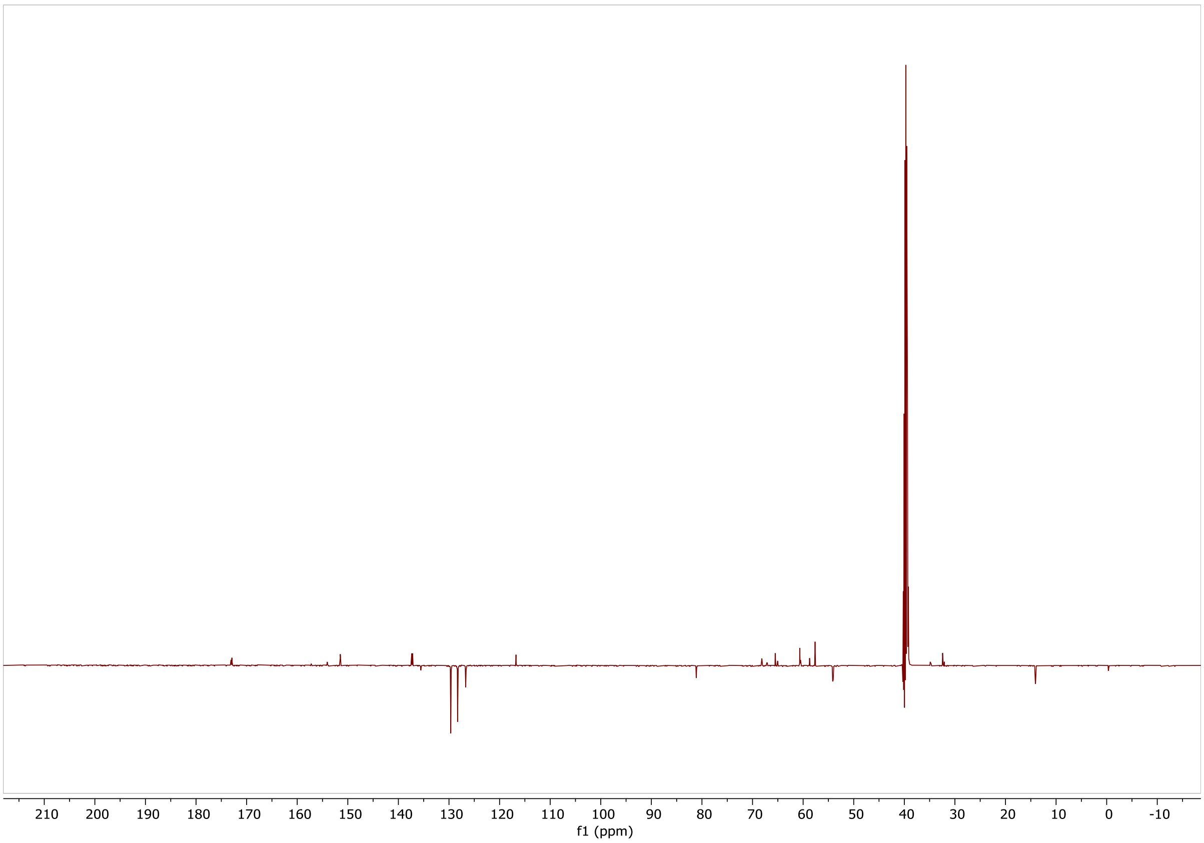

Fig. S36. ^{1}H (top) and ^{13}C (bottom) NMR spectra of compound **21f** (at rt) in DMSO.

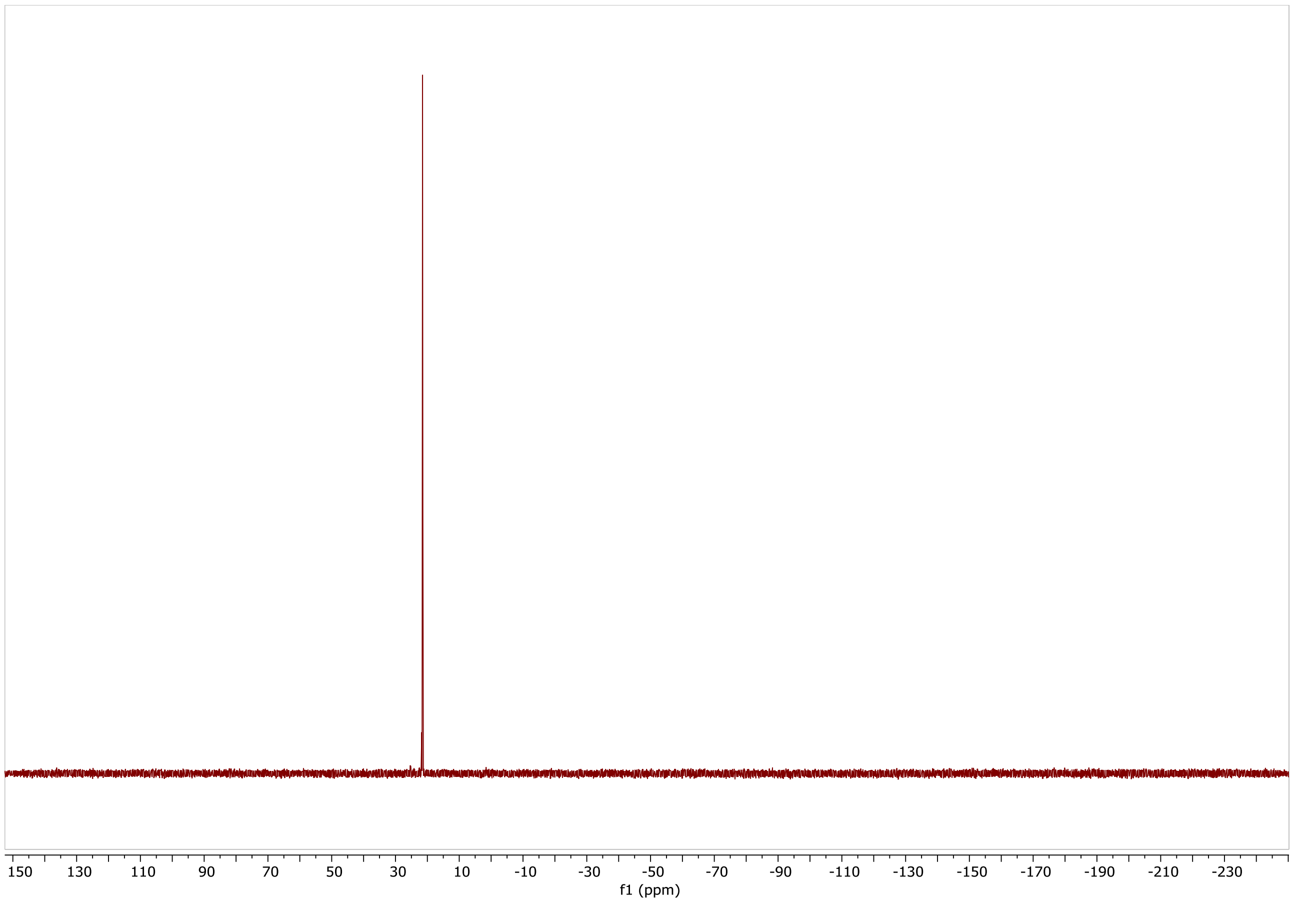

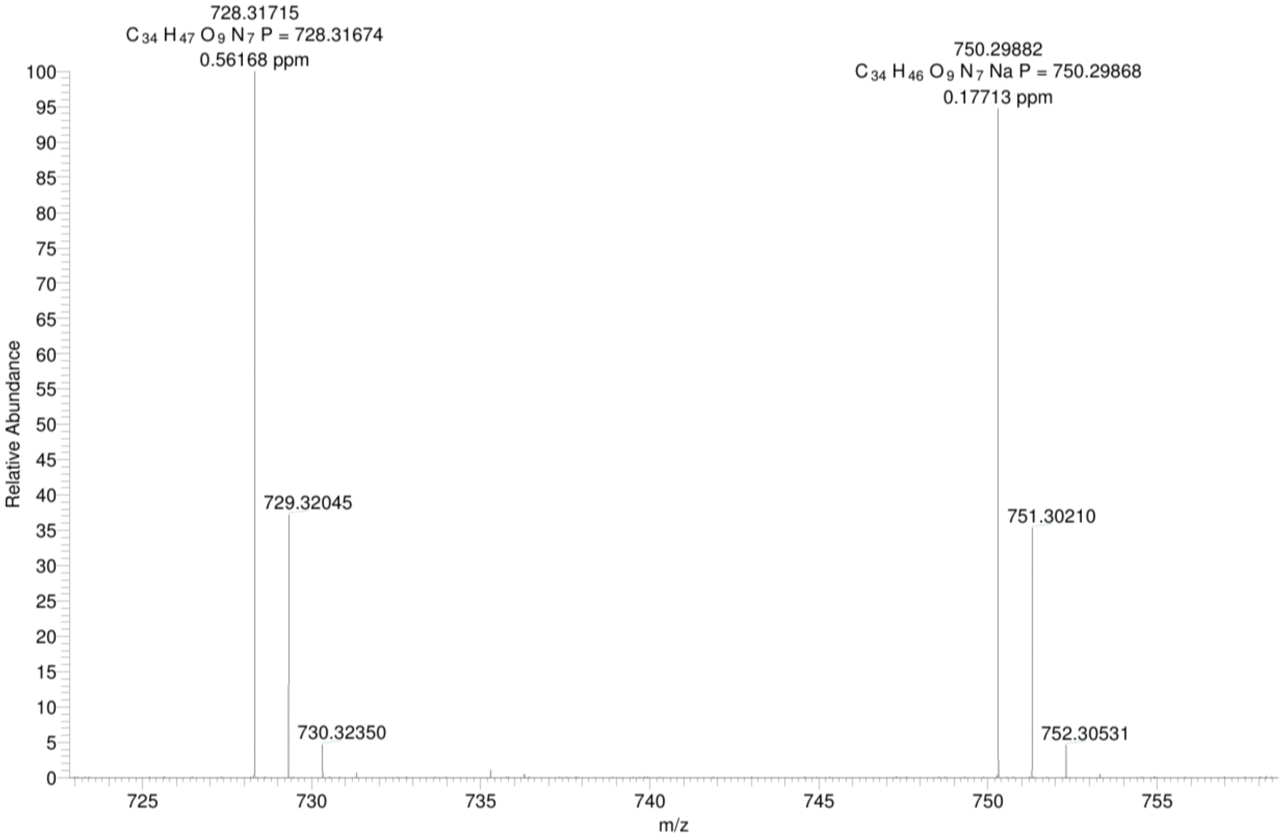

Fig. S37. ^{31}P NMR of compound **21f** (at rt) in DMSO (top) and high resolution mass spectrum (HRMS, bottom) of compound **21f**.

Bis-(L-phenylalaninate ethyl ester) prodrug of ((3-(3-hydroxypropoxy)-3-(4-oxo-4,5-dihydro-1*H*-pyrazolo[3,4-d]pyrimidin-1-yl)propoxy)methyl) phosphonic acid (21g)

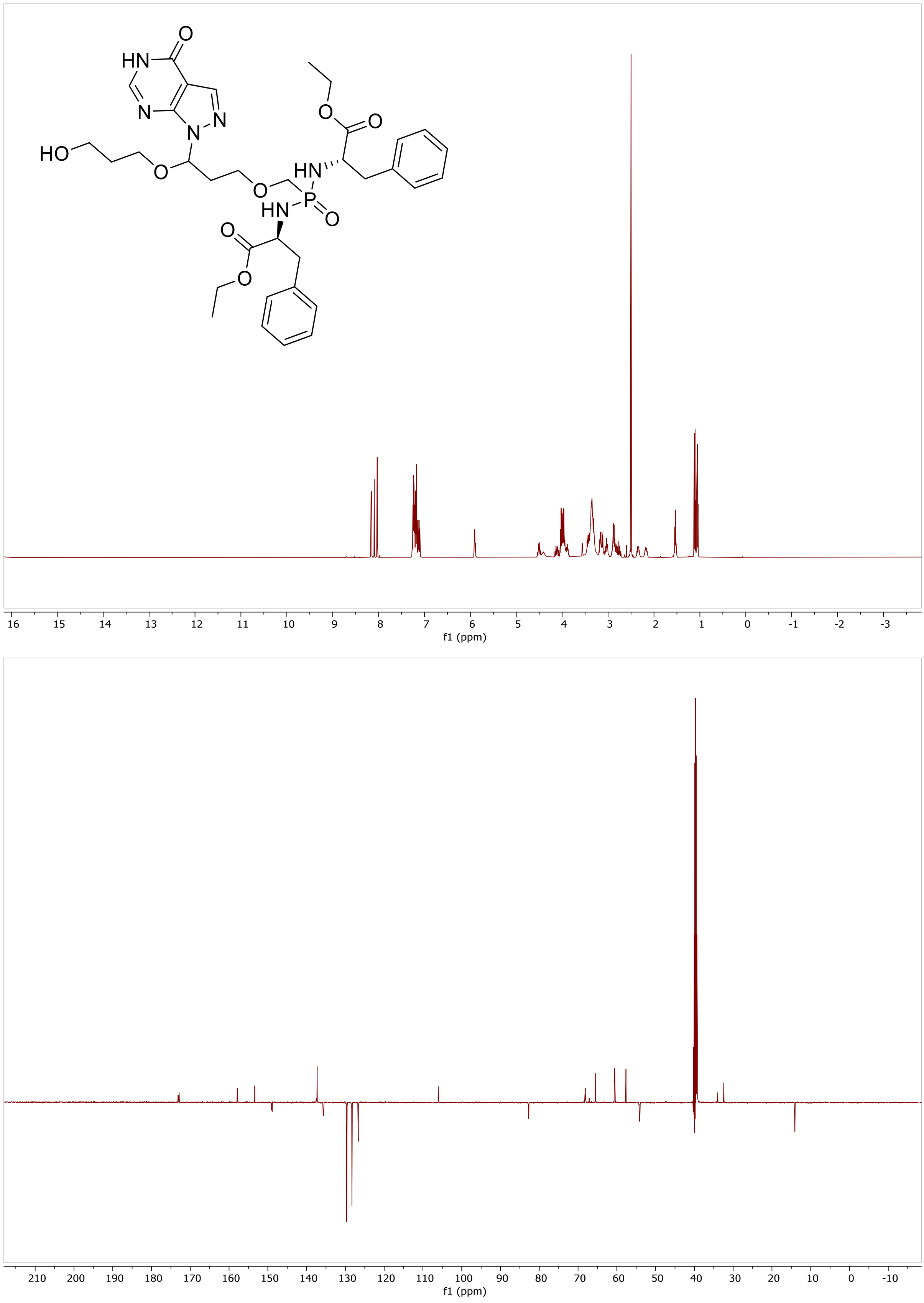

Fig. S38. ^{1}H (top) and ^{13}C (bottom) NMR spectra of compound **21g** (at rt) in DMSO.

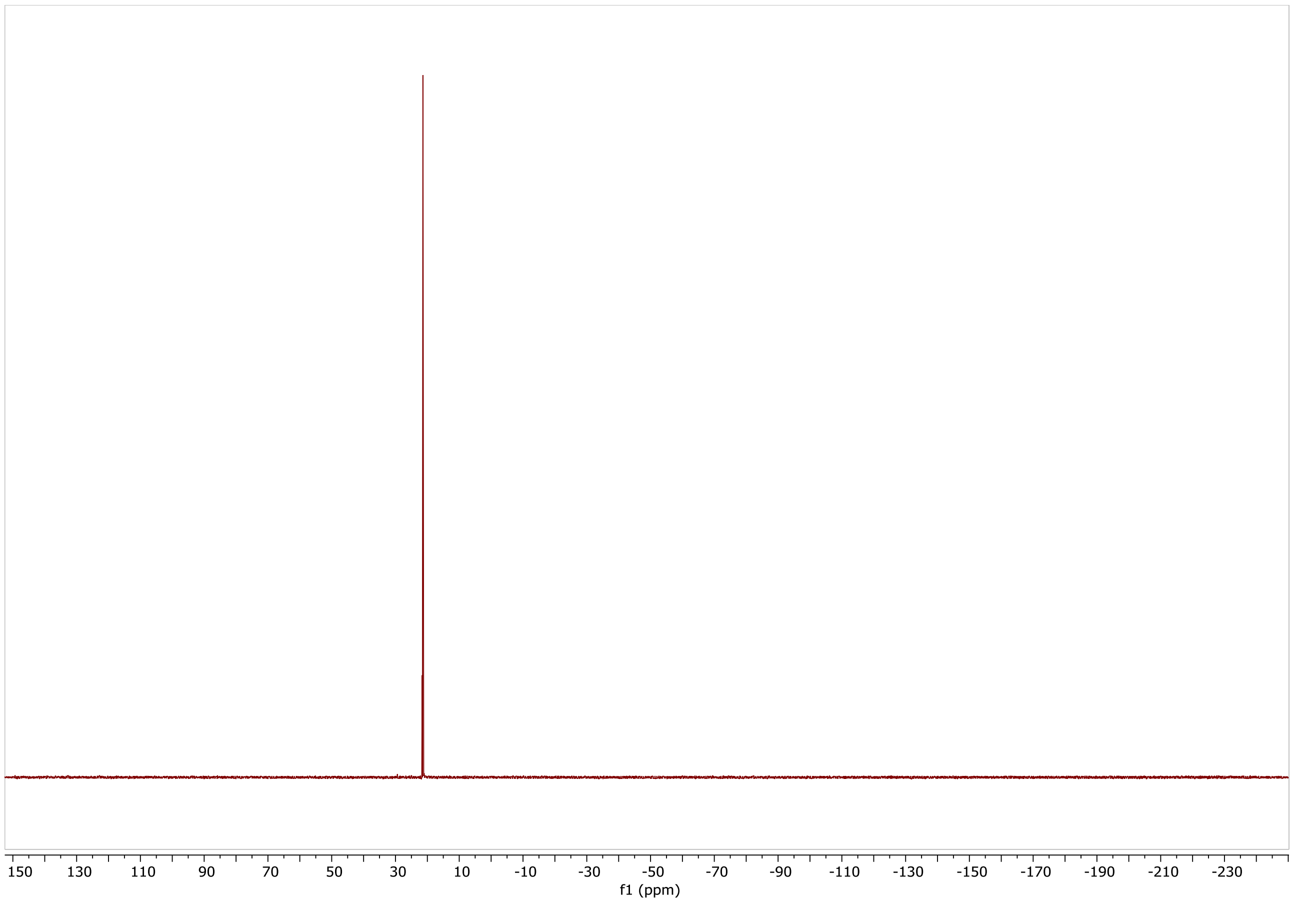

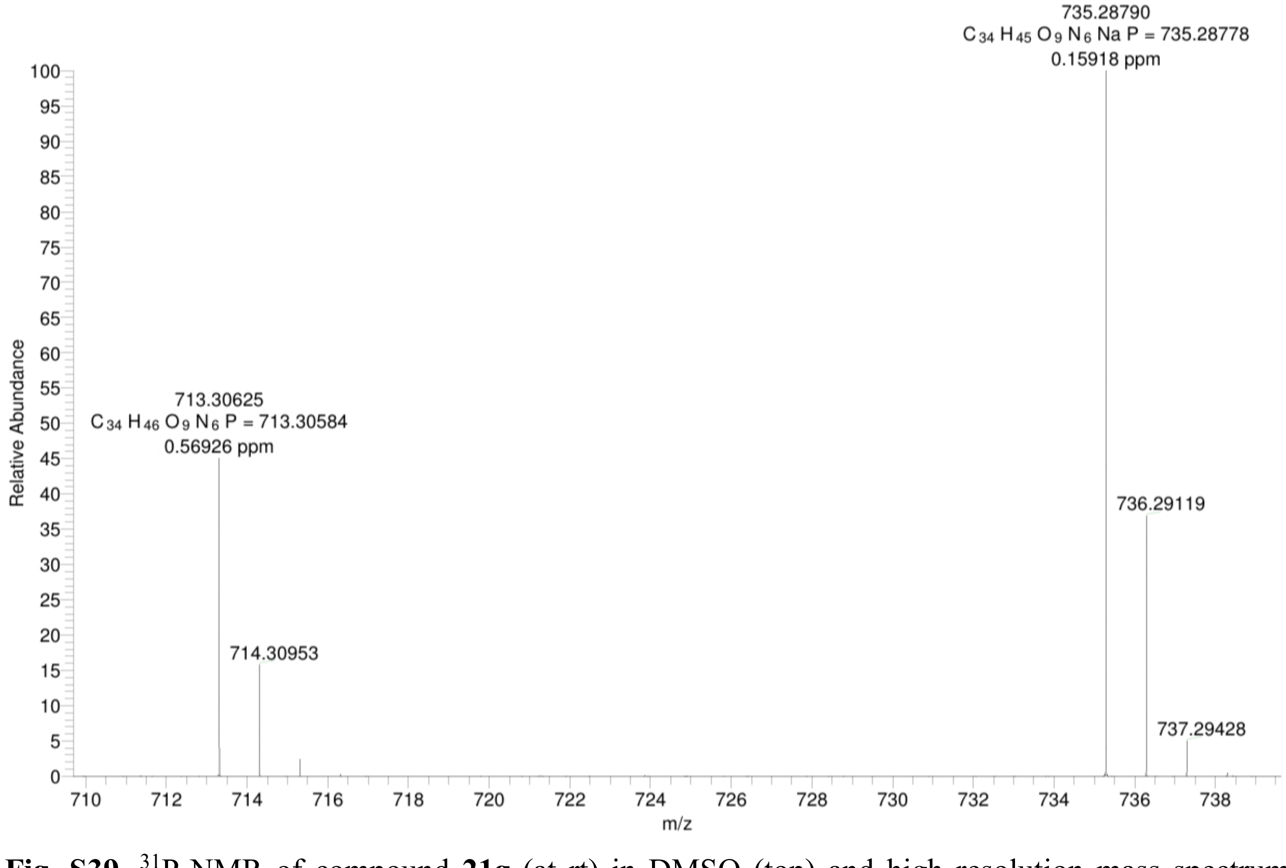

Fig. S39. ^{31}P NMR of compound **21g** (at rt) in DMSO (top) and high resolution mass spectrum (HRMS, bottom) of compound **21g**.

Sodium salt of (2-(2-(guanin-9-yl)-2-ethoxyethoxy)ethyl)phosphonic acid (25)

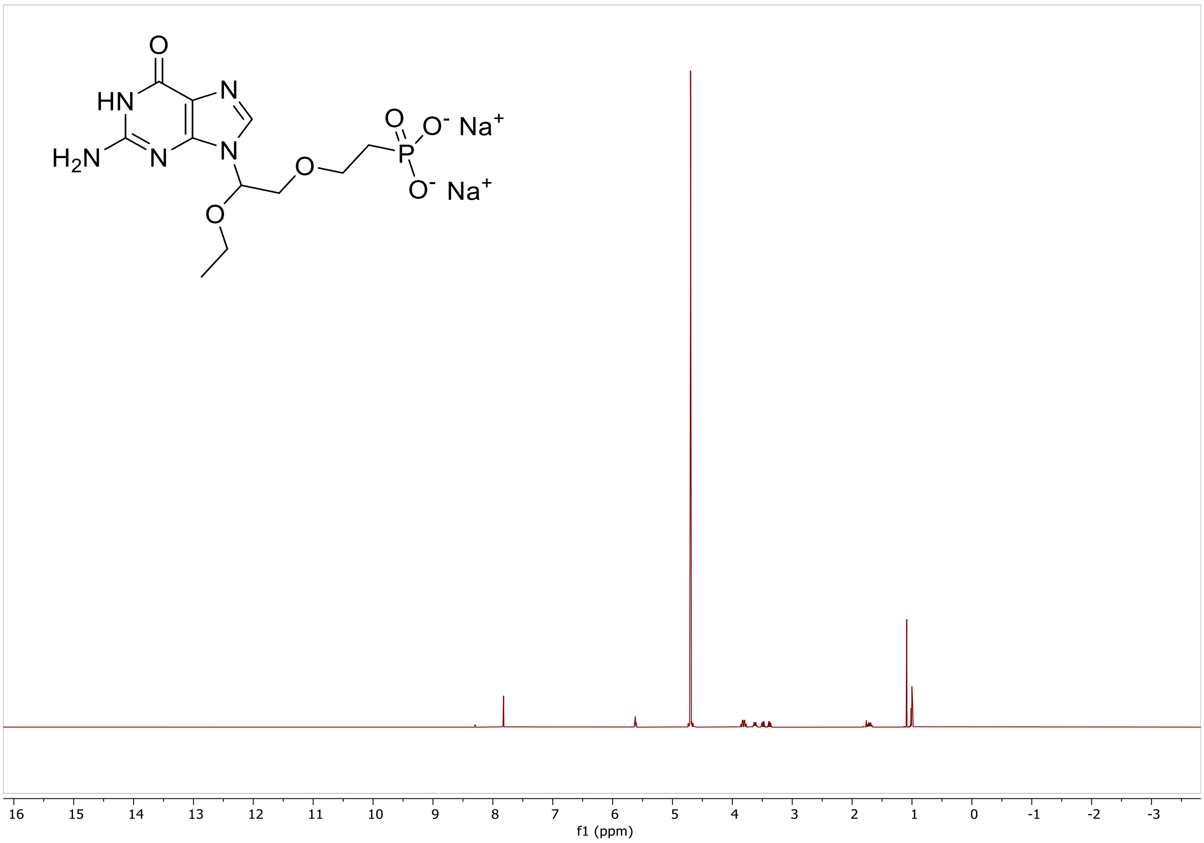

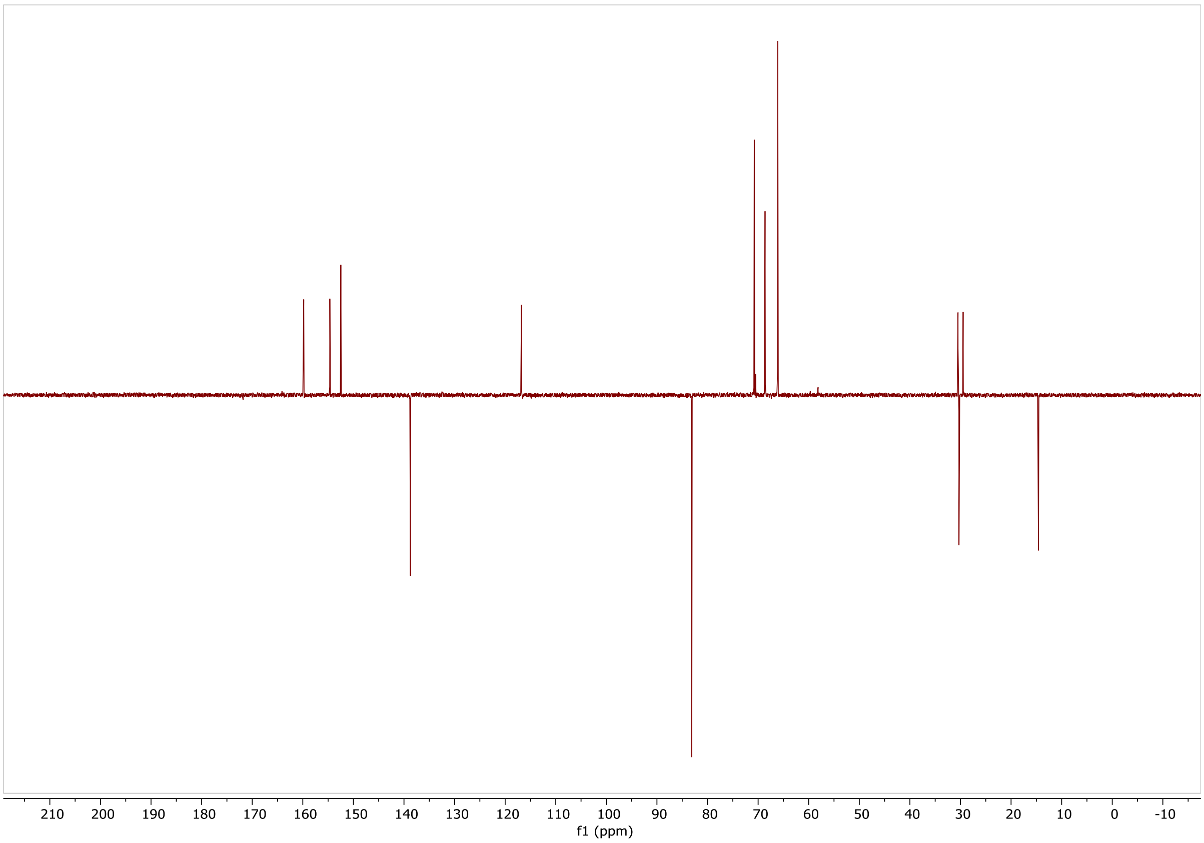

Fig. S40. ^{1}H (top) and ^{13}C (bottom) NMR spectra of compound **25** (at rt) in D_2O containing 0.1% of *terc*-butyl alcohol as an internal standard.

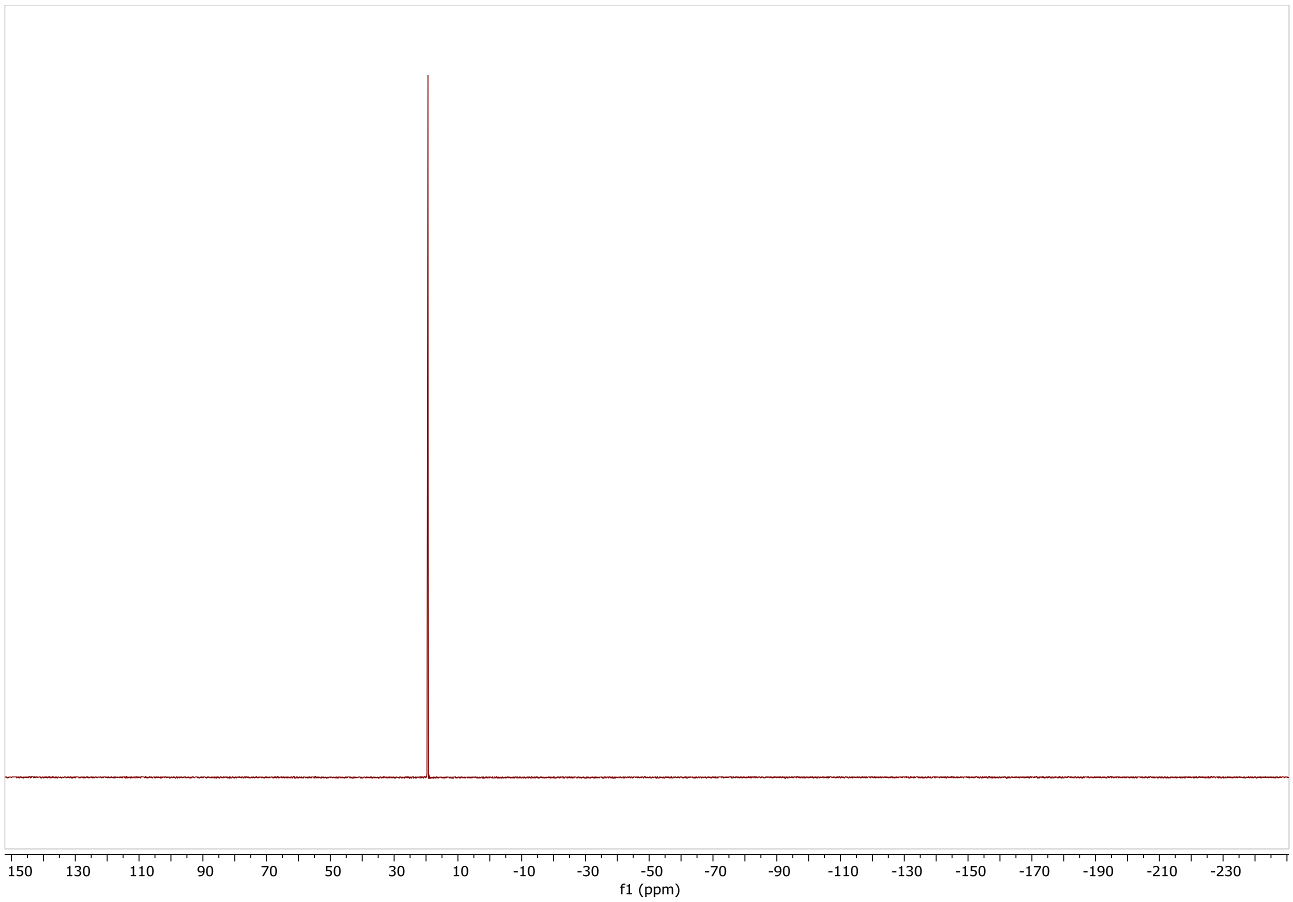

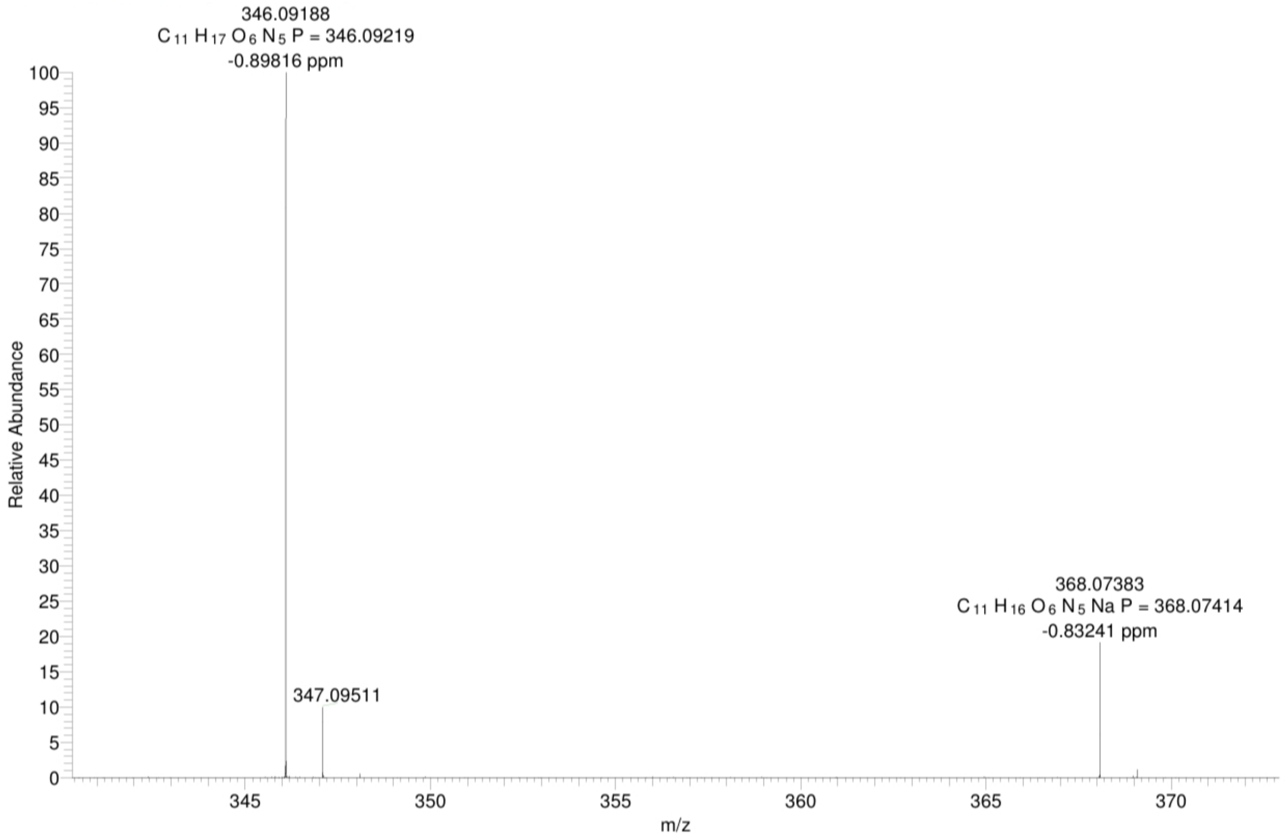

Fig. S41. ^{31}P NMR of compound **25** (at rt) in D_2O containing 0.1% *terc*-butyl alcohol as an internal standard (top) and high resolution mass spectrum (HRMS, bottom) of compound **25**.
